# Phenotypic correlates of the lianescent growth form: a review

**DOI:** 10.1093/aob/mct236

**Published:** 2013-10-29

**Authors:** Tomasz P. Wyka, Jacek Oleksyn, Piotr Karolewski, Stefan A. Schnitzer

**Affiliations:** 1 Adam Mickiewicz University, Department of Biology, Institute of Experimental Biology, Laboratory of General Botany, Umultowska 89, 61-614 Poznań, Poland; 2 Polish Academy of Sciences, Institute of Dendrology, Parkowa 5, 62-035 Kórnik, Poland; 3 University of Minnesota, 1530 Cleveland Avenue North, St. Paul, MN 55108, USA; 4 University of Wisconsin-Milwaukee, Department of Biological Sciences, PO Box 413, Milwaukee, WI 53201, USA

**Keywords:** Climbers, vines, lianas, growth forms, plant functional types, ecological strategy

## Abstract

**Background:**

As proposed by Darwin, climbers have been assumed to allocate a smaller fraction of biomass to support organs in comparison with self-supporting plants. They have also been hypothesized to possess a set of traits associated with fast growth, resource uptake and high productivity.

**Scope:**

In this review, these hypotheses are evaluated by assembling and synthesizing published and unpublished data sets from across the globe concerning resource allocation, growth rates and traits of leaves, stems and roots of climbers and self-supporting species.

**Conclusions:**

The majority of studies offer little support for the smaller allocation of biomass to stems or greater relative growth rates in climbers; however, these results are based on small sized (<1 kg) plants. Simulations based on allometric biomass equations demonstrate, however, that larger lianas allocate a greater fraction of above-ground biomass to leaves (and therefore less biomass to stems) compared with similar sized trees. A survey of leaf traits of lianas revealed their lower average leaf mass per area (LMA), higher N and P concentration and a slightly higher mass-based photosynthetic rate, as well as a lower concentration of phenolic-based compounds than in woody self-supporting species, consistent with the specialization of lianas towards the fast metabolism/rapid turnover end of the global trait spectra. Liana stems have an efficient hydraulic design and unique mechanical features, while roots appear to penetrate deeper soil levels than in trees and are often able to generate hydraulic pressure. Much remains to be learned, however, about these and other functional specializations of their axial organs and the associated trade-offs. Developmental switches between self-supporting, searcher and climbing shoots within the same individual are a promising field of comparative studies on trait association in lianas. Finally, some of the vast trait variability within lianas may be reduced when species with different climbing mechanisms are considered separately, and when phylogenetic conservatism is accounted for.

## INTRODUCTION

Plant ecologists have long sought to classify the bewildering variety of plant species into a manageable number of functional types that could be used to represent the essential characteristics of particular biomes or vegetation of geographic areas (Wright *et al.*, 2005*b*; Lavorel *et al.*, 2007). Commonly recognized functional types include trees, shrubs, herbs, graminoids and climbers. Functional types have been useful in modelling the processes of succession and, especially in recent years, the responses of vegetation to changing climatic factors (Reich *et al.*, 2003; Reich and Oleksyn, 2004; Wright *et al.*, 2004*b*; Schnitzer and Bongers, 2011). The usefulness and definition of functional types has been debated, but there is much support for significant differences in key traits among plants of different functional types (e.g. Wright *et al.*, 2005*a*, *b*; Asner and Martin, 2012).

Climbers, also known as vines, are plants rooted in the soil that are incapable of autonomuos vertical growth above a certain height and must rely on external support. Commonly, a distinction is made between herbaceous vines and lianas, the latter exhibiting a significant degree of woodiness (Gentry, 1991). These plants have long attracted the interest of botanists because of their peculiar climbing mechanisms (Darwin, 1865; Isnard and Silk, 2009), anatomical modifications (Schenck, 1893; Carlquist, 1991; Bowling and Vaughn, 2009), biomechanical characteristics (Rowe *et al.*, 2004), extreme stem hydraulic capacities (Gartner *et al.*, 1990; Ewers *et al.*, 1991), extraordinary developmental plasticity (Lee and Richards, 1991) and other unusual but easily identifiable characteristics associated with this growth form. Much knowledge of climbing plant biology comes from the study of climbing crop species, such as grape, kiwi, hop, bean, peas and cucumber. Trait combinations of such plants have probably been inflenced by past selection for yield enhancement under cultivation, thus reflecting agricultural, rather than ecological context (e.g. Lovisolo and Schubert, 1998). In fact, studies of the functional biology and ecology of wild climbers appear to lag behind those of other functional types (Schnitzer and Bongers, 2002; Bowling and Vaughn, 2009).

Lianas constitute a major functional type in temperate, and especially in tropical zones. Liana research has recently been stimulated by their increasing presence and even dominance in disturbed vegetation and the discoveries of the multifaceted role they play in forest dynamics (Schnitzer and Bongers, 2002, 2011; Wright *et al.*, 2004*a*; Selaya *et al.*, 2007; Schnitzer *et al.*, 2012; Yong *et al.*, 2012) as well as by the weedy nature and economic problems caused by some of the species (Putz, 2011). As the development of meta-analytical tools has led to synthetic works and global overviews of various functional groups of plants (e.g. Wright *et al.*, 2005*a*, *b*; Poorter *et al.*, 2012), it has become increasingly clear that a number of questions still need to be answered with respect to biological characterization of lianas as a group. Whereas external morphology and the underlying anatomical structure as well as the physiology of the climbing mechanisms have been extensively investigated and reviewed (Isnard and Silk, 2009), much less attention had been paid to modifications of other functional traits that are not directly related to climbing but nevertheless constitute important components of the climbing strategy. This review addresses such functional characteristics of wild lianas while focusing on whole-plant resource allocation, growth rate and some organ-specific structural and functional features of leaves, stems and roots.

## ALLOCATION TO SUPPORT VS. LEAVES

In a recent review (Poorter *et al.*, 2012), we identified major gaps in our understanding of the biomass allocation strategy in climbers. Plants in this group have slender, flexible stems and use neighbouring plants or other objects for mechanical support, often to the detriment of their hosts (Schnitzer and Bongers, 2002). They have therefore often been termed ‘structural parasites’ (Stevens, 1987). Their distinct habit inspired Darwin (1865) to formulate the hypothesis that climbers economize on costs of constructing stems, which allows them to form a larger and more competitive leaf canopy. In Darwin's (1865) words: ‘Plants become climbers, in order, it may be presumed, to reach light, and to expose a large surface of leaves to its action and to that of the free air. This is effected by climbers with wonderfully little expenditure of organized matter, in comparison with trees, which have to support a load of heavy branches by a massive trunk’. Using the contemporary ecophysiological terminology, climbers should thus have a lower stem mass ratio (i.e. ratio of stem dry mass to whole-plant biomass) and greater leaf mass and area ratios (respectively, ratios of total leaf mass or leaf area to whole-plant mass) than self-supporting plants. In spite of numerous supporting observations for the differential biomass allocation pattern in climbers and erect plants, few explicit tests of Darwin's hypothesis have been attempted. The existing studies can be sorted into three distinct categories: (1) formal whole-plant allocation analyses of lianas and erect species; (2) comparative shoot-level studies of organ allocation ratios; and (3) studies in which lianas were either given or denied support. They are then best grouped into studies using juvenile or small sized individuals vs. those that used large, canopy-reaching individuals.

### Juvenile or pre-climbing stage

Intrinsic allocation patterns are usually investigated under uniform, preferably controlled, conditions. Because of space and cultivation time restrictions, however, available data are biased towards herbaceous species and juvenile life stages of woody species. For example, a growth chamber study of British woody plant seedlings demonstrated a greater leaf area ratio in climbers and scramblers than in shrubs or trees; however, on converting to leaf mass ratios, the values were similar in all three life forms (0·54 for climbers and shrubs, 0·51 for trees; data on allocation to stems were not provided; Cornelissen *et al.*, 1996). Within the woody genus *Bauhinia* (Fabaceae), pot-cultured seedlings of two light-demanding climbing species allocated a greater portion of biomass to stems (after becoming scandent) than did seedlings of two tree species (Cai *et al.*, 2007). In contrast, *Bauhinia aurea*, a single species of slow-growing, shade-tolerant liana included in that study, allocated less biomass to stems than did the arborescent *Bauhinias*. All lianescent *Bauhinias* had greater whole-plant leaf mass ratios than the arborescent species, accompanied by lower root mass rather than stem mass (Cai *et al.*, 2007). These examples, to our knowledge, are the only data sets that compared whole-plant allocation patterns between lianas and other growth forms under controlled (Cornelissen *et al.*, 1996) or semi-controlled (Cai *et al.*, 2007) conditions.

Although evaluation of Darwin's climber biomass allocation hypothesis requires that allocation to plant organs is expressed against the entire plant biomass, for practical reasons field studies of lianas tend to restrict themselves to measuring above-ground biomass fractions. At a Bolivian Amazon site, leaf to stem mass ratios in young regenerating lianas at the self-supporting stage were not different from those in surrounding tree saplings (Selaya *et al.*, 2007). Self-supporting lianas and trees face similar constraints and have similar stem architecture, and thus are not likely to differ in their allocation patters at this ontogenetic state. However, in an experimental garden in The Netherlands, leaf/stem mass ratios were also similar between the scandent liana *Lonicera peryclimenum* and the shrub *L. xylosteum* (Caprifoliaceae; During *et al.*, 1994).

Many liana species exhibit a marked plasticity in response to the availability of support structures (Rowe and Speck, 2004). In the absence of a support trellis, a more or less erect, bushy or scrambling form may be produced. This property offers an additional opportunity for evaluation of the various characteristics of climbing vs. non-climbing forms within the same species, i.e. without the confounding interspecific differences. Assuming the adaptive nature of such plasticity, traits expressed in the climbing form may be interpreted as functionally associated with the climbing habit. With respect to allocation, a common garden comparison of 2-year-old shrub and climbing forms of *Toxicodendron radicans* (Anacardiaceae) revealed a greater contribution of stems to above-ground plant biomass in climbers and similar leaf mass ratios between the forms (Gartner, 1991). Likewise, in pot-grown 2-year-old *Wisteria floribunda* (Fabaceae), availability of support caused an increase in relative allocation to stems and a decrease in allocation to roots, but no effect on allocation to leaves (Sakai and Suzuki, 1999). Observations of climbers in the natural environment and the results of some controlled studies, however, suggest a stimulatory effect of support availability on shoot extension and, often, on whole-plant growth (Sakai and Suzuki, 1999). The latter authors reported both stem length and dry mass increases in staked *W. floribunda* plants, as well as a positive effect of trellis height on plant growth. Thus, it is important to account for plant size when comparing allocation ratios between supported and unsupported plants. Results of support availability experiments thus far have not demonstrated reduced support costs in lianas. Only a single study, using *Hedera helix* (Araliaceae), found a slightly increased allocation to leaves at the expense of stems in vertically growing shoots in a highly artificial experimental set-up (Frey and Frick, 1987).

Although all of the above studies failed to support Darwin's hypothesis unequivocally, they only included small sized (**<**1000 g dry weight**)** individuals and therefore did not consider the potential differentiation of allocation ratios between growth forms that becomes apparent only in larger individuals. A size-dependent decrease in biomass allocation to stems is especially likely in those liana species in which young saplings are self-supporting and whose stem structure undergoes a dramatic transformation after the liana begins to climb (Ewers *et al.*, 1991; Angyalossy *et al.*, 2012, 2013).

### Mature climbers

For most plants, the accumulation of biomass accompanying shoot elongation causes an increased requirement for support (Rowe and Speck, 2004). As proposed by Darwin, and suggested by many casual observations, this cost in lianas might be reduced by lianas being supported by stems of neighbouring plants. Surprisingly few data sets on adult lianas exist that can be used to test this hypothesis.

The only published whole-plant allocation report that considers mature lianas is on the naturally grown reproductive *Hydrangea petiolaris* (Hydrangeaceae). Surprisingly, stems in this species accounted for a much larger fraction of biomass than in three shrubby *Hydrangea* species growing at the same site, whereas leaf mass fractions were not different between the growth forms (Kaneko and Homma, 2006). However, this species is a temperate plant that grows as a root climber (a somewhat rare climbing strategy in tropical areas, where the vast majority of liana species occur). Furthermore, to compare organ mass fractions in different species meaningfully, one should account for potentially allometric relationships between plant size and allocation ratios (Poorter and Sack, 2012). Ideally, plants should be compared at the same body size, whereas in Kaneko and Homma's 2006 study the lianescent *H. petiolaris* plants were 1–2 orders of magnitude larger than the shrubby species. As an alternative, slopes of log–log relationships between plant and organ mass with at least some overlap of size ranges may be studied. An analysis of this kind showed that plant size-corrected stem mass and leaf mass ratios in climbing *H. petiolaris* were not statistically different from those in the three shrubby congeners (Kaneko and Homma, 2006).

The majority of available data on biomass allocation in large sized lianas are limited to above-ground parts (Putz, 1983; Teramura *et al.*, 1991). Work on an invasive, large herbaceous vine *Pueraria lobata* (Fabaceae) by Wechsler (1977) cited by Teramura *et al.* (1991) indicated that leaf area supported by a unit of stem mass in this species is greater by at least an order of magnitude than in temperate trees. Lianas in a Venezuelan ‘tierra firme’ forest were shown to have a larger total leaf mass than trees of a similar basal stem area, except for individuals with the smallest (i.e. under approx. 20 cm^2^) basal areas that were often still self-supporting (Putz, 1983). The slope of the relationship of leaf mass vs. stem basal area was six times larger in lianas than in trees, indicating that as lianas reach and explore the canopy the leaf mass to stem diameter ratio rapidly increases. At another Amazonian forest site, 1 cm and 10 cm diameter liana stems supported five and four times greater leaf mass then did trees of similar stem diameters (Gerwing and Farias, 2000). Finally, biomass alocation to stem tissue in terminal twigs sampled in canopies of Madagascan forest was twice as large in trees as in lianas, showing a lower cost of canopy space acquisition in lianas (Kazda *et al.*, 2009).

**Fig. 1.**
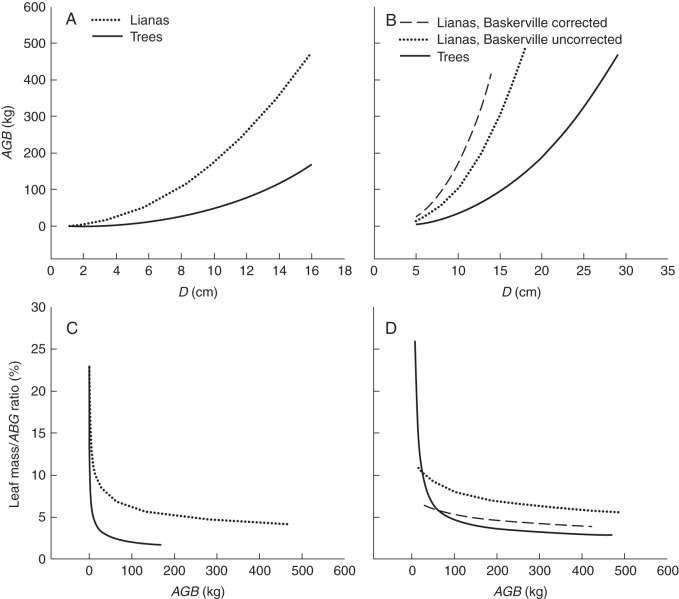
Simulated relationships between above-ground dry plant biomass (*AGB*) and stem diameter (*D*) measured at 1·30 m above the root (A, B), and between the leaf mass ratio and *AGB* (C, D) for Amazonian lianas and trees based on published equations. A and C utilized equations used by Gerwing and Farias (2000): *AGB* = 10^0·07+^^2·17×log (^^*D*^^)^ for lianas and *AGB* = 0·603 × e^−1^^·754+2^^·665×ln(^^*D*^^)^ for trees with *D* <20 cm; the latter equation originally proposed by Higuchi *et al.* (1997). Leaf mass in C was estimated from equations by Gerwing and Farias (2000): leaf mass = 10^−^^0·57+0^^·81×log (basal area)^ for lianas and leaf mass = 10^−1^^·26+^^0·84×log (basal area)^ for trees. B and D utilized equations used by Schnitzer *et al.* (2006): *AGB* = e^−1^^·484+2^^·657×ln(basal area)^ and *AGB* = e^−0^^·968+2^^·657×ln(basal area)^ for lianas (the latter equation with Baskerville-corrected intercept) and *AGB* = e^−2^^·00+2^^·42×ln(^^*D*^^)^ for trees with *D* <20 cm; the latter originally proposed by Chave *et al.* (2001). Leaf mass in D was estimated from equations by Putz (1983): leaf mass *=* 0·109 × (basal area) – 0·376 for lianas and leaf mass*=* 1·368−0·018 × (basal area) for trees.

To remedy the scarcity of published information on differences in average leaf mass fractions in above-ground biomass between lianas and trees, we used published allometric equations for Amazonian trees and lianas to estimate the above-ground biomass (*AGB*) from stem diameter (*D*). Subsequently, we combined these results with published regression equations for dry leaf mass (*LM*) vs. *D* to estimate the *LM*/*AGB* ratio for each growth form (see the legend to Fig. 1 for details). In the first set of calculations, we used the allometric equation for *AGB* vs. *D* in lianas and trees under 20 cm stem diameter proposed by Gerwing and Farias (2000). The *LM* was also calculated from stem basal area using equations provided by Gerwing and Farias (2000). The second set of calculations utilized allometric equations for *AGB* vs. *D* by Chave *et al.* (2001) for trees and by Schnitzer *et al.* (2006) for lianas. The *LM* was estimated from *D* for both growth forms according to Putz (1983). Results of both independent sets of simulations suggest that lianas reach particular *AGB* levels at smaller stem diameter (Fig. 1A, B) and that, except for the smallest plants, lianas bear greater leaf mass than trees at the same above-ground plant mass (Fig. 1C, D). This difference is consistent with the smaller wood density of liana stems attributable to their lower mechanical demands and also to their more hydraulically efficient xylem (Putz, 1983). Existing allometric equations for lianas suffer from low predictability due to rarity of data for very large individuals (Schnitzer *et al.*, 2006). Nevertheless, data sets such as these, allowing comparisons of allocation to stems and leaves in lianas and trees *in situ*, are generally consistent with Darwin's hypothesis showing greater leaf mass ratios and therefore lower stem mass ratios and reduced support costs in lianas. Unfortunately, since they do not account for the biomass of roots, they still leave uncertainty as to the whole-plant allocation pattern.

## ALLOCATION TO ROOTS

In tropical forests, lianas are thought to be deeper rooted than tree species, as is evident from their high transpiration rates, high pre-dawn water potentials and low osmotic adjustment (Jackson *et al.*, 1995; Schnitzer, 2005; Zhu and Cao, 2009; Putz, 2011) as well as their greater abundance in tropical seasonally dry forests compared with rain forests (Schnitzer, 2005; Swaine and Grace, 2007; DeWalt *et al.*, 2010). For example, in a seasonal forest in Panama, lianas used a shallow water source when it was available, but used water from greater depth as the upper soil layers dried (Andrade *et al.*, 2005). The general trend for deep root penetration and tapping deep water sources is illustrated by the report by Restom and Nepstad (2004), who excavated 28 small (<140 cm tall) liana individuals of *Davilla kunthii* (Dilleniaceae) and found that their taproots reached the depth of 10 m. In contrast, Johnson *et al.* (2013) were not able to locate roots of large canopy lianas *Trichostigma octandrum* (Phytolaccaceae) and *Prionostemma aspera* (Celastraceae) at depths below 60 cm. More field data on depth and lateral extent of root systems, especially in canopy lianas, are needed.

Root systems must not only ensure adequate access to soil resources but must also provide mechanical anchorage. Clearly, the latter requirement is reduced in climbers in comparison with self-supporting plants. Do lianas therefore have lower root mass ratios? In the whole-plant allocation studies discussed earlier, the root mass fraction in climbers was smaller or no different in comparison with non-climbing species (Kaneko and Homma, 2006; Cai *et al.*, 2007). Supported *Wisteria floribunda* plants had a lower relative allocation to roots, but were also larger than unsupported plants (Sakai and Suzuki, 1999). Variability reported from pot studies of juvenile climbers (where root mass fraction in various species was between 9 and 71%; Teramura *et al.*, 1991) reflects a diversity of both phylogenetic backgrounds and ecological strategies of those plants. In spite of considerable progress in recent years, root systems continue to constitute the weakest link in our understanding of resource allocation patterns in wild plants, including lianas. This is particularly true for bigger plants, in which the relatively largest differences between climbing and self-supporting forms are expected.

## GROWTH RATES

Field data, especially the studies of invasive lianas, emphasize their fast growth (Schnitzer, 2005; Osunkoya *et al.*, 2010; Paul and Yavitt, 2011). Observations and measurements show that liana shoots elongate at a faster rate than those of non-climbing plants (Ruiz Fernandez, 1987; Teramura *et al.*, 1991; Schnitzer, 2005). This fast expansion rate is achieved because the construction of a shoot length unit of lianas requires a small amount of biomass, i.e. the specific shoot length is large (During *et al.*, 1994). The very fast shoot growth frequently seen in lianas under natural conditions (Teramura *et al.*, 1991) may, however, be also partly attributed to their ability to place apical meristems and leaves quickly in favourable (i.e. well-lit) locations and overgrow and shade their competitors, rather than to their inherently faster relative growth rate (Gartner, 1991). Availability of support seems to enhance growth at least in some species (Sakai and Suzuki, 1999), but see Frey and Frick (1987), Gartner (1991) and Tsugawa *et al.* (1992) for results showing no or little growth stimulation.

Relative growth rate, as well as its allocation components, are strongly dependent on plant size (e.g. Poorter and Pothmann, 1991; Poorter and Sack, 2012) and life stage. For example, many liana saplings may initially exhibit little shoot extension growth while developing an extensive root system that later allows them to take advantage of deep water sources while rapidly extending their shoots (S. A. Schnitzer, unpubl. obs.). Leaf area expansion was slower in liana seedlings than in tree seedlings in understorey sites in Panama (Brenes-Arguedas *et al.*, 2013). In contrast, seedling relative growth rate was greater in temperate lianas than in shrubs or trees (Cornelissen *et al.*, 1996), and light-demanding lianas showed a slightly faster growth rate than tree species, attributable to their thinner leaves and greater leaf area (Cai *et al.*, 2007). To date, no comparative growth analysis in lianas and erect species has been published in which size effects would be accounted for. Information on the relative growth rate of large individuals is lacking althogether.

## LEAF CHARACTERISTICS

Leaf-level traits are an important aspect of plant ecological strategies (Grime, 1977; Wright *et al.*, 2005*a*, *b*) because they influence a plant's CO_2_ uptake, transpiration, nutrient use efficiency and competitive ability, as well as susceptibility to various biotic and abiotic stressors. In a global view, liana leaves are considered to represent a ‘fast turnover/quick return’ end of the leaf trait spectra (Wright *et al.*, 2004*b*; Zhu and Cao, 2010). Traits associated with this type of leaf economics include low leaf mass per area (LMA; a measure of carbon construction costs of leaf area unit), low carbon cost of constructing a biomass unit, low investment in carbon-intensive defences, high photosynthetic and respiratory rates, high stomatal conductance, high nutrient [especially nitrogen (N)] concentration, fast leaf turnover or short life spans (Reich, 1998). Such trait combinations should ensure a ‘quick return’ on resource investment, i.e. fast plant growth and superior competitive ability. Here, we evaluate the existence of such a trait pattern in liana leaves using published multispecies comparative studies as well as our own database compiled from 436 published single- and multispecies reports and five unpublished data sets (see Supplementary Data Bibliography). Database entries were limited to woody angiosperms and were classified as shrubs, trees and climbers. Since data for some species were found in more than one source, we calculated and used the average values for the 3534 species with some species represented by more than one growth form (Supplementary Data Table S1).

### Photosynthesis

Lianas are generally believed to have higher average area-based gas exchange capacity, i.e. stomatal conductance and CO_2_ uptake capacity, than early successional and canopy trees with which they often compete (Castellanos, 1991; Teramura *et al.*, 1991; Zhu and Cao, 2009, 2010). In a set of tropical forest species, lianas had a faster gas exchange than trees only during the dry season (Cai *et al.*, 2009). These observations were not corroborated by our global database in which photosynthesis in lianas and trees did not differ but was lower in lianas than in shrubs (Table 1) or the meta-analysis of Wright *et al.* (2005*a*) in which area-based photosynthesis in climbers was lower than that in trees or shrubs. Two *in situ* multispecies studies conducted in a tropical forest in China (Han *et al.*, 2010) or a rainforest in Panama (Santiago and Wright, 2007), however, reported a lower area-based gas exchange capacity in lianas than in canopy trees, consistent with the global trend revealed by our database. The Santiago and Wright (2007) finding could be explained by the greater hydraulic limitation in lianas in which the narrow stems, even if highly conductive on a cross-sectional area basis, had to support a disproportionally large leaf area. Thus, whole-plant gas exchange may have been higher in lianas than in trees even though they had a lower photosynthetic rate per area.

**Table 1.** Leaf characteristics of wild shrubs (S), trees (T) and lianas (L) (trait mean, standard deviation, minimal and maximal values) based on a global survey of published literature reports

Variable	Growth form	*n*	Mean	s.d.	Min	Max	ANOVA *P*-value
LMA (g m^−2^)	L	132	**67·53**^**c**^	48·62	11·20	478·60	***
	T	1230	**127·82**^**b**^	168·25	23·90	3448·30	
	S	803	**173·97**^**a**^	172·52	22·90	1513·60	
A_max(area)_ (μmol m^−2^ s^−1^)	L	25	**9·05**^**b**^	3·58	0·61	16·90	**
	T	537	**9·90**^**b**^	4·40	1·45	26·31	
	S	379	**11·50**^**a**^	5·76	0·89	30·28	
A_max(mass)_ (nmol g^−1^ s^−1^)	L	20	138·59^a^	88·05	38·90	399·50	NS
	T	478	124·19^a^	94·06	8·70	933·50	
	S	330	118·39^a^	76·56	8·80	426·70	
N (mg g^−1^)	L	115	**23·91**^**c**^	9·55	10·65	53·50	***
	T	1820	**18·77**^**b**^	7·15	4·00	59·60	
	S	1042	**17·46**^**a**^	8·83	2·50	59·90	
P (mg g^−1^)	L	56	**1·55**^**c**^	1·20	0·18	6·09	***
	T	1266	**1·15**^**b**^	0·734	0·05	10·8	
	S	858	**1·22**^**a**^	0·967	0·09	9·70	
K (mg g^−1^)	L	41	13·47^a^	10·41	1·47	50·10	NS
	T	683	9·58^a^	5·56	0·60	44·50	
	S	327	10·87^a^	8·29	1·40	63·60	

To ensure morphological and taxonomic homogeneity of the samples, sampling was restricted to angiosperms. Families Arecaceae, Pandanaceae, Cyperaceae and Poaceae were excluded.

*P*-values for one-way analyses of variance for each trait are given in the last column (***P < 0·001; ** *P* < 0·01; * *P* < 0·05; NS, not significant). Where life form effect was significant mean trait values are given in bold font. For each trait, means marked by the same letter are not significantly different from one another as determined by HSD Tukey test (*P* < 0·05).

Photosyntehtic rate per unit of biomass is more directly related to leaf resource economics than is photosynthetic rate per area (Wright *et al.*, 2004*b*). Among *in situ* studies, mass-based photosynthesis was higher in lianas than in their supporting trees at Chinese tropical forest sites (Cai *et al.*, 2009; Zhu and Cao, 2010) but not in a Panamanian forest (Santiago and Wright, 2007) or in Chinese (Han *et al.*, 2010) forests. Van der Sande *et al.* (2013) also reported no difference in mass-based photosynthesis for small tree and liana saplings in Panama. Our database suggests that average mass-based photosynthesis is only insignificantly higher in lianas than in trees or shrubs (Table 1). The contrasting findings of the few studies cited above may be driven by species selection or the use of liana saplings that had not yet expressed adult traits. Overall, in spite of variability in outcomes of local studies, liana leaves photosynthesize at a slower rate than other woody plants when expressed on a leaf area basis but their leaf biomass shows a slight tendency for greater photosynthetic capacity.

### Respiration rate

In the global spectrum of plant economic traits, foliar respiration features as a strong correlate of leaf metabolic activity (Reich *et al.*, 2008). Leaf respiratory rates frequently differ among plant functional types (Reich *et al.*, 1998). Two studies have compared this important aspect of leaf economics in lianas and other growth forms. In a forest canopy studied in Costa Rica, lianas had higher per area respiratory rates than co-occurring trees, palms and herbs (Cavaleri *et al.*, 2008). Compared with other plant types, liana respiration also increased more rapidly with height position in the canopy, probably reflecting their stronger response of leaf mass per area and area-based nutrient concentration to light availability. Whereas Cavaleri *et al.* (2008) found no differentiation between functional types with respect to mass-based respiration, a study by Slot *et al.* (2013) conducted in an upper forest canopy in Panama showed a greater mass-based dark foliar respiration in lianas than in trees (and no pattern in area-based respiration). These contrasting findings do not provide support for consistent differences in respiration between lianas and trees; however, together they have only included 19 liana species, emphasizing the need for more *in situ* data. Both studies reported a slightly lower Q_10_ in lianas than in trees, suggesting lower maintenance costs of liana leaves at high temperatures.

### Leaf structure

Maximal photosynthetic and respiratory rates achieved by the leaf are influenced by its structural characteristics. An integrated measure of leaf structure, the LMA (leaf mass per area) denoting the amount of biomass invested in constructing a leaf area unit, is both widely measured and meaningful as it is correlated with several important functional traits such as photosynthetic capacity and leaf longevity (Wright *et al.*, 2004*a*). Do structural characteristics of leaves of lianas differ in a systematic way from those of self-supporting plants? Individual reports where LMA was compared between plant types (typically canopy trees vs. lianas) within a site yield a rather consistent picture. Three tropical forest studies from South China reported the LMA of lianas to be on average lower than that of trees, whether both forms were evergreen (Cai *et al.*, 2009) or included deciduous species (Zhu and Cao, 2010; Han *et al.*, 2010). Likewise, the LMA of lianas was significantly lower than that of trees in a lowland tropical rain forest in Gabon (Kazda and Salzer, 2000), an Ecuadorian montane forest (Salzer *et al.*, 2006) and in tropical dry forest and rain forest in Panama (Santiago and Wright, 2007; Sánchez-Azofeifa *et al.*, 2009). We found only two *in situ* studies where results deviated from this trend. Castellanos *et al.* (1989) found in a tropical deciduous forest in Mexico that lianas had a similar LMA to co-occurring trees and shrubs. In a tropical montane forest in China, Cai and Bongers (2007) found a slightly, but not significantly lower LMA in lianas compared with trees. According to our global literature database, lianas have a nearly 2-fold lower average LMA than trees (Table 1).

Our findings are consistent with a meta-analysis by Wright *et al.* (2005*a*) and a comprehensive data set from humid tropical forest sites across Neotropics, Madagascar and Australasia (Asner and Martin, 2012). Asner and Martin (2012) compared 563 liana species and 3322 tree species and reported a 16 % smaller LMA in lianas. Overall, the smaller mean LMA of climbers in comparison with trees seems to be well supported in global and, in most cases, in local comparisons.

Comparisons between lianas and shrubs are seldom drawn even though in many vegetation types (e.g. forest edges, gaps, recently disturbed sites) they co-exist and compete with each other. On the other hand, climbers are nearly absent from many arid, open or otherwise unproductive areas that are occupied by shrubs with sometimes extremely high LMA values. Our database showed that the mean LMA of lianas is nearly 3-fold lower than that of shrubs (Table 1), probably reflecting these different habitat spectra.

**Fig. 2.**
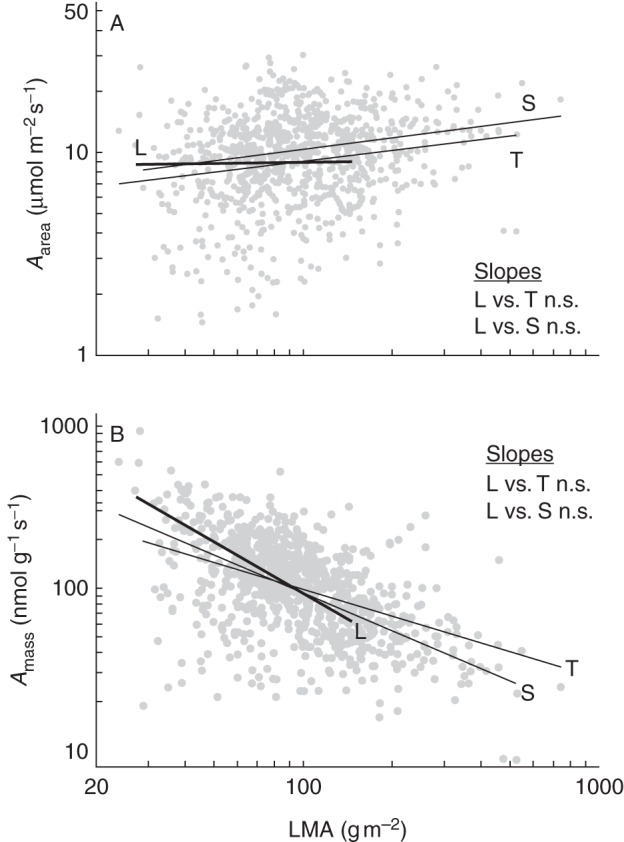
Regression relationships between leaf mass per area (LMA) and (A) maximal photosynthetic rate per leaf area (A_area_) and (B) maximal photosynthetic rate per leaf mass (A_mass_) in lianas (L), shrubs (S) and trees (T) based on literature data (see Supplementary Data Bibliography and Table S1). Regression lines run through the entire range of LMA for each growth form. Slope comparisons based on analysis of covariance between vines and the other growth forms are shown (n.s., not significant). Slopes and correlation coefficients for regression equations and associated probabilities are given in Table 2.

The slopes of relationships between LMA and photosynthetic capacity were non-significant in any growth form for area-based photosynthesis (Fig. 2A), whereas slopes were negative, highly significant and statistically indistinguishable from one another when photosynthesis was expressed on a leaf mass basis (Fig. 2B). These findings indicate that the slightly higher mass-based photosynthesis in lianas was attributable to their clustering closer to the lower end of the LMA continuum while subject to the economic rules common to all three growth forms.

**Table 2.** Slopes of linear regression of the log10-transformed area- and mass-based photosynthetic rate and nutrient concentrations against the log10-transformed LMA in lianas, trees and shrubs based on literature data sources

Trait	Lianas	Trees	Shrubs
logA_area_ (μmol m^−2^ s^−1^)	0·024 (*n* = 22, *r*^2^ = 0·000NS)	0·171 (*n* = 489, *r*^2^ = 0·03NS)	0·188 (*n* = 333, *r*^2^ = 0·05NS)
logA_mass_ (nmol g^−1^ s^−1^)	–1·050 (*n* = 20, *r*^2^ = 0·501***)	–0·774 (*n* = 477, *r*^2^ = 0·347***)	–0·548 (*n* = 330, *r*^2^ = 0·230***)
logN (mg g^−1^)	–0·488 (*n* = 95, *r*^2^ = 0·450***)	–0·501 (*n* = 1041, *r*^2^ = 0·398***)	–0·640 (*n* = 673, *r*^2^ = 0·593***)
logP (mg g^−1^)	–0·892 (*n* = 35, *r*^2^ = 0·324***)	–0·679 (*n* = 696, *r*^2^ = 0·341 ***)	–0·882 (*n* = 495, *r*^2^ = 0·519***)
logK (mg g^−1^)	–1·137 (*n* = 28, *r*^2^ = 0·241**)	–0·402 (*n* = 328, *r*^2^ = 0·141***)	–0·385 (*n* = 177, *r*^2^ = 0·121***)

The number of species used (*n*), correlation coefficients (*r*^2^) and significance levels (*P < 0·05; **P < 0·01, ***P < 0·001; NS, not significant) are given in parentheses.

Several other leaf structural traits with postulated functional consequences have been occasionally reported as associated with the climbing habit and potentially influencing the photosynthesis, productivity, growth and competitive ability. Liana leaves were smaller than those of trees in a Chinese (Cai *et al.*, 2009; Han *et al.*, 2010), Madagascan (Kazda *et al.*, 2009) and Andean forest (Salzer *et al.*, 2006), but not in a Mexican forest (Castellanos *et al.*, 1989). Smaller leaf lamina size increases the convective cooling effect by providing lower boundry layer resistance, especially in exposed canopy locations (Givnish and Vermeij, 1976). On the other hand, a smaller lamina requires less investment in structural support (Niinemets *et al.*, 2007), although relevant analyses for climbers vs. other growth forms appear to be lacking. At the anatomical level, leaf thickness was smaller in lianas, but the volume fraction of air spaces did not differ between lianas and trees (Sánches-Azofeifa *et al.*, 2009). Finally, liana and tree leaves had similar estimated biomass construction costs (1·37 vs. 1·38 g glucose g^−1^, respectively), but because of a smaller LMA, liana leaves had a lower construction cost of area unit (Zhu and Cao, 2010).

### Foliar nutrients

Mass-based photosynthetic capacity of leaves is largely determined by the concentration of N, because the majority of leaf N is contained in photosynthesis-related proteins (Evans, 1989). A higher N concentration in liana leaves in comparison with leaves of canopy trees was found in the majority of tropical forest studies (Kazda and Salzer, 2000; Salzer *et al.*, 2006; Cai *et al.*, 2009; Zhu and Cao, 2010; Asner and Martin, 2011, 2012) but not in those by Castellanos *et al.* (1989), Santiago and Wright (2007) and Sánchez-Azofeifa *et al.* (2009). In a growth chamber study, seedlings of climbers had greater leaf N concentration than seedlings of self-supporting species (Cornellissen *et al.*, 1997). Our own global database indicated a significantly greater leaf N concentration in lianas, compared with shrubs and trees (Table 1; see also Wright *et al.*, 2005*a*). A similar trend has been demonstrated for phosphorus (P) (Salzer *et al.*, 2006; Zhu and Cao, 2010; Asner and Martin, 2011, 2012; Table 1), although occasionally it occurred only during the dry season (Cai *et al.*, 2009) or was not statistically significant (Sánchez-Azofeifa *et al.*, 2009).

**Fig. 3.**
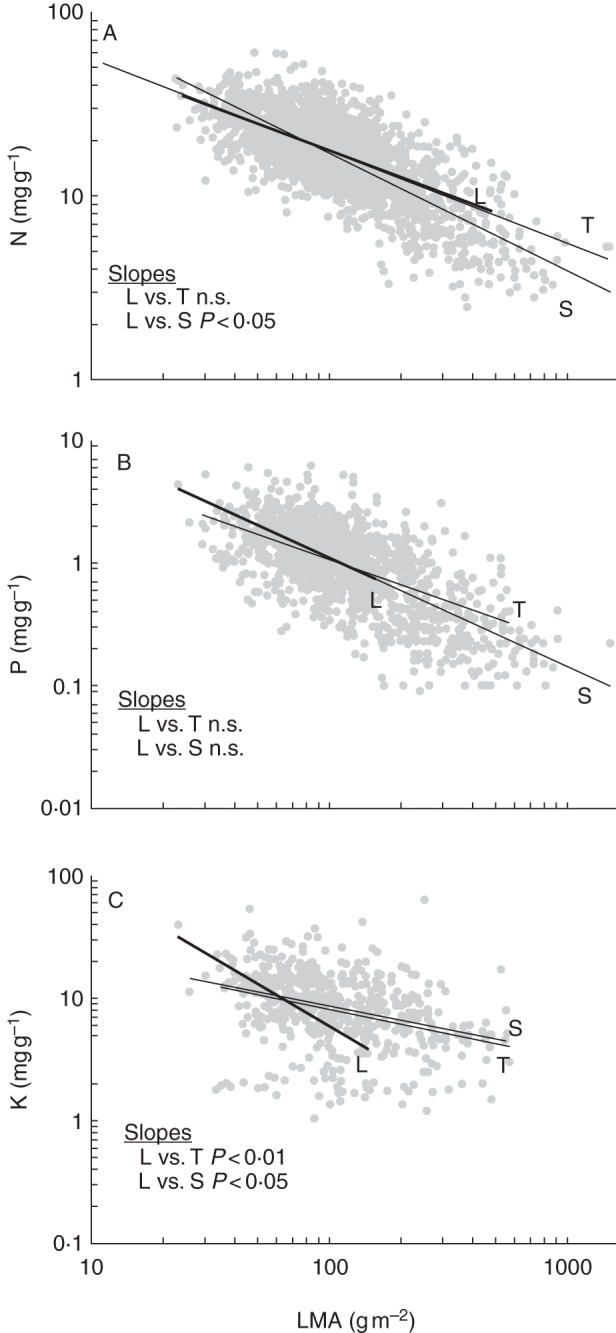
Regression relationships between mass-based concentrations of (A) nitrogen, (B) phosphorus and (C) potassium in lianas (L), shrubs (S) and trees (T) based on literature data (see Supplementary Data Bibliography and Table S1). Slope comparisons based on analysis of covariance between vines and the other growth forms are shown (n.s., not significant). Slopes and correlation coefficients for regression equations and associated probabilities are given in Table 2.

Robust interspecific relationships have been discovered betweeen leaf structure and the concentrations of some essential nutrients, with higher LMA leaves containing lower nutrient levels (Wright *et al.*, 2005*a*). This rule appears to hold across growth forms, as indicated by the similar regression slopes for nutrient concentrations between lianas and trees for N and between lianas and both trees and shrubs for P (Fig. 3A, B; Table 2). The exception is the greater slope of the N vs. LMA line in shrubs that could be caused by the existence of some shrub species with very high LMA and a disproportionally low N level. The range of LMA in lianas does not extend to the highest values, consistent with the generally higher concentration of N and P in liana leaf biomass, as reported in many individual studies (see above). These data indicate that the differences in mean N and P concentrations between lianas and other woody plants seem to be explained primarily by different ranges of leaf structural characteristics found in each growth form.

In local comparisons with trees, liana leaves appear to contain higher concentrations of potassium (K), a nutrient involved in osmotic adjustment and stomatal movements (Cornellissen *et al.*, 1997; Salzer *et al.*, 2006; Asner and Martin, 2011, 2012); however, globally this pattern was not statistically supported (Table 1). Interestingly, the regression of K against LMA was steeper in lianas than in other growth forms, with the most delicate (low LMA) leaves containing disproportionally high K levels (Fig. 3C). This finding extends a similar relationship reported by Salzer *et al.* (2006) for an assortment of woody and herbaceous climbers and their supporting trees. Moreover, the strength of the correlation of K with LMA is higher in lianas than in other growth types, perhaps indicating a special place for this nutrient in leaf economy of this growth form. One possibility is that the high K in thin climber leaves is part of the drought survival mechanism that involves tight stomatal regulation and bulk osmotic adjustent, and is expressed to a smaller degree in the more scleromorphic leaves of trees (Salzer *et al.*, 2006). Given the global correlation between N and P and only weak associations of K with N and P (Wright *et al.*, 2005*a*), it appears that at least some lianas are more specialized than trees and shrubs in maintaining high concentrations of all three nutrients. Reports on other nutrients are scarce. Asner and Martin (2012) reported greater concentrations of calcium, magnesium, zinc, manganese, boron and iron in lianas compared with trees. Salzer *et al.* (2006) found a smaller concentration of calcium but not magnesium or manganese in lianas in comparison with trees. The high concentrations of key nutrients in lianas together with their low LMA thus confirm the ‘fast turnover/quick return’ tendency of this group compared with other woody plants.

### Leaf hydraulics

Leaves are major resistance points for water transport (e.g. up to 90 % of all shoot resistance; Tyree and Zimmerman, 2002), and leaf hydraulic properties are an important determinant of gas exchange capacity (Sack and Holbrook, 2006; Brodribb *et al.*, 2010). The consensus view of a typical liana stem is that it possesses a highly efficient water transport system but has a high risk of conductivity loss because of the presence of wide and long conduits (Ewers *et al.*, 1990; Isnard and Silk, 2009); however, much remains to be learned about the hydraulic design of liana leaves. It is not clear whether the evolution of the climbing habit was also associated with an increased hydraulic conductance of leaves, e.g. through an increased diameter of xylem conduits or pits, reflecting modifications in the stems. Between two liana species found in the canopy of *Anacardium excelsum* (Anacardiaceae) trees in Panama, *Trichostigma octandrum* had higher stomatal conductance and leaf hydraulic conductance than *Prionostemma aspera* and their host trees (Johnson *et al.*, 2013). Remarkably, leaves of both lianas reached lower mid-day water potentials and were more vulnerable to embolism than their host trees, although no details on leaf xylem structure were provided. Susceptibility of liana leaves to hydraulic disruption might prevent embolization of the stem further upstream by reducing transpiration-induced xylem tension (Johnson *et al.*, 2013). As an alternative mechanism, large leaves, such as those of *Aristolochia macrophylla* (Aristolochiaceae), may exhibit spatial variability in hydraulic conductance and stomatal closure, consistent with the need to prevent vein embolization of the laminas (Miranda *et al.*, 2013). Other than the need for prevention of stem embolism, leaf hydraulic conductance may not need to be higher in lianas than in self-supporting species because of the larger leaf area supplied by each unit of stem sapwood in lianas (Putz, 1983; Ewers *et al.*, 1991). Also, given that leaf conductance tends to be coupled to gas exchange potential per leaf area (Brodribb *et al.*, 2010), the hydraulic design of liana leaves is not expected to be on average more efficient than in self-supporting plants; however, in local comparisons of co-occurring plants, the occasional higher gas exchange rate of lianas (Castellanos, 1991; Teramura *et al.*, 1991; Zhu and Cao, 2009, 2010; Johnson *et al.*, 2013) may be linked to their higher leaf hydraulic efficiency.

### Leaf life span

Leaf life span is increasingly recognized for its influence on plant canopy functioning. In some studies, lianas tend to have shorter leaf life spans and lower LMA than trees (Castellanos, 1991; Zhu and Cao, 2010), consistent with the widely acknowledged global correlation (Wright *et al.*, 2004*b*, 2005*a*). Consequently, in tropical and sub-tropical forests, leaf litter production from lianas is much greater than would be expected from their biomass contribution (Yong *et al.*, 2012). The extensive compilation of leaf longevities used in the review paper by Wright *et al.* (2005*a*) included very few climbers; however, it suggested a similar mean longevity for lianas and erect woody species. Similarly, a survey of leaf longevities in tropical understorey species in Indonesia showed both large variability and lack of consistent differences between growth forms in spite of a lower LMA in climbers (Shiodera *et al.*, 2008). The latter authors suggest that the uncoupling of LMA and longevity relationship in lianas can be attributed to a lower requirement for leaf lamina support in semi-epiphytic climbers. Locally lianas may even have a longer life span than canopy trees, at least in situations where the former are less hydraulically stressed (Cai *et al.*, 2009).

### Foliar defences

**Fig. 4.**
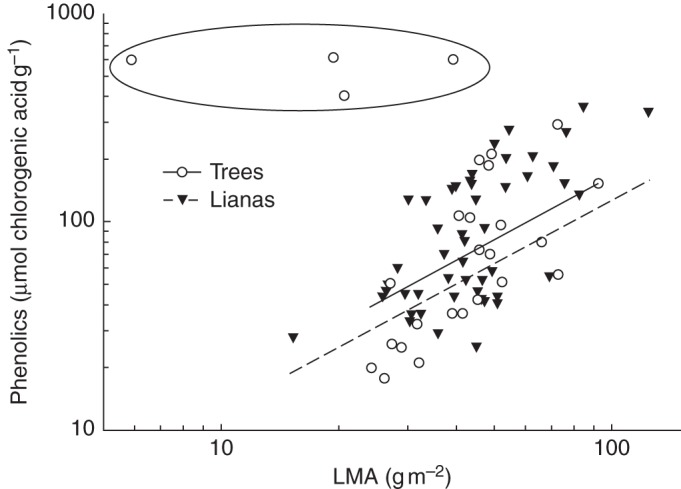
Relationships between leaf mass per area (LMA) and total phenolic concentration in leaves of ten lianas and seven tree species collected at a forest site in Panama (S. A. Schnitzer *et al.*, unpubl.). Each species is represented by 1–7 leaves from diverse light environments. Regression equations are: for lianas, logPhenolics = –0·150 + 1·264 × logLMA (*n* = 49, *r*^2^ = 0·40 *P* < 0·0001); and for trees, logPhenolics = –0·924 + 1·663 × logLMA (*n* = 23, *r*^2^ = 0·52 *P* < 0·0001). Slopes and intercepts were compared by analysis of covariance with, and subsequently without growth form × LMA interaction, and were found not to be statistically different (both *P* > 0·05). The four outlying points surrounded by an elipse belong to tree species *Quassia amara* and were excluded from the analysis. The method of phenolic compound determination has been described in Karolewski *et al.* (2010).

Analyses of resource economics supported by field data show that long-lived (high LMA) leaves require an increased investment in carbon-intensive, constitutive and stable chemical defences in contrast to short-lived (low LMA) leaves that preferentially rely on N-intensive, high-turnover, defensive compounds (Coley *et al.*, 1985; but see Agrawal, 2011 for a more nuanced view). Whereas secondary metabolites have been well characterized in woody plants, including climbers, from many different lineages, the relationship between type and amount of defensive compounds and the growth form has received little attention. In agreement with the expectations of a more opportunistic life strategy of lianas, concentrations of carbon-demanding compounds were lower in leaves of lianas than in trees at Amazon and Bornean forest sites (Asner and Martin, 2011; Asner *et al.*, 2012) as well as in a world-wide tropical study (lignins by 11 %, phenols by 25 % and tannins by 19 %; Asner and Martin, 2012). Our own unpublished data on total phenolic compounds in leaves of 11 liana and seven tree species collected from a seasonally semi-deciduous secondary forest on Gigante Peninsula (part of the Barro Colorado Nature Monument) in central Panama (for a site description, see Schnitzer and Carson, 2010) revealed neither a statistical difference between growth forms nor a relationship to LMA (Fig. 4). However, when *Quassia amara* (Simaroubaceae), a low LMA tree particularly rich in phenolics, was removed from the data set, LMA became a significant negative predictor of phenolic concentration even if trees and lianas still did not differ in either LMA or phenolics (Fig. 4). These findings suggest that other, more typical sample sets, with lianas having lower LMA than trees, may show lower phenolic concentrations in lianas. Nothwithstanding the scarcity of comparative studies, the cited examples indicate that climbing habit, leaf longevity and defences appear to be linked in ecologically meaningful ways.

## STEM CHARACTERISTICS AND MODIFICATIONS FOR CLIMBING

Stem characteristics of climbing plants are particularly distinct and have been described in various publications (e.g. Carlquist, 1991; Isnard and Silk, 2009; Angyalossy *et al.*, 2012, 2013). A variety of climbing mechanisms have been described that are based on morphological modifications of above-ground organs. Apart from species equipped with such well known devices as tendrils (which can arise from leaf, flower or branch modifications), adhesive pads, twining stems, spines and thorns, adventitious roots and lignified petiole bases (Isnard and Silk, 2009), there exist specialized branch-angle climbers with stems originally stiff and assuming a flexible structure only after finding a support (Ménard *et al.*, 2009).

The understanding of liana stem structure comes primarily from interspecific comparisons, but also from studying ontogenetic and plastic modifications within lianas themselves. Many species undergo a juvenile self-supporting phase when the plant habit and stem stiffness are indistinguishable from those of shrubs or even trees of similar stature (Rowe and Speck, 2004; Gallenmüller *et al.*, 2004). There are also species, such as *Machaerium floribundum* (Fabaceae), that may exist either as lianas or as fully grown trees up to 25 m high (Angyalossy *et al.*, 2012). Studies of plastic changes within species accompanying switches between alternative growth forms are especially valuable as they separate functional trait associations from those resulting from the phylogenetic background. Insights into stem biomechanical design can be gained from studies of specialized, rather stiff searcher shoots, on which leaf development is delayed, that are formed by many liana species, enabling an attachment to a primary support as well as efficient movement of the stem tip across the forest canopy (Rowe and Speck, 2004). Once a searcher shoot reaches a support, this rigid stem undergoes a transformation of anatomical structure, assuming a typical flexible design (Hoffman *et al.*, 2003; Isnard and Silk, 2009; Ménard *et al.*, 2009). This transformation involves secondary growth and, e.g. in *Aristolochia macrophylla* and climbing species of *Clematis*, results in the loss of the outer layer of mechanical tissues (Isnard *et al.*, 2003; Masselter and Speck, 2008). Other rigidity-reducing internal modifications of climbing stems include discontinuous or supranumerary cambia that variously modify the transverse geometry of the stem [e.g. by producing wedges or additional rings of phloem (Carlquist, 1991; Isnard and Silk, 2009; Angyalossy *et al.*, 2012)]. Liana stem wood has a larger amount of axial and radial parenchyma than stems of trees (Carlquist 1991; Angyalossy *et al.*, 2012), enhancing their flexibility, but also their water, carbohydrate and nutrient storage capacity (Mooney and Gartner, 1991). Liana wood may also have a smaller content of fibres, e.g. in comparison with co-occurring sub-shrubs (29 % vs. 49 % of stem cross-sectional area; Crivellaro *et al.*, 2012) or between conspecific lianescent vs. arborescent forms (16 % vs. 63 % in *M. floribundum*; Angyalossy *et al.*, 2012) as well as an altered wood cell wall structure and chemical composition (Hoffmann *et al.*, 2003). Other specialized structural traits include gelatinous fibres enhancing the coiling ability of twining stems (Bowling and Vaughn, 2009). Lianas also display an extraordinary ability for healing of mechanically damaged stems (Fisher and Ewers, 1989).

Liana stems are narrow in relation to supported leaf area (Ewers and Fisher, 1991). Accordingly, their vessels tend to be among the longest and widest among the vascular plants, reflecting the necessity to move copious quantities of water quickly (Ewers *et al.* 1990, 1991, 1997*a*). While the benefits of the wide liana vessels are fairly well understood, potential trade-offs have also been documented. Although the long-held view that the high hydraulic efficiency of liana stems also makes them more vulnerable to drought-induced cavitation has been questioned (Ewers *et al.*, 1991), there is recent evidence that liana stems cavitate at less negative pressures than trees both as canopy-level plants (Zhu and Cao, 2009) and as young saplings (Van der Sande *et al.*, 2013). In a multispecies study, the widest and longest vessels were found in two climbers that also cavitated under the least negative pressure (Hacke *et al.*, 2006). Calculations of theoretical vulnerability to cavitation based on vessel diameter and vessel frequency indicated that vulnerability in lianas was more than twice larger than in trees at a Mexican rain forest site (Gutierrez *et al.*, 2009). High vulnerability to drought-induced cavitation in climbers may also be attributed to the large pit area fraction of vessel walls (Hacke *et al.*, 2006). At least in some families unique radial conducting cells originating from cambial ray initials connect neighbouring vessels, perhaps providing by-passes for water flow around embolisms (Angyalossy *et al.*, 2012). Wide vessels also make lianas vulnerable to cavitation induced by freeze–thaw events, possibly accounting for the poor representation of these life forms at higher latitudes (Jiménez-Castillo and Lusk, 2013). The hydraulic design of xylem thus appears fundamental for the ecological performance of lianas. Remarkably, there exists a liana *Tasmannia cordata* (Winteraceae) that is genetically unable to produce vessels and instead uses tracheids for water conduction, achieving much lower hydraulic conductivities than lianas with vessels. It appears, however, that the vessel-less strategy is an evolutionary dead end and allows the plant to persist only in a moist, low statured cloud forest of Papua New Guinea (Feild *et al.*, 2011).

## ROOT CHARACTERISTICS

Except for sporadic qualitative investigations into liana root anatomy (e.g. see the ongoing Anatomy of the Dicotyledons series initated by R. Metcalfe, Oxford University Press, UK) and measurements of root pressure (e.g. Fisher *et al.*, 1997), many structural aspects of liana root system biology are still poorly known. It is well established that stems of climbers have a highly conductive xylem containing large vessels with thin cell walls, implying a low safety factor and a vulnerability to disruption (Putz, 1983; Sperry *et al.*, 1987; Ewers and Fisher, 1991). However, it is, at present, too early to make parallel generalizations for their roots. Although systematic comparisons of root and stem hydraulic conductance in lianas are not available, a survey of many tropical liana and tree species showed that liana root vessels were narrower than in stems and similar with respect to diameter to root vessels of trees (Ewers *et al.*, 1997*a*). This pattern would suggest that, for climbing plants, individual root conduits provide relatively greater resistance to water flow than in stems. However, Tyree and Ewers (1996) reported that the total transverse root area of the liana species *Machaerium milleflorum* (Fabaceae) on Barro Colorado Island, Panama was nearly eight times larger than that of its stem. They found that a single *M. milleflorum* individual with a 20 cm stem diameter had four roots >20 cm diameter, including a 50 cm taproot that reached a depth of 4·4 m. This example indicates that integrated root hydraulic conductance may not constitute a bottleneck in water transport to leaves.

To avoid upstream embolism formation and restriction to water flow, liana roots have to maintain a continuous supply of water. Moreover, positive pressure exerted by the roots reduces upstream tensions and may help prevent embolism formation and, at least in grapevine, may also help eliminate embolisms (Sperry *et al.*, 1987). There is evidence that root pressure in some tropical (Fisher *et al.*, 1997) and temperate (Jiménez-Castillo and Lusk, 2013) lianas may generate a positive stem water pressure at least during the wet season and at night. However, these root pressures may not be positive enough to clear embolisms that are more than a few metres above the soil surface. Among 29 dicotyledonous liana species studied in Panama by Ewers *et al.* (1997*b*), as many as 26 species did not generate positive pre-dawn pressure, whereas three species representing the family Dilleniaceae generated root pressures that were too low to fill vessels higher than 6·4 m in 18 m stems. Only two small statured climbers, a fern (*Lygodium venustum*, Schizaeaceae) and a bamboo (*Rhipidocladum racemiflorum*, Poaceae), generated root pressures sufficient to push water to the apex of the plant (Tyree and Ewers, 1996; Ewers *et al.*, 1997*b*). In contrast, all five temperate lianas studied in Chile showed positive root pressure, the magnitude of which was correlated with vessel diameter, suggesting a link between species' vulnerability to freeze–thaw-induced embolism and ability to generate root pressure (Jiménez-Castillo and Lusk, 2013).

In some temperate liana species, water stored in roots may be involved in the maintenance of elevated xylem water potential even when the transpiration is high (Teramura *et al.*, 1991). Some climbers, especially those from open, periodically dry habitats, possess large lignotubers specialized for water but also carbohydrate storage and allowing regeneration of the herbaceous or woody stems after disturbance (Ewers *et al.*, 1991; Tyree and Ewers, 1996; Rundel and Franklin, 1991). Water storage in axial organs is usually attributed to elastic xylem parenchyma (in both axial and radial systems). Comparative allometric analyses (based on tissue volume fraction) of the anatomical composition of xylem and other tissues, such as those conducted for stems of *Bauhinia* (Ewers and Fisher, 1991), are needed to uncover the basis for root water storage in lianas.

The macromorphological structure of root systems in lianas, as in other plants, greatly influences the water absorption ability (Pregitzer, 2008). It appears, however, that formal architectural analysis has only been used sparsely to investigate root system attributes of lianas. A soil coring of two liana species showed that the majority (74 %) of fine root area was allocated to the upper 30 cm of soil profile (Johnson *et al.*, 2013). Based on information gathered for other growth forms, root architecture in lianas could represent a spectrum from shallow extensively branched systems absorbing water from the upper layer of the soil to deep-rooted species with extended taproots exploring deep horizons of the soil profile (Fitter, 2002; Hutchings and John, 2003). Seasonal water use characteristics (such as leaf stomatal conductance and leaf area ratio) may correlate with these root traits. At the same time, the utilization of other plants for support might mean that coarse, structural axes in the root system are redundant.

Further modifications of the liana root system may include adventitious roots formed along the stem as it touches the ground, e.g. following disturbance (Paul and Yavitt, 2011). Sub-terranean organs may also include woody rhizomes such as described for the genus *Stigmaphyllon* (Malpighiaceae; Anderson 1997). So far, in spite of sporadic publications (Tyree and Ewers, 1996; Andrade *et al.*, 2005; Johnson *et al.*, 2013), the relationship between root structure and climbing habit remains to be elucidated.

## SOURCES OF VARIABILITY

Woody climbers as a group form a phenotypic continuum from highly specialized obligate lianas to species with intermediate growth forms (scramblers) devoid of specialized climbing mechanisms but nevertheless using surrounding vegetation for support. Not surprisingly, when traits of lianas are evaluated on a global basis, they reveal an extensive variation magnified by the remarkable plasticity either in response to support availability or as part of intrinsic ontogenetic programmes. Lianas also include some spectacular examples of morpho-anatomical transformations between juvenile and mature shoot phases and of heteroblastic leaf differentiation (Lee and Richards, 1991).

Further variability may be attributed to interspecific differences in successional status, habitat soil resource richness and climbing mechanisms (Hegarty, 1990; Gerwing, 2004; Kusumoto *et al.*, 2012). Although tropical forests abound in various forms of lianas (Putz, 1984; Gallagher and Leishman, 2012; Kusumoto *et al.*, 2012; Schnitzer *et al.*, 2012), ecological niche differentiation among species with different mechanisms has been noted by several researchers (Putz, 1984; Kusumoto *et al.*, 2012). For example, adventitious root climbers frequently occupy inner, shaded regions of the tropical forest canopy (Kusumoto *et al.*, 2012) and, among temperate liana species, have the lowest photosynthetic rates (Carter and Teramura, 1988). Tropical tendrilled lianas retain leaves for a shorter time than other lianas from the site (Hegarty, 1990) and show preference for sites with an abundance of thin supporting stems, i.e. they tend to be early succession specialists (DeWalt *et al.*, 2000). No relationship, however, was found between climbing mechanism and latitude in a global sample of 1092 climbing species (Gallagher and Leishman, 2012). Seeking the link between climbing mechanisms and other functional traits appears to be a fruitful area for future exploration.

Average differences between climbers and other growth forms, whether addressed on local or on regional scales, will be affected by site climatic conditions favouring high or low climber diversity (Asner and Martin, 2012; Gallagher and Leishman, 2012). Further, although climbers have evolved in a vast number of plant lineages, there are families in which lianas are either exclusive or dominant growth forms (e.g. Celastraceae, Passifloraceae and Vitaceae). It is possible that a large local representation of these specialized families or other speciose liana-rich taxa, such as tribe Bignonieae in the Bignoniaceae, would introduce a phylogenetic signal unbalanced by closely related self-supporting plants. Studies that both retain a biogeographic perspective and control for the phylogenetic trait conservatism are the most useful in elucidating the consequences of evolution of the climbing habit (Asner *et al.*, 2012; Gallagher and Leishman, 2012).

In summary, there is empirical support for global covariation of selected traits, such as increased leaf/stem mass ratios (at larger body sizes), delicate, nutrient-rich leaves and fast stem extension growth with the climbing habit. These traits thus contribute to what may be called ‘the climbing plant syndrome’ (Table 3). This review has also identified gaps in the knowledge of functional traits of climbers (Table 4), such as patterns of resource allocation to support, growth rate, hydraulics and anatomy of leaves and roots that will need to be answered by future comparative and meta-analytical studies.

**Table 3.** Functional traits of lianas as compared with trees based on a global review of published data and observations

Trait category	Lianas vs. trees	Key references
Plant biomass allocation	Greater ratio of leaf mass to above-ground biomass (at large plant biomass)	Putz (1983); Gerwing and Farias (2000); Chave *et al.* (2001); Schnitzer *et al.* (2006); this review
	Greater ratios of leaf mass and leaf area to stem cross-sectional area	Putz (1983); Gerwing and Farias (2000)
Growth rate	Faster relative growth rate	Cornelissen *et al*. (1996); Cai *et al.* (2007)
	Faster absolute shoot extension rate	Ruiz Fernandez (1987); Teramura *et al.* (1991); Schnitzer (2005); Gillian and Yavitt (2011)
	Slower stem diameter growth	Putz (1990)
Photosynthetic rate	Comparable on a leaf area basis	Castellanos (1991); Teramura *et al.* (1991); Wright *et al.* (2005*a*); Santiago and Wright (2007); Cai *et al.* (2009); Zhu and Cao (2009, 2010); Han *et al.* (2010); Van der Sande *et al.* (2013); this review
	Slightly (non-significantly) higher on a leaf mass basis	Santiago and Wright (2007); Cai *et al.* (2009; Zhu and Cao (2010); Han *et al.* (2010)
Leaf respiratory rate	No consistent difference at 25 °C (on a mass basis)	Cavaleri *et al.* (2008); Slot *et al.* (2013)
	Slightly lower Q_10_	Cavaleri *et al.* (2008); Slot *et al.* (2013)
Leaf structural traits	Smaller lamina area	Castellanos *et al.* (1989); Salzer *et al.* (2006); Cai *et al.* (2009); Han *et al.* (2010)
	Lower LMA (leaf mass per area)	Castellanos *et al.* (1989); Kazda and Salzer (2000); Wright *et al.* (2005*a*); Salzer *et al.* (2006); Cai and Bongers (2007); Santiago and Wright (2007); Cai *et al.* (2009); Sánchez-Azofeifa *et al.* (2009); Zhu and Cao (2010); Han *et al.* (2010); Asner and Martin (2012); this review
	Smaller leaf thickness	Sánchez-Azofeifa *et al.* (2009)
	Similar volume fraction of air spaces	Sánchez-Azofeifa *et al.* (2009)
Foliar nutrients	Higher N per leaf mass	Castellanos *et al.* (1989); Cornelissen *et al.* (1996); Kazda and Salzer (2000); Wright *et al.* (2005*a*); Salzer *et al.* (2006); Santiago and Wright (2007); Cai *et al.* (2009); Sánchez-Azofeifa *et al.* (2009); Zhu and Cao (2010); Asner and Martin (2011, 2012); this review
	Higher P per leaf mass	Salzer *et al.* (2006); Cai *et al.* (2009); Sánchez-Azofeifa *et al.* (2009); Zhu and Cao (2010); Asner and Martin (2011, 2012); this review
	Some species with much greater K per leaf mass	Cornellissen *et al.* (1997); Salzer *et al.* (2006); Asner and Martin (2011, 2012); this review
	Higher Ca, Mg, Zn, Mn, B and Fe per leaf mass	Salzer *et al.* (2006); Asner and Martin (2012)
Carbon-based foliar defences	Lower concentration of phenolics	Asner and Martin (2011, 2012); Asner *et al.* (2012); this review
Stem traits	Developmental transitions of stem habit (self-supporting vs. climbing vs. searching)	Hoffman *et al.* (2003); Rowe and Speck (2004); Gallenmüller *et al.* (2004); Ménard *et al.* (2009); Isnard and Silk (2009); Angyalossy *et al.* (2012)
	Occurrence of anomalous vascular cambia and modified secondary growth pattern	Schenck (1893); Carlquist (1991); Caballé (1993); Isnard and Silk (2009); Angyalossy *et al.* (2012)
	Longer and wider vessels and higher hydraulic conductivity	Ewers (1985); Ewers *et al.* (1990, 1991, 1997*a*); Gartner *et al.* (1990); Carlquist (1991)
	Higher susceptibility to cavitation	Hacke *et al.* (2006); Gutiérrez *et al.* (2009); Zhu and Cao (2009); Feild *et al.* (2011); Van der Sande *et al.* (2013); Jiménez-Castillo and Lusk (2013)
	Lower wood density	Putz (1983); Gallenmüller *et al.* (2004); Zhu and Cao (2009)
	Lower wood and stem stiffness	Speck and Rowe (1999); Hoffmann *et al.* (2003); Gallenmüller *et al.* (2004); Ménard *et al.* (2009);
	Higher stem healing ability	Fisher and Ewers (1989, 1991)
Root system traits	Greater rooting depth	Restom and Nepstad (2004); Andrade *et al.* (2005)
	Frequent positive root pressure	Tyree and Ewers (1996); Fisher *et al.* (1997); Ewers *et al.* (1997*b*); Jiménez-Castillo and Lusk (2013)
	Similar vessel diameter	Ewers *et al.* (1997*a*)
	Variety of modifications (adventitious rooting, lignotubers)	Ewers *et al.* (1991); Tyree and Ewers (1996); Rundel and Franklin (1991)

Traits directly associated with the climbing mechanisms have been ommitted. References cited here provide both supporting and contrary data.

**Table 4.** Key questions on structure–function relationships in lianas that need to be addressed by future research

No.	Question
1.	What are the ratios of biomass partitioning to particular organ types in fully grown lianas?
2.	Are relative growth rates higher in lianas than in self-supporting plants of the same size?
3.	How do key leaf traits contribute to whole-plant carbon gain in lianas vs. self-supporting plants?
4.	To what extent do leaf hydraulic traits mirror those of the stems?
5.	What are the depths and morphological characteristics of the liana root systems?
6.	What mechanisms are responsible for triggering the switch between self-supporting and lianescent growth habit in species exhibiting developmental plasticity?
7.	Do lianas with different climbing mechanisms form separate groups with respect to other functional traits?

## SUPPLEMENTARY DATA

Supplementary data are available online at www.aob.oxfordjournals.org and consist of the following. Bibliography: sources used to produce Figs 2 and 3. Table S1: list of plant taxa used in analysis of leaf traits in Figs 2 and 3.

Supplementary Data
