# Supplementary material for: Phenotypic correlates of the lianescent growth form: a review
Source: Ann Bot. 2013 Oct 29;112(9):1667–81. doi: 10.1093/aob/mct236 (PMC3838560; doi:10.1093/aob/mct236)
Supplement: Supplementary Data [file supp_112_9_1667__index.html]

Phenotypic correlates of the lianescent growth form: a review — Supplementary Data 

# Phenotypic correlates of the lianescent growth form: a review

## Supplementary Data

Supplementary Data

**Files in this Data Supplement:**

- Supplementary Data - Pdf file
- Supplementary Table - pdf file
