# Supplementary material for: Phenotypic correlates of the lianescent growth form: a review
Source: Ann Bot. 2013 Oct 29;112(9):1667–81. doi: 10.1093/aob/mct236 (PMC3838560; doi:10.1093/aob/mct236)
Supplement: Supplementary Data [file supp_mct236_mct236supp.pdf]

SUPPLEMENTARY DATA

Bibliography: literature data sources used to produce Figs 2 and 3.

Abrams, M.D., Mostoller, S.A. 1995. *Tree Physiol*. **15**: 361-370.

Ackerly, D.D., Knight, C.A., Weiss, S.B., et al. 2002. *Oecologia* **130**: 449-457.

Adams, M.B., Burger, J., Zelazny L., et al. 2004. Description of the Fork Mountain long-term soil productivity study: site characterization. USDA General Technical Report NE-323, 40 p.

Adams, M.B., Kochenderfer, J.N., Angradi, T.R., et al. 1995. Nutrient budgets of two watersheds of the Fernow Experimental Forest. In: Gottschlak, K.W., Fosbroke, S.L.C. (eds.) Proceedings 10th Central Hardwood Forest Conference, Morgentown, West Virginia, March 5-8, 1995. US Department of Agriculture, Forest Service, Northeastern Forest Experiment Station, General Technical Report NC-197, pp. 119-130.

Alban, D.H., Perela, D.A., Schlaegel, B.E. 1978. *Can. J. For. Res*. **8**: 290-299.

Allen, S.E. 1989. Chemical analysis of ecological materials, 2nd edn. Blackwell Scientific, Oxford.

Alonso, C., Herrera, C.M. 2001. *New Phytol.* **150**: 629–640.

Alriksson, A., Eriksson, H.M. 1998. *Forest Ecol. Manage*. **108**: 261-273.

Amores, G., Santamaria, J.M. 2003. Beech foliar chemical composition: A bioindicator of air pollution stress. In: Air pollution, Global Change and Forests in the New Millennium. D.F. Karnosky et al. (eds.). Elsevier LTD., pp. 301-313.

Anderson, J.E., Kriedemann, P.E., Austin, M.P., et al. 2000. *Austr. J. Bot*. **48**: 759-775.

Anonymous 1997. A13 national Report: Slovakia. In: Forest Foliar Condition in Europe. UN Economic Commission for Europe, European Commission. EC-UN/ECE-FBVA, Brussels, Geneva, Vienna (ISBN 3-901347-05-4), pp. 116-119

Aranda, I., Pardo, F., Gil, L., et al. 2004. *Acta Oecol.* **25**: 187-195.

Arutiunian, A.S., Santurian, V.S. 1964. *Pochvovedenie* **3**: 24-29.

Auclair AND, Rencz AN. 1982. *Can. J. For. Res.* **12**: 947-968.

Balsberg Pahlsson A-M. 1992. *Tree Physiol.* **10**: 93-100.

Balsberg Pahlsson, A.-M. 1989. *Tree Physiol.* **5**: 485-495.

Bard, G.E. 1949. *Ecology* **30**: 384-389.

Bardi, M.A., Pulford, I.D., Springuel, I. 1996. *J. Arid Environ*. **32**: 421-429.

Barrick, K.A. 2003. *Austr. Ecol*. **28**: 252-262.

Bartos, D.L., Johnson, R.S. 1978. *Forest Sci*. **24**: 273-280.

Baruch, Z., Goldstein, G. 1999. *Oecologia* **121**: 183-192.

Basset, Y. 1996. *Ecology* **77**: 1906-1919.

Bauer, G., Schulze, E.-D., Mund, M. 1997. *Tree Phys*. **17**: 777-786.

Bazilevich, N.I. 1955. *Pochvovedenie* **4**: 1-32.

Bazilevich, N.I., Semenyuk, N.V. 1986. *Pochvovedenie* **7**: 57-69.

Beaulieu, J.M., Leitch, I.J., Knight, C.A. 2008. *Ann. Bot*. **99**: 495-505.

Bedford, B.L., Walbridge, M.R., Aldous, A. 1999. *Ecology* **80**: 2151-2169.

Bellingham, P.J., Walker, L.R., Wardle, D.A. 2001. *J. Ecol*. **89**: 861–875.

Belyaev, A.B., Alexandrovich, V.E., Kalutsku, K.K. 1976. *Pochvovedenie* **2**: 95-106.

Berki, I. 1991. *AFZ* **2**: 74-78.

Bernier B, Brazeau M. 1988. *Can. J. For. Res.* **18**: 1865-1269.

Bernier B, Brazeau M. 1988. *Can. J. For. Res.* **18**: 754-761.

Bloom, R,G., Malik A.U. 2004. *Plant and Soil* **265**: 279-293.

Bloor, J.M.G., Grubb, P.J. 2004. *Funct. Ecol*. **18**: 337–348.

Bockheim JG, Leide JE. 1991. *Can. J. For. Res.* **21**: 925-934.

Bolyshev, N.N., Vinnik, M.A., Konnova, E.N. 1967. *Pochvovedenie* **6**: 93-104.

Bonal, D. Sabatier, P. Montpied, D., et al. 2000. *Oecologia* **124**: 454-468.

Bonal, D., Barigah, T.S., Granier, A., et al. 2000. *Plant Cell Environ*. **23**: 445-459.

Bongers, F., Pompa, J. 1988. Trees and gaps in a Mexican tropical rain forest: species differentiation in relation to gap-associated environmental heterogeneity. PhD Thesis (ISBN 90-9002344-5), 185 pp.

Bouat, A. 1987. Olives: introduction and general. In Martin-Prevel, P., Gagnard, J., Gautier, P. (Eds.) Plant Analysis as a guide to the nutrient requirements of temperate and tropical crops. Lavoisier Publishing Inc. New York., pp. 299-309

Bowersox, T.W., Ward, W.W. 1977. *Forest Sci*. **23**: 433-436.

Bowersox, T.W., Ward, W.W. 1977. *Forest Sci*. **23**: 463-469.

Brandtberg, P.-O., Bengtsson, J., Lundkvist, H. 2004. *For. Ecol. Manage*. **198**: 193-208.

Broadford, W.M., Farmer, R.E. Jr. 1969. *Forest Sci*. **15**: 46-48.

Brodribb, T.J., Holbrook, N.M. 2005. *Trees* **19**: 290-295.

Brown, K.R., van den Driessche, R. 2005. *New Forests* **29**: 89–104.

Brozek S. 1990. *Can. J. For. Res.* **20**: 1320-1325.

Bruun, H. H., Osterdahl, S., Moen, J. et al. 2005. *Ecography* **28**: 81-87.

Burke, M.K., Lockaby, B., Graeme, B. et al. 1999. *Can. J. For. Res*. **29**: 1402-1418.

Bussotti F. et al. 1992. *Forest Ecol. Manage.* **51**: 81-93.

Bussotti, E., Pancrazi, M., Matteucci, G., et al. 2005. *Tree Physiol*. **25**: 211-219.

Bussotti, F., Borghini, F., Celesti, C. et al. 2000. *Trees* **14**: 361-368.

Buwalda J.G., Smith, G.S. 1987. *Tree Physiol.* **3**: 295-307.

Caldeira, M.V.W., Schumacher, M.V., Spathelf, P. 2002. *Ann. For. Sci*. 59: 833-838.

Cao, K.-F. 2000. *Can. J. Bot*. **78**: 1245-1253.

Castell, C., Terradas, J. 1995. *Tree Physiol*. **15**: 405-409

Castellanos, A, Mooney, H.A., Bullock, S.H. et al. 1989. *Biotropica* **21**: 41-49.

Castro-Díez, P., Puyravaud J.P., Cornelissen J.H.C. 2000. *Oecologia* **124**: 476–486.

Castro-Diez, P., Villar-Salvador, P., Perez-Rentome, C. et al. 1997. *Trees* **11**: 127-134.

Cernusak, L.A., Aranda, J., Marshall, J.D., et al. 2007. *New Phytol.* **173**: 294-305.

Chapin, F.S. III., Johnson, D.A., McKendrick, J.D. 1980. *J. Ecol*. **68**: 189-209.

Chapin, F.S. III., Kedrowski, R.A. 1983. *Ecology* **64**: 376-391.

Chave, J., Muller-Landau,H.C., Baker T.R., et al. 2006. *Ecol. Appl*. **16**: 2356–2367.

Chen, T-H., Sheu, B-H., Chang, C-T. 1998. *Taiwan J. For. Sci*. **13**: 335-349 (in Chinese with English summary)

Clearwater, M.J., Susilawaty, R., Effendi, R., et al. 1999. *Oecologia* **121**: 478-488.

Clement, A., Montpied, P. 1997. A5 National Report: France. EC, UN/ECE, Austrian Federal Research Centre, 1997, 207 pp., pp. 100-101.

Cole, D.W., Rapp, M. 1981. Element cycling in forest ecosystems. In: Dynamic Properties of Forest Ecosystems. Cambridge University Press, Cambridge, pp. 341-409.

Coleman, Mark D.; Chang, S.X.; Robison, D.J. 2003. *Comm. Soil Sci. and Plant Analysis* **34**: 1919–1941.

Compton, J.E., Cole D.W., Homan, P.S. 1997. *Can. J. For. Res*. **27**: 662-666.

Cordell, S., Goldstein, G., Meinzer, F.C., et al. 2001. *Oecologia* **127**: 198-206.

Cornelissen JHC. et al. 1997. *Oecologia* **111**: 460-469.

Coulson, J.C., Butterfield, J. 1978. *J. Ecol*. **66**: 631-650.

Courtois, M., Masson, P. 1999. *Ann. For. Sci*. **56**: 521-52.

Covelo, F., Gallardo, A. 2001. *Can. J. Bot.* **79**: 1262-1269.

Cromack, K., Monk, C.D. 1975. Litter production, decomposition, and nutrient cycling in a mixed hardwood watershed and a white pine watershed. In: Howell, F.G., Gentry, J.B., Smith, M.B. (Eds.) Mineral Cycling in Southeastern Ecosystems. Proceedings of a symposium held at Augusta, Georgia, May 1-3, 1974. Technical Information Center, Office of Public Affairs, U.S. Energy Research and Development Administration, pp. 609-624.

Cunningham, S.A., Floyd, R.B. 2004. *Can. J. For. Res*. **34**: 642–648.

Cunningham, S.A., Summerrhayes, B., Westoby, M. 1999. *Ecol. Monog.* **69**: 569-588.

Czerney, P., Fiedler, H.J. 1968. *Arch. Forstwes*. **17**: 1203-1278.

Czerny, P., Fiedler, H.J. 1969. *Arch. Forstwes*. **18**: 37-40.

Davidson, EA, Reis de Carvalho, CJ., Figueira, AM., et al. 2007. *Nature* **447**: 995-998.

Davidson, R., Gagnon, D., Mauffette, Y. 1999. *Plant and Soil* **208**: 135-147.

Davies, S.J. 1998. *Ecology* **79**: 2292-2308.

Davis, D.D., Skelly, J.M., Nash, B.L. 1995. Elemental concentrations in foliage of red maple, red oak, and white oak in relation to atmospheric deposition in Pennsylvania. In: Gottschlak, K.W., Fosbroke, S.L.C.(eds.) Proceedings 10th Central Hardwood Forest Conference, Morgentown, West Virginia, March 5-8, 1995. US Department of Agriculture, Forest Service, Northeastern Forest Experiment Station, General Technical Report NC-197, pp. 188-195.

Day, F.P.Jr., McGinty, D.T. 1975. Mineral cycling strategies of two deciduous and two evergreen tree species on a southern Appalachian watershed. In: Howell, F.G., Gentry, J.B., Smith, M.B. (eds.) Mineral Cycling in Southeastern Ecosystems. Proceedings of a symposium held at Augusta, Georgia, May 1-3, 1974. Technical Information Center, Office of Public Affairs, U.S. Energy Research and Development Administration, pp. 736-743.

DeBell, D.S., Radwan, M.A. 1984. *Plant and Soil* **77**: 391-394.

Dell B, Bywaters T. 1989. *Can. J. For. Res.* **19**: 427-431.

Dell B, Malajczuk M. 1994. *Can. J. For. Res.* **24**: 2409-2416.

DeLucia, E.H., Thomas, R.B. 2000. *Oecologia* **122**: 11-19.

Demchik M.C., Sharpe, W.E. 2000. *Forest Ecol. Manage*. **136**: 199-207.

DeWalt, S.J., Denslow, J.S., Hamrick, J.L. 2004. *Oecologia* **138**: 521–531.

Dietz, H., Wirth, L.R., Buschmann, H. 2004. *Biological Inv*. **6**: 511–521.

du Toit, J.T., Bryant, J.P., Frisby, K. 1990. *Ecology* **7**: 149-154.

Duguma, B. 1995. Growth of nitrogen fgixing trees of moderate to very acid soils of the humid lowlands of southern Cameroon. In: D.O. Evans, L.T. Szott, Nitrogen Fixing Trees for AcidSolis. Proceedings of a Wirkshop Sponsored by Nitrogen Fixing Tree Association, Centro Agronomico Tropical de Investigacion y Ensenanza, July 3-8, 1994, Turrialba, Costa Rica. A publication of the Nitrogen Fixing Tree Asspociation, Nitrogen Fixing Tree Research Reports, Special Issue. 195-206 pp.

Dushkov, V.Yu. 1980. *Pochvovedenie* **3**: 74-82.

Duvigneaud, P., Denaeyer-de Smet, S. 1967. Biomass, productivity, and mineral cycling in deciduous forests Belgium. In: Symposium on Primary Productivity and Mineral Cycling in Natural Ecosystems. University of Maine Press. pp. 167-186.

Duvigneaud, P., Denaeyer-de Smet, S. 1970. Ecological cycling of minerals in temperate decdiduous forests. In: D.E. Beichle (ed.) Analysis of Temperate Forest Ecosystems. Springer-Verlag, Berlin, pp. 199-225.

Eamus, D., Myers, B., Duff, G., et al. 1999. *Photosynthetica* **36**: 575-586.

Eamus, D., Prichard, H. 1998. *Tree Physiol*. **18**: 537-545.

Elliott, K.J., Boring, L.R., Swank, W.T. 2002. *Can. J. For. Res*. **32**: 667-683.

Ellis RC. 1975. *Can. J. For. Res.* **5**: 310-317.

Ellis RC. 1979. *Can. J. For. Res.* **9**: 179-188.

Erdmann GG. et al. 1988. *Can. J. For. Res.* **18**: 134-139.

Ermolova, L.S., Utkin, A.I. 1998. *Russian J. Ecol*. **29**: 152-156.

Escarre, A., Roda, F., Terradas, J., Mayor, X. 1999. Nutrient distribution and cycling. In: Roda, F., Retana, J., Garcia, C.A., Bellot, J. (eds) Ecological Studies, vol. 137. Ecology of Evergreen Oak Forests, pp. 253-269.

Evdokimova, T.I. 1955. Pochvovedenie **6**: 53-59.

Ewald, J. 2000. *Forstw. Cbl.* **119**: 276-296.

Fahey, T.J., Battles, J.J., Wilson, G.F. 1998. *Ecol. Monogr*. **68**: 183-212

Falkelgren-Grerup, U., ten Brink, D-J., Bruner, J. 2006. *Forest Ecol. and Manage*. **225**: 74-81.

Farmer, R.E., Jr., Bengtson, G.W., Curlin, J.W. 1970. *Forest Sci*. **16**: 130-136.

Feller, I.C. 1995. Ecol. Monogr. **65**: 477-505.

Ferm, A. 1985. *Folia Forest*. **641**: 1-35.

Ferm, A., Markkola, A. 1985. *Folia Forest*. **613**: 1-28.

Fiedler, H.J., Czerney, P. 1970. *Arch. Forstwes*. **19**: 963-979.

Fiedler, H.J., Hunger, W., Wiesner, J. 1974. *Beitr. Forstw*. **1**: 17-24.

Filip, V., Dirzo, R., Maass, J.M. et al. 1995. *Biotropica* **27**: 78-86.

Flanagan PW, Van Cleve K. 1983. *Can. J. For. Res.* **13**: 795-817.

Flexas, J., Gulias, J., Jonasson, S. et al. 2001. *Acta Oecol*. **22**: 33-43.

Fonte, S.J., Schowalter, T.D. 2004. *Biotropica* **36**: 474–482.

Foulds, W. 1993. *New Phytol*. **125**: 529-546.

Gallardo, J.F., Martin, A., Moreno, G. 1999. *Ann. For. Sci*. **56**: 321-331.

Gaoming, J., Haiping, T., Mei Y. et al. 1999. *Trees* **14**: 72-82.

García, L.V., Marañón, T., Ojeda, F., et al. 2002. *Oikos*: **98**: 75-86.

Garnier, E., Laurent, G., Bellmann, A., et al. 2001. *New Phytol.* **152**: 69-83.

Gerloff, G.C., Moore, D.G., Curtis, J.T. 1964. Mineral content of native plants of Wisconsin. Wisconsin Agr. Exp. Station Research Report **14**: 3-27.

Givnish, T.J., Montgomery, R.A., Goldstein, G. 2004. *Am. J. Bot*. **91**: 228-246.

Goncalves-Alvim, S.J., Collevatti, R.G., Fernandes, G.W. 2004. *Ann. Bot*. **94**: 259-268.

Gowda, J.H., Palo, R.T. 2003. *Afr. J. Ecol*. **41**: 218-223.

Gower, S.T., Reich, P.B, Son, Y. 1993. *Tree Phys*. **12**: 327-345.

Gratani, L., Bombelli, A. 2001. *Ann. Bot. Fenn*. **38**: 15-24.

Gratani, L., Varone, L. 2004. *Flora* **199**: 58–69.

Gray, J.T. 1983. *J. Ecol*. **71**: 21-41.

Grigal, D.F., Ohmann, L.F., Moody, N.R. 1979. Nutrient content of some tall shrubs from northeastern Minnesota. U.S. Dep. Agric. For. Serv. Res. Pap. NC-168., 10 p.

Grimshaw H.M., Allen, S.E. 1987. *Vegetatio* **70**: 157-169.

Grubb, P.J., Edwards, P.J. 1982. *J. Ecol*. **70**: 623-648.

Gulder, H.-j., Kölbel, M. 1993. Waldbodeninventur in Bayern. Forstliche Forschungsberichte München, 132, 243 pp. [in German with English summary].

Gulias, J., Flexas, J., Mus, M., et al. 2003. *Ann. Bot*. **92**: 215-222.

Hagen-Thorn, A., Armolaitis, S., Callesen, I., et al. 2004. *Ann. For. Sci*. **61**: 489-498.

Hallett, R.A., Hornbeck, J.W. 1997. *Can. J. For. Res*. **27**: 1233-1244.

Han, W., Fang, J., Guo, D., et al. 2005. *New Phytol.* **168**: 377-385.

Hanley, M.E., Lamont, B.B. 2002. *Funct. Ecol*. **16**: 216-222.

Harrington, R.A., Fownes, J.H., Vitousek, P.M. 2001. *Ecosystems* **4**: 646-657.

He, J.-S., Wang, X., Schmid, B., et al. 2010. *J. Plant Res*. **123**: 551-561.

He, J.-S., Wang, Z.H., Wang, X.P., et al. 2006. *New Phytol.* **170**: 835-848.

Heal, O.W., Smith, R.A.H. 1978. 1. Introduction and site description. In: Heal, O.W. and Perkins, D.F. (Eds.). Production Ecology of British Moors and Montane Grasslands. Ecological Studies 27, Springer-Verlag, Berlin, Heidelberg, New York, pp. 3-16.

Heinsdorf, D. 1984. *Beitr. Forstwir*. **18**: 28-36.

Heinsdorf, D. 1985. *Beitr. Forstwir*. **19**: 34-40.

Heinsdorf, D. 1987. *Beitr. Forstwir*. **21**: 13-17.

Heinsdorf, D., Krauss, H.H. 1974. *Beitr. Forstwir*. **1**(1974): 25-37.

Henry, D.G. 1973. Minnesota Forestry Res. Notes. **241**: 1-4

Hevia, F., Minolletti, L., Decker, K.L.M. et al. 1999. *Am. J. Bot*. **86**: 447-455.

Hiremath, A.J. 2000. *Tree Physiol*. **20**: 937-944.

Hiremath, A.J., Ewel, J.J., Cole, T.G. 2002. *Forest Sci*. **48**: 662-672.

Hobbie, S.E., Gough, L. 2002. *Oecologia* **131**: 453-462.

Hofmeister, J., Mihajlevic, M., Hosek, J. et al. 2002. *Forest Ecol. Manage*. **169**: 213-230

Höhne, H. 1962. *Arch. Forstwes*. **11**: 1085-1114.

Höhne, H. 1963. *Arch. Forstwes*. **12**: 792-805.

Hölscher D., Leuschner, C., Bohman, K., et al. 2004. *J. Trop. Ecol*. **20**: 157-164.

Hölscher, D. 2004. *Basic Appl. Ecol*. **5**: 163-172.

Hölscher, D., Hertel, D., Koenies, H. 2002. *J. Pl. Nutr. and Soil Sci*. **165**: 668-674.

Hölscher, D., Leuschner, C., Bohman, K., et al. 2004. *J. Trop. Ecol*. **20**: 157-164.

Huang, J., Wang, X., Yan, E. 2007. *Forest Ecol. Manage*. **239**: 150-158.

Huguet, C. 1987c. Pears. In Martin-Prevel, P., Gagnard, J., Gautier, P. (Eds.) Plant Analysis as a guide to the nutrient requirements of temperate and tropical crops. Lavoisier Publishing Inc. New York., pp. 230

Huorang, W., Wenlong, Z. 1996. Fertilizer and eucalypt plantations in China. In: P.M. Attiwill and M.A. Adams. Nutrition in Eucalypts. CSIRO, Collingwood, Australia, pp. 389-397.

Ignatenko, I.V., Pugachev, A.A. 1980. *Pochvovedenie* **8**: 43-54.

Ilyin, V.B. 1974. *Pochvovedenie* **6**: 89-95.

Jiang, G.M., Tang, H.P., Yu, M., et al. 1999. *Trees* **14**: 72–82

Johnson, J.E., Mitchem, D.O., Kreh, R.E. 2002. The relationship between soils and foliar nutrition for planted royal paulownia. In: Outcalt, Kenneth W., ed. 2002. Proceedings of the eleventh biennial southern silvicultural research conference. Gen. Tech. Rep. SRS–48. Asheville, NC: U.S. Department of Agriculture, Forest Service, Southern Research Station. 622 p.

Jokela, E.J., Shannon, C.A., White, E.H. 1981. *Can. J. For. Res.* **11**: 299-305.

Judd, T.S., Bennett, L.T. Weston, C.J. et al. 1996. *For. Ecol. Manage*. **82**: 87-101

Juhrbandt, J., Leuschner, C., Hölscher, D. 2004. *For. Ecol. Manage*. **202**: 245-256.

Jurkevich, I.D., Jaroschevich, E.P., Gold, D.S. 1972. *Lesovedenie* **5**: 3-13.

Kallio, P. 1975. Kevo, Finland. In: Rosswall, T., Heal, O.W. (Eds.) Structure and Function of Tundra Ecosystems. *Ecol. Bull*. (Stockholm) **20**: 193-223.

Kappelle, M., Leal, M.A. 1996. *Biotropica* **28**: 331-344.

Karmanova, I.V., Sudnitsyna, T.N. 1987. *Pochvovedenie* **9**: 79-88.

Kass, D.L. 1995. Are nitrogen fixing trees a solution for acid soils? In: D.O. Evans, L.T. Szott, Nitrogen Fixing Trees for Acid Solis. Proceedings of a Workshop Sponsored by Nitrogen Fixing Tree Association, Centro Agronomico Tropical de Investigacion y Ensenanza, July 3-8, 1994, Turrialba, Costa Rica. A publication of the Nitrogen Fixing Tree Asspociation, Nitrogen Fixing Tree Research Reports, Special Issue. 19-31 pp.

Katahata, S., Naramoto, M., Kakubari, et al. 2005. *Tree Physiol*. **25**: 437–445.

Kazda, M., Salzer, J., Reiter, I. 2000. *Tree Physiol*. **20**: 1029-1037.

Kennedy, H.E., Jr. 1993. Effects of Crown Position and Initial Spacing on Foliar Nutrient Composition of Seven Bottomland Hardwoods. USDA Forest Service, Southern Forest Experiment Station, Research Note SO-371,

Khodjamkuliev, A. 1977. *Pochvovedenie* **11**: 128-133.

Khojamkuliev, A. 1976. *Pochvovedenie* **1**: 109-117.

Killingbeck, K., Whitford, W.G. 1996. *Ecology* **77**: 1728-1737.

Kimmins, J. P., Catanzaro, J. de, Binkley, D. 1979. Tabular summary of data from the literature on the biogeochemistry of temperate forest ecosystems. Vancouver, B.C. Faculty of Forestry, Univ. of British Columbia.

Kimura, K., Ishida, A., Uemura, A. et al. 1998. *Tree Physiol*. **18**: 459-466.

Kindu, M., Glatzel, G., Tadesse, Y., et al. 2006. *J. Trop. For. Sci*. 18: 173-180.

King, J.A., Campbell, B.M. 1994. *For. Ecol. Manage*. **67**: 225-239.

Kitajima, K., Mulkey, S.S., Wright, S.J. 1997. *Oecologia* **109**: 490–498.

Kitajima, K., Mulkey, S.S., Wright, S.J. 2005. *Ann Bot*. **95**: 535-547.

Klimo, E. 1981. *Acta Ecol.* **23**: 6-51.

Kloeppel, B.D., Abrams, M.D. 1995. *Tree Physiol*. **15**: 739-746.

Knight, C.A., Ackerly, D.D. 2003. *New Phytol*. **160**: 337-347.

Knight, P.J. and Nicholas, I.D. 1996. Eucalypt nutrition: New Zealand experience. In: P.M. Attiwill and M.A. Adams. Nutrition in Eucalypts. CSIRO, Collingwood, Australia, pp. 275-302.

Knops, J.M.H., Koenig W.D. 1997. *Plant Ecol*. **130**: 121-131.

Kogami, H., Hanaba, Y.T., Kibe, T. et al. 2001. *Plant Cell Environ.* **24**: 529-538.

Koretskaya, L.B. 1970. *Lesovedenie* **5**: 70-75.

Korneev, V.P. 1959. *Pochvovedenie* **4**: 87-94.

Kuers, K., Steinbeck, K. 1998. *Can. J. For. Res*. **28**: 1660-1670

Kull, O., Koppel, A., Noormets, A. 1998. *Tree Physiol*. **18**: 45-51

Lal, C.B., Annapurna, C., Raghubanshi, A.S. et al. 2001. *Can. J. Bot*. **79**: 1066–1075

Lambrecht-Mcdowell, S.C., Radosevich, S.R. 2005. *Biol. Invasions* **7**: 281-295.

Lang GE, et al. 1982. *Can. J. For. Res.* **12**: 311-318.

Lavergne, S., Garnier, E., Debussche, M. 2003. *Ecol. Lett*. **6**: 398-404.

Le Thiec, D., Dixob, M., Loosveldt, P., et al. 1996. *Trees* **10**: 55-62.

Lee, W.K., Lee, C.H., Kwang, C.H. et al. 1993. *Res. Rep. For. Res. Inst*. **48**: 27-35.

Li, M.S., 1997. *Estaurine, Costal and Shelf Sci*. **45**: 463-472.

Likens, G.E., Bormann, F.H. 1970. Bulletin of the School of Forestry, Yale University **79**: 1-25.

Lin, K.C., Du, C.T., Huang, C.M. 2003. *Taiwan J. For. Sci*. **18**: 95-100.

Lin, P., Wang W. 2001. *Ecol. Eng*. **16**: 415-424.

Liu, W., Fox, J.E.D., Xu, Z. 2002. *For. Ecol. Manage*. **158**: 223-235.

Liu, X., Ellsworth, D.S., Tyree, M.T. 1997. *Tree Physiol*. **17**: 169-178.

Llorens, L., Penuelas, J., Fillela, I. 2003. *Physiol. Plant*. **118**: 84-95.

Lockheart, M.J., Van Bergen, P.F., Evershed, R.P. 1997. *Org. Geochem.* 26: 137-153.

Londhiyal, L.S. et al. 1994. *Can. J. For. Res.* **24**: 1199-1209.

Loranger, J., Shipley, B. 201. *Botany* **88**: 30-38.

Loue, A., Gagnard, J., Morard, P. 1987. Vines. In Martin-Prevel, P., Gagnard, J., Gautier, P. (Eds.) Plant Analysis as a guide to the nutrient requirements of temperate and tropical crops. Lavoisier Publishing Inc. New York., pp. 175-206

Loue, A., Gagnard, J., Morard, P. 1987. Vines. In Martin-Prevel, P., Gagnard, J., Gautier, P. (Eds.) Plant analysis as a guide to the nutrient requirements of temperate and tropical crops. Lavoisier Publishing Inc. New York., pp. 175-206

Lovelock, C.E., Feller, I.C., Mckee, K.L., et al. 2004. *Funct. Ecol*. **18**: 25-33.

Luk'yanets, V.B. 1980. *Lesovedenie* **1**: 52-57.

Lusk, C.H., Wright, I., Reich, P.B. 2003. *New Phytol*. **160**: 329-336.

Ma, J.-Y., Fang, X.-W., Xia, D.-S., et al. 2008. *J. Plant Ecol*. (*Chinese Version*) **32**: 848-857.

Maghembe, J.A., Prins, H. 1994. *For. Ecol. Manage*. **64**: 171-182.

Manakov, K.M. 1968. *Pochvovedenie* **1**: 80-92.

Manakov, K.N. 1961. *Pochvovedenie* **7**: 34-41.

Manakov, K.N. 1970. *Lesovedenie* **4**: 27-36.

Mankovska B. 1997. *Lesnictvi* **43**: 117-124

Marchal, J. 1987. Date palm. In Martin-Prevel, P., Gagnard, J., Gautier, P. (Eds.) Plant Analysis as a guide to the nutrient requirements of temperate and tropical crops. Lavoisier Publishing Inc. New York., pp. 405-418

Marchenko, A.I., Karlov, E.M. 1962. *Pochvovedenie* **7**: 52-66.

Markov, M.I., Glaser, B., Zech, W., et al. 2003. *Plant and Soil* **256**: 389-402.

Marrs, R.H. 1978. *J. Ecol*. **66**: 533-545.

Marrs, R.H., Proctor, J. 1978. *J. Ecol*. **66**: 417-432.

Martin-Prevel, P., Gagnard, J., Gautier, P. (Eds.) 1987. Plant analysis as a guide to the nutrient requirements of temperate and tropical crops. Lavoisier Publishing Inc. New York.

Martin, J., Kloeppel, B.D., Schaefer, T.L., et al. 1998. *Can. J. For. Res*. **28**: 1648-1659.

Martinalli, L.A., Almeida, S., Brown, I.F., et al. 2000. *Biotropica* **32**: 597-613.

Martizez- Sanchez, J.L. 2003. *J. Trop. Ecol*. **19**: 465-468.

Matson, P., Johnson, L., Billow, C. et al. 1994. *Ecol. Appl*. **4**: 280-298.

Matsuki, S., Koike, T. 2006. *Ann. Bot*. **97**: 813-817.

Mayor, X., Roda, F. 1992. *Vegetatio* **99/100**: 209-217.

McBride J.R., Norberg, E., Bertenshaw, J., et al. 1997. Proceedings of a symposium on oak woodlands: ecology, management, and urban interface issues; 19-22 March 1996. Gen. Techn. Rep. PSW-GTR-160. Albany, CA: Pacific Southwest Research Station, Forest Service, U.S. Department of Agriculture, pp. 117-125.

McLennan DS. 1990. *Can. J. For. Res.* **20**: 1089-1097.

McPherson, S., Eamus, D., Murray, B.R. 2004. *Austr. J. Bot*. **52**: 293-301.

Mediavilla, S., Escudero, A. 2003. *Plant Ecol.* **168**: 321-332.

Meir, P., Levy, P.E., Grace, J., et al. 2007. *Plant Ecol*. **192**: 277-287.

Melendez, G., Szott, L.T., Rocse, A. 1995. Mineralizacion de nitrogeno de mater foliar de especies de Inga. In: D.O. Evans, L.T. Szott, Nitrogen Fixing Trees for Acid Solis. Proceedings of a Workshop Sponsored by Nitrogen Fixing Tree Association, Centro Agronomico Tropical de Investigacion y Ensenanza, July 3-8, 1994, Turrialba, Costa Rica. A publication of the Nitrogen Fixing Tree Association, Nitrogen Fixing Tree Research Reports, Special Issue. 35-41 pp.

Merino, A., Balboa, M.A., Rodriguez Soalleiro, R., et al. 2005. *For. Ecol. Manage*. **207**: 325-339.

Merino, A., Lopez, A.R., Branas, J., et al. 2003. *Ann. For. Sci*. **60**: 509-517.

Midgley, J.J., Van Wyk, G.R., Everard, D.A. 1995. *Afr. J. Ecol*. **33**: 160-168.

Mina, V.I. 1955. *Pochvovedenie* **6**: 32-44.

Minotta, G., Pinzauti, S. 1996. *For. Ecol. Manage*. **86**: 61-71.

Misson, L., Ponette, Q., Andre, F. 2001. *Ann. For. Sci*. **58**: 699-712.

Mitchell, K., Bolstad, P.V., Vose, J.M. 1999. *Tree Physiol*. **19**: 861-870.

Mitrofanov, D.P. 1977. Khimicheskii sostav lesnykh rastenii Sibiri. Izd. Nauka, Sibirskoe Otdelenie, Novosibirsk, 120 pp. (in Russian).

Morrison IK. 1985. *Can. J. For. Res.* **15**: 179-183.

Myers, B.J., Benyon, R.G., Theiveyanathan, S. et al. 1998. *Tree Physiol*. **18**: 565-573

Naaf, T., Wulf, M. 2012. *Plant Ecol*. **213**: 431-443.

Nagel, J.M., Griffin, K.L. 2001. *Am. J. Bot*. **88**: 2252–2258.

Nagel, J.M., Griffin, K.L., Schuster, W.S.F., *et al*. 2002. *Tree Physiol*. **22**: 859–867.

Neff, J.C., Reynolds, R., Sanford R. L. Jr., et al. 2006. *Ecosystems* **9**: 879–893.

Negi, J.D.S., Sharma, S.C. 1996. Mineral nutrition and resource conservation in Eucalyptus plantations and other forest covers of India. In: P.M. Attiwill and M.A. Adams. Nutrition in Eucalypts. CSIRO, Collingwood, Australia, pp. 399-416.

Negi, K.S., et al. 1983. *Can. J. For. Res.* **13**: 1185-1196.

Nelson, L.E. et al. 1995. *Can. J. For. Res.* **25**: 298-306.

Nielsen, S.L., Enroquez S., Duarte, C.M. 1998. *Biol. Plantarum* **40**: 91-101.

Niinemets, Ü., Kull, K. 1994. *For. Ecol. Manage*. **70**: 1-10

Niinemets, Ü., Kull, K. 2003. *Acta Oecol*. **24**: 209-219.

Niinemets, Ü., Portsmuth, A., Truus, L. 2002. *Ann. Bot*. **89**: 191-204.

Northup, B.K., Zitzer, S.F., Archer, S., et al. 2005. *J. Arid Environ*. **62**: 23-43.

Nyathi, P., Campbell, B. 1994. *For. Ecol. Manage*. **64**: 259-264.

Nygren, P. 1995. *Tree Physiol*. **15**: 71-83.

Oates, J.F., Whitesides, G.H., Davies, A.G., et al. 1990. *Ecology* **71**: 328-343.

Ola-Adams, B.A. 1993. For. *Ecol. Manage*. **58**: 299-319.

Oleksyn, J., Karolewski, P., Giertych, M.J. et al. 1998. *New Phytol*. **140**: 239-249.

Oleksyn, J., Zytkowiak, R., Reich, P.B. et al. 2000. *Trees* **14**: 271-281.

Oliveira, G., Martins-Loução, M.A., Correia, O. et al. 1996. *Trees* **10**: 247-254.

Osada, N., Takeda, H., Kitajima, K., et al. 2003. *Oecologia* **137**: 181-187.

Ovington, J.D. 1956. *Forestry* **29**: 22-28.

Ovington, J.D. 1959. *Ann. Bot*. **23**: 229-239.

Ovington, J.D., Madwick, A.I. 1959. *Plant and Soil* **10**: 389-400.

Palmiotto, P.A., Davies, S.J., Vogt, K.A., et al. 2004. *J. Ecol*. **92**: 609–623.

Paoli, G.D. 2006. *J. Trop. Ecol*. **22**: 397–408.

Papp, B.L. 1985. Nutrient content of woody plants. In: P. Jakucs (ed.) Ecology of an oak forest in Hungary, Akademiai Kiado, Budapest, pp. 321-348.

Parshevnikov, A.L. 1962. *Trudy Inst. Lesa*. **52**: 196-209.

Parsons, A.N., Welker, J.M., Wookey, P.A. et al. 1994. *J. Ecol*. **82**: 307-318.

Pastor, J., Bockheim, J.G. 1984. *Ecology* **65**: 339-353.

Pate, J.S., Dell, B. 1984. Economy of mineral nutrients in sandplain species. In: Pate, J.S., and Beard, J.S. (eds) Kwongan: Plant Life of the Sandplain. Biology of South-West Australian Shrubland Ecosystem. University of Western Australia Press, Nedlands, Western Australia, pp. 227-252.

Patlai, I.N., Boiko, A.V. 1978. *Lesovedenie* **4**: 100-103.

Penkov, O.G. 1976. *Pochvovedenie* **10**: 70-81.

Penuelas, J., Estiarte, M. 1996. *Ocecologia* **109**: 69-73.

Perry, E., Hickman, G.W. 2001. *J. Arboricult*. **27**: 152-159.

Pfanz, H., Lomsky, B., Hynek, V. et al. 1993. Lesnictvi **39**: 222-230 (in Czech with English summary).

Ponder, F.Jr. 1993. Performance of hardwoods planted with autumn olive after removing prior cover. In: Gillespie, A.R., Parker, G.R., and Pope, P.E. (eds.). Proceedings 9th Central Hardwood Forest Conference, Purdue University, West Lafayette, Indiana, March 8-10, 1993. US Department of Agriculture, Forest Service, North Central Forest Experiment Station, General Technical Report NC-161, pp. 447-454.

Poorter, H., De Jong, R. 1999. *New Phytol*. **143**: 163-176.

Poorter, H., Evans, J.R. 1998. *Oecologia* **116**: 26-37

Poorter, L., Bongers, F. 2006. *Ecology* **87**: 1733–1743.

Posdnjakov, L.K. 1967. *Lesovedenie* **6**: 36-42.

Powers, J.S., Tiffin, P. 2010. *Funct. Ecol*. **24**: 927-936.

Prado, J.A., Toro, J.A. 1996. Silviculture of eucalypt plantations in Chile. In: P.M. Attiwill and M.A. Adams. Nutrition in Eucalypts. CSIRO, Collingwood, Australia, pp. 357-369.

Prescott. C.E., Coward, L.P., Weetman, G.F. et al. 1993. *For. Ecol. Manage*. **61**: 45-60.

Prior, L.D., Bowman, D.M.J.S., Eamus, D. 2004. *Funct. Ecol*. **18**: 707-718.

Prior, L.D., Emaus, D., Bowman, D.M.J.S. 2003. *Funct. Ecol*. ***17***: 504-515.

Pyankov, V.I., Kondratchuk, A.V., Shipley, B. 1999. *New Phytol*. **143**: 131-142

Pyavchenko, N.I. 1967. *Lesovedenie* **3**: 32-43.

Ralston, C.W., Prince, A.B. 1965. Accumulation of dry matter and nutrients in pine and hardwood forests in the Lower Piedmont of North Carolina. In: Youngberg, C.T. (Ed.) Forest-Soil Relationships in North America. Papers presented at the Second North American Forest Soil Conference, August 1963, Oregon State University, Corvallis, Oregon, Oregon State University Press, Corvallis, pp. 77-104.

Ranger, J., Colin-Belgrand, M. 1966. *For. Ecol. Manage*. **86**: 259-277.

Rapp, M., Derfoufi, F.E., Blanchard, A. 1992. *Vegetatio* **99**/**100**: 263-272.

Read, J., Sanson, G.D. 2003. *New Phytol*. **160**: 81-99.

Reich, P.B. et al. 1994. *Oecologia* **97**: 82-92.

Reich, P.B., Buschena, C., Tjoelker, M.G., et al. 2003. *New Phytol*. **157**: 617-631.

Reich, P.B., Ellsworth, D.S., Walters M.B. et al. 1999. *Ecology* **80**: 1955-1969.

Reich, P.B., Walters, M.B., Ellsworth D.S. et al. 1998. *Oecologia* **114**: 471-482.

Remezov, N.P. 1959. *Pochvovedenie* **1**: 71-79.

Remezov, N.P., Bykova, L.N. 1953. *Pochvovedenie* **8**: 28-41.

Ricklefs, R.E., Matthew, K.K. 1982. *Can. J. Bot*. **60**: 2037-2045.

Rikala, R., Petälistö R-L. 1986. *Folia Forest*. **642**: 1-16.

Robert, B., Bertoni, G., Sayag, D. et al. 1969. *Comm. Soil. Sci. Plant Anal*. **27**: 2091-2109.

Rode, M.W. 1993. *J. Veg. Sci*. **4**: 263-268.

Rodin, L.E., Bazilevich, N.I. 1965. Dinamika organicheskogo veschcestva I biologicheskij krugovorot zol'nykh elementov i azota v osnovnykh tipakh rastitel'nostyi zemnogo shara. Nauka, Moskva-Leningrad, 1965.

Roggy, J.C., Prevost, M.F., Garbaye, J., et al. 1999. *J. Trop. Ecol*. **15**: 1-22.

Rosecrance, R.C., Weinbaum, S.A., Brown, P.H. 1998. *Ann. Bot*. **82**: 463-470.

Rudneva, E.N., Tonkogonov, V.D., Dorokhova, K.Ya. 1966. *Pochvovedenie* **3**: 14-26.

Rundel, P.W., Gibson, A.C., Midgley, G.S., et al. 2003. *Plant Ecol*. **169**: 179-193.

Rysin, L.P., Antyukhina, V.V. 1977. *Lesovedenie* **1**: 36-47.

Rzeznik, Z., Nebe, W. 1987. *Beitr. Forstwirt*. **21**: 106-110.

Saarsalmi, A., Palmgren, K., Levula, T. 1985. *Folia Forest*. **628**: 1-24.

Saarsalmi, A., Palmgren, K., Levula, T. 1991. Folia Forest. **768**: 1-25.

Salzer J. 2004. Structural and nutritional differences between climbers and their supporting trees in a montane rainforest in South-Ecuador. Dissertation Thesis (Department of Systematic Botany and Ecology, University of Ulm), 144 pp.

Samusenko, V.F., Golovina, R.D. 1989. *Pochvovedenie* **11**: 135- 141.

Sanchez, M.-J. et al. 1997. Chemical and physical analyses of selected plants and soils from Puerto Rico (1981-1990). U.S. Dept. of Agriculture, Forest Service, International Institute of Tropical Forestry, 112 p.

Santa Regina I. 2000. *Ann. For. Sci*. **57**: 691-700.

Santa Regina, I., Tarazona, T. 2001. *Forestry* **74**: 11-28.

Santa Regina, I., Tarazona, T., Calvo, R. 1997. *Plant Ecol*. **133**: 49-56.

Santiago, L.S., Goldstein, G., Meinzer, F.C., et al. 2004. *Oecologia* 140: 543-550.

Santiago, L.S., Wright, S.J. 2007. *Funct. Ecol*. **21**: 19-27.

Scarano, F.R., Duarte, H.M., Franco, A.C., et al. 2005. *Trees* **19**: 497-509.

Scatena, F.N., Silver, W., Siccama, T. et al. 1993. *Biotropica* **25**: 15-27.

Schroeder, L.A. 1986. *Ecology* **67**: 1628-1636.

Schulze, E-D., Kelliher, F.M., Körner, Ch. et al. 1994. *Annu. Rev. Ecol. Syst*. **25**: 629-660.

Scotter, G.W. 1965. *Can. J. Plant Sci*. **45**: 246-250.

Scotter, G.W. 1972. *Arctic* **25**: 21-27.

Searson, M.J., Thomas, D.S., Montagu, K.D., Conroy, J.P. 2004. *Funct. Plant Biol.* **31**: 441-450.

Seastedt, T.R., Crossley, D.A. Jr., Hargrove, W.W. 1983. *Ecology* **64**: 1040-1048.

Semenova, V.G. 1971. *Pochvovedenie* **7**: 46-55.

Sharma BM. 1983. *Can. J. For. Res.* **13**: 556-562.

Shaver, G.R. 1983. 16. Mineral nutrient and nonstructural carbon pools in shrubs from Mediterranean-type ecosystems in California and Chile. In: Kruger, F.J., Mitchell, D.T., Jarvis, J.U.M. (Eds). Ecological Studies 43, Springer-Verlag, Berlin, Heidelberg, New York, Tokyo, pp. 286-299.

Silfverberg K. 1982. *Folia Forest.* **526**: 1-12.

Silfverberg, K., Issakainen, J. 1991. *Folia Forest*. **769**: 1-23.

Silla, F., Escudero, A. 2004. *Funct. Ecol*. **18**: 511–521.

Simonic, P., Kalan, P. 1997. A14 National Report: Slovenia. In: Forest Foliar Condition in Europe. UN Ecxonomic Comission for Europe, European Commission. EC-UN/ECE-FBVA, Brussels, Geneva, Vienna (ISBN 3-901347-05-4), pp. 119-122

Singh, G. 1996. *For. Ecol. Manage*. **80**: 267-278.

Sprecht, R.L. 1988. Mediterranean -type ecosystems: A data source book. Kluwer Academic Publishers, Dordrechr, Boston, London, ISBN 90-61-93-652-7.

Springer, C.J., DeLucia, E.H., Thomas, R.B. 2005. *Tree Physiol*. **25**: 385–394.

Staaf, H., Stjernquist, I. 1986. *Scand. J. For. Res*. **1**: 333-342.

Stark, N. 1973. Nutrient Cycling in a Jeffrey pine Ecosystem. Monatana Forest and Conservation Experiment Station, Missoula, Montana, 389 pp.

Stefan, K., Fürst, A., Hacker, R., et al. 1997. EC, UN/ECE, Austrian Federal Research Centre, 207 pp.

Stepanets, I.T. 1963. *Pochvovedenie* **2**: 68-74.

Stone DM, Christenson DR. 1975. *Can. J. For. Res.* **5**: 410-413.

Sudnitsyna, T.N. 1987. *Lesovedenie* **2**: 26-35.

Sullivan, P.F., Welker, J.M. 2007. *Oecologia* **151**: 372-386.

Takagi, M., Gyokusen, K. 2004. *Urban For. Urban Green*. **2**: 167–171.

Tanner, E.V.J. 1977. *J. Ecol*. **65**: 883-918.

Tateno, R., Takeda, H. 2010. *Oecologia* **163**: 793-804.

Taylor, L. 1982. *J. Ecol*. **70**: 83-100.

Teklay, T. 2004. *Plant and Soil* **267**: 297-307.

Thomas, S.C., Bazzaz, F.A. 1999. *Ecology* **80**: 1607-1622.

Thompson K. et al. 1997. *New Phytol.* **136**: 679-689.

Thompson, J., Proctor, J., Viana, V., et al. 1992. *J. Ecol*. **80**: 689-703.

Tissue, D.T., Griffin, K.L., Turnbull, M.H., et al. 2005. *Tree Physiol*. **25**: 447-456.

Trabaud, L. 2001. *Ann. For. Sci*. **58**: 555-567.

Trillmich, H.-D. 1978. *Beitr. Forstwirt*. **2**: 73-78.

Trillmich, H.-D., Uebe, E.1976. *Beitr. Forstwirt*. **2**: 80-87.

Trillmich, H.-D., Uebel, E. 1982. Beitr. *Beitr. Forstwirt*. **16**: 34-39

Trillmich, H.G., Uebel, E. 1972. *Beitr. Forstwirt*. **4**, 15-24.

Turnbull, M.H., Whitehead, D., Tissue, D.T., et al. 2002. *Oecologia* **130**: 515-524.

Turnbull, M.H., Whitehead, D., Tissue, D.T., et al. 2003. *Funct. Ecol.* **17**: 101-114.

Turner, I.M., Ong, B.L., Tan, H.T. 1995. *Biotropica* **27**: 2-12.

Turner, J., Singer M.J. 1976. *J. Appl. Ecol*. **13**: 295-301.

Uri, V., Tullus, H. & Lõhmus, K. 2003. *Silva Fenn*. **37**: 301–311.

van Wijk, M.T., Williams, M., Shaver, G.R. 2005. *Oecologia* **142**: 421–427.

VanderSchaaf, C.L., Moore, J.A., Kingery., J.L. 2004. *For. Ecol. Manage*. **190**: 201-218.

Vassiljevskaja, V.D., Ivanov, V.V., Bogatyrev, L.G. et al. 1975. Agapa, USSR. In: Rosswall, T., Heal, O.W. (Eds.) Structure and Function of Tundra Ecosystems. Ecol. Bull. (Stockholm) **20**: 141-158.

Vendramini, F., Diaz, S., Gurvich, D.E., et al. 2002. *New Phytol*. **154**: 147-157.

Verry ES, Timmons DR. 1976. *Can. J. For. Res.* **6**: 436-440.

Villar, R., Merino, J. 2001. *New Phytol*. **151**: 213–226.

Vincent, G. 2001. *J. Trop. Ecol.* **17**: 495 –509.

Vitousek, P.M. 1998. *Ecosystems* **1**: 401-407.

Vitousek, P.M., et al. 1995. *Ecology* **76**: 712-720.

Volk, B.G., Schemnitz, S.D., Gamble, J.F., et al. 1975. Base-line data on Everglades soil-plant systems: elemental composition, biomass, and soil depth. In: Howell, F.G., Gentry, J.B., Smith, M.B. (Eds.) Mineral Cycling in Southeastern Ecosystems. Proceedings of a symposium held at Augusta, Georgia, May 1-3, 1974. Technical Information Center, Office of Public Affairs, U.S. Energy Research and Development Administration, pp. 658-672.

Vtorova, V.N. 1982. *Pochvovedenie* **8**: 112-120.

Wang J.-Y., Wang S.-Q., Li R.-L., et al. 2011. *Chinese J. Plant Ecol*. **35**: 587–595

Wang, J.R., Zhong, A.L., Simard, S.W. et al. 1996. *For. Ecol. Manage*. **82**: 27-38

Ward, W.W., Bowersox, T.W. 1970. *Forest Sci*. **16**: 113-120.

Warren, C.R., Tausz, M., Adams, M.A. 2005. *Tree Physiol*. **25**: 1369–1378.

White, E.H., Carter, M.C. 1970. Relationships between foliage nutrient levels and growth of young natural stands of Populus deltoides Bartr. In: Youngberg, C.T., and Davey, C. (eds.) Tree Growth and Forest Soils. Oregon State University Press, Corvallis ISBN 0-87071-423-6, pp. 283-294.

Whitehead, D., Boelman, N.T., Turnbull, M.H., et al. 2005. *Oecologia* **144**: 233-244.

Wielgolaski, F.E., Kjelvik, S., Kallio, P. 1975. Mineral content of tundra and forest tundra plants in Fennoscandia. In: Wielgolaski, F.E. (ed.) Fennoscandia Tundra Ecosystems. Part 1: Plants and Microorganisms. Ecological Studies 16, Springer-Verlag, New York, Heidelberg, Berlin, pp. 316-332.

Williams-Lindera, G. 2000. *Plant Ecol*. **149**: 233-244.

Wilmot, T.R. et al. 1995. *Can. J. For. Res.* **25**: 386-397.

Wilmot, T.R., Ellsworth, D.S., Tyree, M.T. 1996. *For. Ecol. Manage*. **84**: 123-134.

Wohlfahrt, G., Bahn, M., Haubner, E. et al. 1999. *Plant Cell Environ*. **22**: 1281-1296.

Wright, I.J., Reich P.B., Westoby, M., et al. 2004. *Nature* **428**: 821-827.

Wright, I.J., Reich, P.B., Westoby, M. 2001. *Funct. Ecol.* **15**: 423-434.

Wu, C.C. Tsui, C.-C., C.-F. Hseih, et al. 2007. *For. Ecol. Manage*. **239**: 81-91.

Wu, T.-G., Chen, B.-F., Xiao, Y.-H., et al. 2010. *Chinese J. Plant Ecol.* **34**: 58–63

Wyckoff, G.W., Einspahr, D.W., Benson, M.K. 1989. Development of an aspen sucker stand following irrigation and fertilization. In: Proceedings of the Aspen Symposium ’09, Adams R.D. (Ed.) Duluth, Minnesota July 25-27, 1989. USDA, Forest Service, North Central Forest Experiment Station, General Technical Report, NC-140, pp.195-203.

Yan, E.-R., Wang, X.-H., GUO, M., et al. 2010. *Chinese J. Plant Ecol.* **34**: 48–57.

Yarie, J, Van Cleve, K. 1996. *Ecol. Appl.* **6**: 815-827.

Yarie, J. 1997. *Ecology* **78**: 2351-2358.

Yasumura, Y., Onoda, Y., Hikosaka, K., et al. 2005. *Plant Ecol*. **178**: 29-37.

Yin X.-R., Liang C.-Z., Wang, L.-X., et al. 2010. *Chinese J. Plant Ecol.* **34**: 39–47.

Yuan, Z.-Y., Li, L.-H. Xing-Guo Han, X.-G., et al. 2005. *J. Arid Environ*. **63**: 191-202.

Zemlyanitskij, L.T. 1954. *Pochvovedenie* **12**: 30-35.

Zhang, J-G., Fu, S-L., Wen, D-Z., et al. 2009. *J. Trop. and Subtrop. Bot*. **17**: 395-400.

Zheng, S., Shangguan, Z. 2007. *Plant Ecol*. **191**: 279-293.

Zhu, A-D., Cao, K-F. 2010. *Oecologia* **163**: 591-599.
