# Supplementary material for: Phenotypic correlates of the lianescent growth form: a review
Source: Ann Bot. 2013 Oct 29;112(9):1667–81. doi: 10.1093/aob/mct236 (PMC3838560; doi:10.1093/aob/mct236)
Supplement: Supplementary Data [file supp_mct236_mct236supp_table.pdf]

SUPPLEMENTARY DATA

Table S1. Listing of woody angiosperm taxa used in analysis of leaf traits (Fig. 2 and 3) based on published literature sources (see Supplementary Information 1). Growth forms are given as in original sources; note that some taxa occur as more than one growth form. Family designation and taxon spelling have been verified against a publicly accessible online database (The Taxonomic Name Resolution Service. iPlant Collaborative. Version 3.0 accessed on 2013.01.26. Available at http://tnrs.iplantcollaborative.org).

Taxon	Growth form		
**ACANTHACEAE**			
Avicennia marina	shrub	tree	
Acanthus ilicifolius	shrub		
Acanthus montanus	shrub		
Hypoestes verticilliaris	shrub		
Isoglossa ciliata	shrub		
Justicia adhatoda	shrub		
Avicennia alba		tree	
Avicennia germinans		tree	
Avicennia lanata		tree	
**ACHARIACEAE**			
Caloncoba echinata		tree	
Kiggelaria africana		tree	
Lindackeria paludosa		tree	
**ACTINIDIACEAE**			
Saurauia veraguasensis		tree	
Actinidia arguta			liana
Actinidia callosa			liana
Actinidia chinensis			liana
Actinidia deliciosa			liana
Actinidia kolomikta			liana
Actinidia rufa			liana
**ADOXACEAE**			
Sambucus canadensis	shrub		
Sambucus mexicana	shrub		
Sambucus nigra	shrub		
Sambucus racemosa	shrub		
Sambucus sp.	shrub		
Sambucus williamsii	shrub		
Viburnum cassinoides	shrub		
Viburnum cinnamomifolium	shrub		
Viburnum costaricanum	shrub		
Viburnum dilatatum	shrub		
Viburnum opulus	shrub		
Viburnum rafinesquianum	shrub		
Viburnum rigidum	shrub		

Viburnum tinus	shrub	
Viburnum utile	shrub	
Viburnum sp.		tree
**AEXTOXICACEAE**		
Aextoxicon punctatum		tree
**ALTINGIACEAE**		
Altingia obovata		tree
Liquidambar formosana		tree
Liquidambar styraciflua		tree
Liquidambar styraciflua var. *mexicana*		tree
**ALZATAECAEA**		
Alzatea sp.		tree
**AMARANTHACEAE**		
Atriplex stipitata	shrub	tree
Allenrolfea patagonica	shrub	
Anabasis aphylla	shrub	
Anabasis brevifolia	shrub	
Anabasis salsa	shrub	
Atriplex argentina	shrub	
Atriplex cana	shrub	
Atriplex canescens	shrub	
Atriplex confertifolia	shrub	
Atriplex halimus	shrub	
Atriplex hymenelytra	shrub	
Atriplex hymenotheca	shrub	
Atriplex inflata	shrub	
Atriplex leucoclada	shrub	
Atriplex leucophylla	shrub	
Atriplex nummularia	shrub	
Atriplex vesicaria	shrub	
Camphorozma monspeliacum	shrub	
Ceratoides arborescens	shrub	
Ceratoides lanata	shrub	
Ceratoides latens	shrub	
Chenopodium oahuense	shrub	
Grayia spinosa	shrub	
Halocnemum strobilaceum	shrub	
Halostachys caspica	shrub	
Halothamnus acutifolius	shrub	
Haloxylon ammodendron	shrub	
Heterostachys ritteriana	shrub	
Iljinia regelii	shrub	
Kalidium caspicum	shrub	

Kalidium cuspidatum	shrub	
Kalidium foliatum	shrub	
Kalidium gracile	shrub	
Kochia prostrata	shrub	
Krascheninnikovia ceratoides	shrub	
Maireana carnosa	shrub	
Maireana pyramidata	shrub	
Maireana sedifolia	shrub	
Petrosimonia brachiata	shrub	
Rhagodia baccata	shrub	
Rhagodia gaudichaudiana	shrub	
Salsola chaudharyi	shrub	
Salsola cyclophylla	shrub	
Salsola dendroides	shrub	
Salsola laricifolia	shrub	
Salsola oppositifolia	shrub	
Salsola passerina	shrub	
Salsola tetrandra	shrub	
Suaeda australis	shrub	
Suaeda divaricata	shrub	
Suaeda microphylla	shrub	
Suaeda olufsenii	shrub	
Suaeda physophora	shrub	
Suaeda vera	shrub	
Sympegma regelii	shrub	
Traganum nudatum	shrub	
Atriplex marina		tree
Haloxylon persicum		tree
**ANACAMPSEROTACEAE**		
Grahamia bracteata	shrub	
**ANACARDIACEAE**		
Rhus chirendensis	shrub	tree
Rhus tomentosa	shrub	tree
Rhus typhina	shrub	tree
Lithraea caustica	shrub	
Pistacia lentiscus	shrub	
Pistacia terebinthus	shrub	
Rhus chinensis	shrub	
Rhus glabra	shrub	
Rhus microphylla	shrub	
Rhus ovata	shrub	
Rhus succedanea	shrub	
Anacardium excelsum		tree
Anacardium giganteum		tree

Astronium fraxinifolium		tree	
Astronium graveolens		tree	
Astronium lecointei		tree	
Astronium sp.		tree	
Buchanania lanzan		tree	
Buchanania obovata		tree	
Choerospondias axillaris		tree	
Harpephyllum caffrum		tree	
Lannea coromandelica		tree	
Lannea grandis		tree	
Lannea stuhlmannii		tree	
Lithraea molleoides		tree	
Mangifera foetida		tree	
Pistacia chinensis		tree	
Pistacia vera		tree	
Rhus sandwicensis		tree	
Schinopsis haenkeana		tree	
Semecarpus anacardium		tree	
Spondias mangifera		tree	
Spondias mombin		tree	
Spondias purpurea		tree	
Spondias radlkoferi		tree	
Swintonia schwenckei		tree	
Tapirira guianensis		tree	
Thyrsodium guianense		tree	
Trichoscypha patens		tree	
Toxicodendron diversilobum			liana
**ANNONACEAE**			
Polyalthia cerasoides	shrub		
Alphonsea mollis		tree	
Alphonsea monogyna		tree	
Annona coriacea		tree	
Annona purpurea		tree	
Annona reticulata		tree	
Annona spraguei		tree	
Annona squamosa		tree	
Bocageopsis multiflora		tree	
Bocageopsis sp.		tree	
Cymbopetalum baillonii		tree	
Desmopsis panamensis		tree	
Duguetia furfuracea		tree	
Duguetia lucida		tree	
Duguetia sp.		tree	
Guamia sp.		tree	

Guatteria cf. *poeppigiana*		tree
Guatteria dumetorum		tree
Guatteria megalophylla		tree
Guatteria poeppigiana		tree
Guatteria schomburgkiana		tree
Guatteria sp.		tree
Miliusa tomentosa		tree
Monanthotaxis caffra		tree
Monocarpia marginalis		tree
Onychopetalum lucidum		tree
Oxandra xylopioides		tree
Polyalthia lauii		tree
Polyalthia longifolia		tree
Polyalthia pendula		tree
Rollinia exsucca		tree
Sapranthus microcarpus		tree
Sapranthus palanga		tree
Xylopia nitida		tree
Xylopia sp.		tree
**APHLOIACEAE**		
Aphloia theiformis	shrub	
**APIACEAE**		
Bupleurum fruticosum	shrub	
Bupleurum rigidum	shrub	
Bupleurum salicifolium	shrub	
Ligusticum huteri	shrub	
Phellopterus littoralis	shrub	
**APOCYNACEAE**		
Beaufortia elegans	shrub	
Carissa carundu	shrub	
Carissa lanceolata	shrub	
Cynanchum thesioides	shrub	
Nerium oleander	shrub	
Poacynum hendersonii	shrub	
Strophanthus divaricatus	shrub	
Tabernaemontana coronaria	shrub	
Thevetia ovata	shrub	
Allamanda violacea		tree
Alstonia macrophylla		tree
Ambelania acida		tree
Aspidosperma album		tree
Aspidosperma cruentum		tree
Aspidosperma cylindrocarpon		tree
Aspidosperma megalocarpon		tree

Aspidosperma quebracho-blanco		tree	
Aspidosperma rigidum		tree	
Aspidosperma sp.		tree	
Aspidosperma tomentosum		tree	
Cerbera manghas		tree	
Couma guianensis		tree	
Funtumia africana		tree	
Funtumia latifolia		tree	
Himatanthus articulatus		tree	
Holarrhena floribunda		tree	
Kopsia officinalis		tree	
Lacmellea aculeata		tree	
Malouetia tamaquarina		tree	
Peschiera australis		tree	
Plumeria alba		tree	
Plumeria obtusa		tree	
Plumeria rubra		tree	
Rauvolfia paraensis		tree	
Rauvolfia vomitoria		tree	
Stemmadenia donnell-smithii		tree	
Stemmadenia obovata		tree	
Thevetia peruviana		tree	
Wrightia natalensis		tree	
Wrightia pubescens		tree	
Wrightia tomentosa		tree	
Alyxia sp.			liana
Anodendron affine			liana
Beaumontia grandiflora			liana
Ecdysanthera rosea			liana
Marsdenia lanata			liana
Marsdenia peniculata			liana
Matelea quirosii			liana
Melodinus novoguineensis			liana
Metaplexis japonica			liana
Periploca sepium			liana
Trachelospermum gracilipes			liana
**AQUIFOLIACEAE**			
Ilex verticillata	shrub	tree	
Ilex cassine	shrub		
Ilex discolor	shrub		
Ilex pallida	shrub		
Ilex pubescens	shrub		
Ilex tsoi	shrub		
Nemopanthus mucronatus	shrub		

Ilex anomala		tree	
Ilex aquifolium		tree	
Ilex canariensis		tree	
Ilex cochinchinensis		tree	
Ilex hylonoma		tree	
Ilex kengii		tree	
Ilex macfadyenii		tree	
Ilex mitis		tree	
Ilex opaca		tree	
Ilex platyphylla		tree	
Ilex rotunda		tree	
Ilex sideroxyloides		tree	
Ilex subcordata		tree	
Ilex triflora var. *kanehirai*		tree	
**ARACEAE**			
Dieffenbachia pittieri	shrub		
**ARALIACEAE**			
Acanthopanax senticosus	shrub		
Eleutherococcus senticosus	shrub		
Acanthopanax sciadophylloides		tree	
Arthrophyllum diversifolium		tree	
Cheirodendron trigynum		tree	
Cussonia spicata		tree	
Dendropanax arboreus		tree	
Didymopanax macrocarpus		tree	
Didymopanax morototoni		tree	
Didymopanax vinosum		tree	
Kalopanax pictus		tree	
Oreopanax capitatus		tree	
Oreopanax nubigenus		tree	
Oreopanax xalapensis		tree	
Schefflera decaphylla		tree	
Schefflera octophylla		tree	
Schefflera rodriguesiana		tree	
Schefflera sciadophyllum		tree	
Schefflera sp.		tree	
Hedera colchica			liana
Hedera helix			liana
**ARECACEAE**			
Geonoma cuneata	shrub		
Calyptrogyne costatifrons		tree	
Caryota ochlandra		tree	
Dictyocarium sp.		tree	
Calamus faberi			liana

Calamus simplicifolius		liana
Calamus sp.		liana
Daemonorops margaritae		liana
**ARISTOLOCHIACEAE**		
Aristolochia durior		liana
Aristolochia liukiuensis		liana
Aristolochia manshuriensis		liana
Aristolochia rotunda		liana
Aristolochia taliscana		liana
Aristolochia tomentosa		liana
**ASPARAGACEAE**		
Ruscus aculeatus	shrub	
**ASTERACEAE**		
Acamptopappus shockleyi	shrub	
Ajania tibetica	shrub	
Anvillea garcinii	shrub	
Argyranthemum spp.	shrub	
Aristomenia fruticosa	shrub	
Artemisia badhysi	shrub	
Artemisia californica	shrub	
Artemisia filifolia	shrub	
Artemisia frigida	shrub	
Artemisia halodendron	shrub	
Artemisia herba-alba	shrub	
Artemisia incana	shrub	
Artemisia intramongolica	shrub	
Artemisia leucotricha	shrub	
Artemisia maritima	shrub	
Artemisia meyeriana	shrub	
Artemisia ordosica	shrub	
Artemisia pamirica	shrub	
Artemisia pauciflora	shrub	
Artemisia rhodantha	shrub	
Artemisia santolinifolia	shrub	
Artemisia sp.	shrub	
Artemisia sphaerocephala	shrub	
Artemisia spinescens	shrub	
Artemisia taurica	shrub	
Artemisia tridentata	shrub	
Artemisia vestita	shrub	
Baccharis angustifolia	shrub	
Baccharis arbutifolia	shrub	
Baccharis articulata	shrub	
Baccharis latifolia	shrub	

Baccharis pedunculata	shrub
Baccharis pilularis	shrub
Baccharis salicifolia	shrub
Baccharis santelicis	shrub
Bahia ambrosioides	shrub
Brachyglottis revoluta	shrub
Brickellia arguta	shrub
Brickellia incana	shrub
Brickellia laciniata	shrub
Cassinia vauvilliersii	shrub
Chrysothamnus nauseosus	shrub
Chuquiraga jussieui	shrub
Chuquiraga spinosa	shrub
Cichorium spinosum	shrub
Cyclolepis genistoides	shrub
Diplostephium rupestre	shrub
Dittrichia viscosa	shrub
Dubautia scabra	shrub
Encelia californica	shrub
Encelia farinosa	shrub
Encelia frutescens	shrub
Flotovia diacanthoides	shrub
Flourensia cernua	shrub
Flourensia thurifera	shrub
Franseria dumosa	shrub
Gochnatia fascicularis	shrub
Gynoxys sp.	shrub
Haplopappus foliosus	shrub
Haplopappus glutinosus	shrub
Hazardia squarrosa. var. *squarrosa*	shrub
Helichrysum apiculatum	shrub
Helichrysum arenarium	shrub
Helichrysum elatum	shrub
Helichrysum stoechas	shrub
Hymenoclea salsola	shrub
Inula amphila	shrub
Inula cappa	shrub
Isocoma acradenia	shrub
Isocoma menziesii	shrub
Loricaria ilinissae	shrub
Myripnois dioica	shrub
Olearia decurrens	shrub
Olearia moschata	shrub
Olearia nummulariifolia	shrub

Olearia pimelioides	shrub		
Olearia virgata	shrub		
Phagnolam graceum	shrub		
Santolina chamaecyparissus	shrub		
Saussurea spp.	shrub		
Tetradymia glabrata	shrub		
Pentacalia lanceolifolia		tree	liana
Ageratina dendroides		tree	
Artocarpus hypargyraea		tree	
Ateleia herberth-smithii		tree	
Baccharis macrantha		tree	
Brachylaena neriifolia		tree	
Cassinia laevis		tree	
Dasyphyllum diacanthoides		tree	
Eupatorium critoniforme		tree	
Eupatorium viscidum		tree	
Flourensia campestris		tree	
Gochnatia floribunda		tree	
Heterothalamus alienus		tree	
Homalium dictyoneurum		tree	
Hopea dryobalanoides		tree	
Hopea pentanervia		tree	
Olearia avicenniifolia		tree	
Olearia erubescens		tree	
Persoonia linearis		tree	
Piptocarpha rotundifolia		tree	
Pollalesta discolor		tree	
Bidens reptans-urbanii			liana
Lasianthaea ceanothifolia			liana
Liabum caducifolium			liana
**ATHEROSPERMATACEAE**			
Laurelia sempervirens	shrub		
**AUSTROBAILEYACEAE**			
Austrobaileya scandens			liana
**BEGONIACEAE**			
Begonia luxurians	shrub		
**BERBERIDACEAE**			
Berberis amurensis	shrub		
Berberis circumserrata	shrub		
Berberis diaphana	shrub		
Berberis ilicifolia	shrub		
Berberis pinifolia	shrub		
Berberis poiretii	shrub		
Berberis pruinosa	shrub		

Berberis sp.	shrub	
Berberis trifoliolata	shrub	
Berberis vulgaris	shrub	
Mahonia lomariifolia	shrub	
Mahonia trifoliolata	shrub	
Nandina domestica	shrub	
**BETULACEAE**		
Alnus rugosa	shrub	tree
Betula populifolia	shrub	tree
Betula pumila	shrub	tree
Alnus crispa	shrub	
Betula alba	shrub	
Betula exilis	shrub	
Betula fruticosa	shrub	
Betula glandulosa	shrub	
Betula gmelinii	shrub	
Betula humilis	shrub	
Betula nana	shrub	
Corylus americana	shrub	
Corylus avellana	shrub	
Corylus cornuta	shrub	
Corylus heterophylla	shrub	
Ostryopsis davidiana	shrub	
Alnus glutinosa		tree
Alnus glutinosa x A. incana		tree
Alnus hirsuta		tree
Alnus incana		tree
Alnus japonica		tree
Alnus maximowiczii		tree
Alnus oregana		tree
Alnus rhombifolia		tree
Alnus rubra		tree
Alnus sp.		tree
Alnus viridis		tree
Betula albosinensis var. *septentrionalis*		tree
Betula alleghaniensis		tree
Betula dahurica		tree
Betula ermanii		tree
Betula lenta		tree
Betula lutea		tree
Betula maximowicziana		tree
Betula nigra		tree
Betula papyrifera		tree
Betula pendula		tree

Betula platyphylla	tree	
Betula platyphylla var. *japonica*	tree	
Betula platyphylla var. *szechuanica*	tree	
Betula pubescens	tree	
Betula pubescens ssp. *carpatica*	tree	
Betula sp.	tree	
Betula tortuosa	tree	
Carpinus betulus	tree	
Carpinus caroliniana	tree	
Carpinus cordata	tree	
Carpinus laxiflora	tree	
Carpinus tschonoskii	tree	
Carpinus viminea	tree	
Corylus sp.	tree	
Ostrya carpinifolia	tree	
Ostrya japonica	tree	
Ostrya virginiana	tree	
**BIGNONIACEAE**		
Amphitecna tuxtlensis	tree	
Chilopsis linearis	tree	
Crescentia alata	tree	
Crescentia cujete	tree	
Distictis granulosa	tree	
Godmania aesculifolia	tree	
Godmania macrocarpa	tree	
Jacaranda copaia	tree	
Kigelia pinnata	tree	
Markhamia platycalyx	tree	
Radermachera sinica	tree	
Spathodea campanulata	tree	
Tabebuia chrysantha	tree	
Tabebuia haemantha	tree	
Tabebuia heterophylla	tree	
Tabebuia impetiginosa	tree	
Tabebuia ochracea	tree	
Tabebuia pallida	tree	
Tabebuia rigida	tree	
Tabebuia rosea	tree	
Tabebuia sp.	tree	
Arrabidaea verrucosa		liana
Campsis radicans		liana
Mansoa verrucifera		liana
Memora sp		liana
Memora sp.		liana

Phryganocydia corymbosa			liana
Pleonotoma variabilis			liana
**BIXACEAE**			
Cochlospermum vitifolium		tree	
**BORAGINACEAE**			
Cordia dentata	shrub		
Cortesia cuneifolia	shrub		
Eriodictyon californicum	shrub		
Heliotropium ramosissimum	shrub		
Onosma frutescens	shrub		
Beureria cumanensis		tree	
Bourreria succulenta		tree	
Cordia africana		tree	
Cordia alliodora		tree	
Cordia borinquensis		tree	
Cordia caffra		tree	
Cordia collococca		tree	
Cordia dichotoma		tree	
Cordia elaeagnoides		tree	
Cordia megalantha		tree	
Cordia panamensis		tree	
Cordia sp.		tree	
Cordia sprucei		tree	
Cordia ucayaliensis		tree	
Sloanea pulleniana		tree	
Tournefortia fuliginosa		tree	
**BRASSICACEAE**			
Alyssum spinosum	shrub		
Eremophila leptophylla	shrub		
Zilla spinosa	shrub		
**BRUNIACEAE**			
Berzelia lanuginosa	shrub		
**BURSERACEAE**			
Boswellia serrata		tree	
Bursera heteresthes		tree	
Bursera simaruba		tree	
Bursera simaruba		tree	
Bursera tomentosa		tree	
Canarium album		tree	
Commiphora pyracanthoides		tree	
Commiphora sp.		tree	
Crepidospermum cf. *goudotianum*		tree	
Crepidospermum goudotianum		tree	
Dacryodes cf. *sclerophylla*		tree	

Dacryodes excelsa		tree
Dacryodes nitens		tree
Dacryodes sp.		tree
Protium cf. *aracouchini*		tree
Protium cf. *carnosum*		tree
Protium cf. *insigne*		tree
Protium cf. *neglectum* var. *robustum*		tree
Protium cf. *paniculatum* var. *paniculatum*		tree
Protium cf. *robustum*		tree
Protium krukoffii		tree
Protium opacum		tree
Protium pallidum		tree
Protium spp.		tree
Protium subserratum		tree
Protium tenuifolium		tree
Tetragastris balsamifera		tree
Tetragastris cf. *altissima*		tree
Tetragastris panamensis		tree
Trattinnickia aspera		tree
Trattinnickia rhoifolia		tree
Trattinnickia sp.		tree
**BUXACEAE**		
Buxus sempervirens	shrub	
Sarcococca ruscifolia	shrub	
Buxus macowanii		tree
**CALOPHYLLACEAE**		
Calophyllum inophyllum		tree
Calophyllum longifolium		tree
Calophyllum sp.		tree
Caraipa heterocarpa		tree
Kielmeyera coriacea		tree
Mammea africana		tree
Marila laxiflora		tree
Calophyllum brasiliense		
**CAMPANULACEAE**		
Brighamia insignis	shrub	
Clermontia fauriei	shrub	
Clermontia montis-loa	shrub	
Clermontia parviflora	shrub	
Cyanea floribunda	shrub	
Cyanea hirtella	shrub	
Cyanea leptostegia	shrub	
Cyanea pilosa	shrub	
Delissea rhytidosperma	shrub	

Lobelia villosa	shrub	
Lobelia yuccoides	shrub	
Trematolobelia kauaiensis	shrub	
**CANNABACEAE**		
Trema cannabina	shrub	
Celtis adolfi-friderici		tree
Celtis africana		tree
Celtis australis		tree
Celtis durandii		tree
Celtis occidentalis		tree
Celtis pallida		tree
Celtis sinensis		tree
Celtis tala		tree
Celtis tetrandra ssp. *sinensis*		tree
Gironniera subaequalis		tree
Pteroceltis tatarinowii		tree
Trema micrantha		tree
Trema orientalis		tree
Trema tomentosa		tree
**CAPPARACEAE**		
Capparis verrucosa	shrub	tree
Capparis spinosa	shrub	
Boscia albitrunca		tree
Capparis aristiguetae		tree
Capparis atamisquea		tree
Capparis baducca		tree
Capparis cynophallophora		tree
Capparis flexuosa		tree
Capparis indica		tree
Capparis linearis		tree
Capparis odoratissima		tree
Capparis pachaca		tree
Cladostemon kirkii		tree
Morisonia americana		tree
**CAPRIFOLIACEAE**		
Cephalaria squamiflora	shrub	
Diervilla lonicera	shrub	
Linnaea borealis	shrub	
Lonicera baltica	shrub	
Lonicera bella	shrub	
Lonicera canadensis	shrub	
Lonicera chrysantha	shrub	
Lonicera hispida	shrub	
Lonicera koehneana	shrub	

Lonicera maximowiczii	shrub		
Lonicera pyrenaica	shrub		
Lonicera ruprechtiana	shrub		
Lonicera semenovii	shrub		
Lonicera sp.	shrub		
Lonicera thibetica	shrub		
Lonicera xylosteum	shrub		
Symphoricarpos albus	shrub		
Symphoricarpos orbiculatus	shrub		
Lonicera affinis			liana
Lonicera dioica			liana
Lonicera etrusca			liana
Lonicera hirsuta			liana
Lonicera hispidula			liana
Lonicera implexa			liana
Lonicera japonica			liana
Lonicera periclymenum			liana
**CARDIOPTERIDACEAE**			
Dendrobangia boliviana		tree	
Leptaulus daphnoides		tree	
**CARICACEAE**			
Jacaratia spinosa		tree	
**CARYOCARACEAE**			
Caryocar brasiliense		tree	
Caryocar glabrum		tree	
**CARYOPHYLLACEAE**			
Silene mollissima	shrub		
**CASUARINACEAE**			
Allocasuarina humilis	shrub		
Allocasuarina pusilla		tree	
Allocasuarina sp		tree	
Casuarina equisetifolia		tree	
Casuarina humilis		tree	
Casuarina spp.		tree	
**CELASTRACEAE**			
Maytenus oleoides	shrub	tree	
Euonymus alatus	shrub		
Euonymus myrianthus	shrub		
Euonymus sp.	shrub		
Euonymus verrucosus	shrub		
Maytenus boaria	shrub		
Maytenus canariensis	shrub		
Schaefferia cuneifolia	shrub		
Cassine xylocarpa		tree	

Euonymus bungeanus		tree	
Euonymus europaeus		tree	
Hartogiella schinoides		tree	
Hemiangium excelsum		tree	
Maytenus acuminata		tree	
Maytenus magellanica		tree	
Maytenus vitis-idaea		tree	
Salacia leptoclada		tree	
Celastrus hypoleucus			liana
Celastrus orbiculatus			liana
Euonymus fortunei			liana
Hippocratea volubilis			liana
Salacia megistophylla			liana
Tontelea richardii			liana
Tropterygium forrestii			liana
**CERCIDIPHYLLACEAE**			
Cercidiphyllum japonicum		tree	
**CHLORANTHACEAE**			
Hedyosmum arborescens		tree	
Hedyosmum mexicanum		tree	
**CHRYSOBALANACEAE**			
Acioa barteri	shrub		
Chrysobalanus icaco	shrub		
Acioa guianensis		tree	
Acioa staudtii		tree	
Couepia caryophylloides		tree	
Couepia guianensis		tree	
Hirtella punctillata		tree	
Hirtella triandra		tree	
Licania cf. *hispidula*		tree	
Licania alba		tree	
Licania arborea		tree	
Licania glabriflora		tree	
Licania granvillei		tree	
Licania heteromorpha		tree	
Licania kunthiana		tree	
Licania lata		tree	
Licania latifolia		tree	
Licania macrophylla		tree	
Licania membranacea		tree	
Licania micrantha		tree	
Licania ovalifolia		tree	
Licania sp.		tree	
Parinari excelsa		tree	

Parinari montana		tree
Parinari sp.		tree
**CISTACEAE**		
Cistus albidus	shrub	
Cistus clusii	shrub	
Cistus crispus	shrub	
Cistus incanus	shrub	
Cistus ladanifer	shrub	
Cistus ladaniferus	shrub	
Cistus laurifolius	shrub	
Cistus libanotis	shrub	
Cistus monspeliensis	shrub	
Cistus parviflorus	shrub	
Cistus populifolius	shrub	
Cistus salviifolius	shrub	
Cistus salviifolius	shrub	
Cistus simphytifolius	shrub	
Cistus varius	shrub	
Halimium commutatum	shrub	
Halimium halimifolium	shrub	
Halimium umbellatum	shrub	
Helianthemum nummularium	shrub	
Helianthemum squamatum	shrub	
**CLEOMACEAE**		
Cleome droserifolia	shrub	
**CLETHRACEAE**		
Clethra alnifolia	shrub	
Clethra barbinervis	shrub	
Clethra mexicana		tree
Clethra occidentalis		tree
Purdiaea nutans		tree
**CLUSIACEAE**		
Clusia fluminensis	shrub	
Clusia hilariana	shrub	
Clusia parviflora	shrub	
Garcinia paucinervis	shrub	
Clusia cf. *havetioides*		tree
Clusia elliptica		tree
Clusia multiflora		tree
Clusia rosea		tree
Clusia spp.		tree
Garcinia spp.		tree
Garcinia bancana		tree
Garcinia cowa		tree

Garcinia intermedia		tree	
Garcinia malaccensis		tree	
Garcinia mannii		tree	
Garcinia nervosa		tree	
Garcinia ovalifolia		tree	
Garcinia scortechinii		tree	
Garcinia sp.		tree	
Garcinia tinctoria		tree	
Moronobea coccinea		tree	
Platonia insignis		tree	
Rheedia edulis		tree	
Rheedia sp.		tree	
Symphonia globulifera		tree	
Symphonia sp.		tree	
Tovomita sp.		tree	
**COCHLOSPERMACEAE**			
Cochlospermum fraseri		tree	
**COMBRETACEAE**			
Combretum micranthum	shrub		
Guiera senegalensis	shrub		
Lumnitzera racemosa	shrub		
Quisqualis indica	shrub		
Anogeissus latifolia		tree	
Bucida buceras		tree	
Combretum apiculatum		tree	
Combretum nigricans var. *elliotii*		tree	
Laguncularia racemosa		tree	
Lumnitzera littorea		tree	
Pteleopsis myrtifolia		tree	
Terminalia alata		tree	
Terminalia arjuna		tree	
Terminalia arostrata		tree	
Terminalia bellirica		tree	
Terminalia canescens		tree	
Terminalia catappa		tree	
Terminalia chebula		tree	
Terminalia ferdinandiana		tree	
Terminalia hainanensis		tree	
Terminalia microcarpa		tree	
Terminalia oblonga		tree	
Terminalia sericea		tree	
Terminalia superba		tree	
Combretum fruticosum			liana
**CONNARACEAE**			

Connarus perrottetii var. *angustifolius*		tree	
Connarus suberosus		tree	
Agelaea pentagyna			liana
Agelaea poggeana			liana
**CONVOLVULACEAE**			
Ipomoea wolcottiana		tree	
Jacquemontia nodiflora			liana
Maripa panamensis			liana
Merremia quinquefolia			liana
**CORIARIACEAE**			
Coriaria sinica	shrub		
Coriaria arborea		tree	
**CORNACEAE**			
Cornus alternifolia	shrub	tree	
Cornus sp.	shrub	tree	
Chamaepericlymenum suecicum	shrub		
Cornus kousa var. *chinensis*	shrub		
Cornus macrophylla	shrub		
Cornus mas	shrub		
Cornus officinalis	shrub		
Cornus racemosa	shrub		
Cornus rugosa	shrub		
Cornus sanguinea	shrub		
Cornus stolonifera	shrub		
Cornus suecica	shrub		
Swida sanguinea	shrub		
Alangium villosum		tree	
Cornus controversa		tree	
Cornus disciflora		tree	
Cornus florida		tree	
Cornus kousa		tree	
Dendrobenthamia capitata		tree	
Nyssa aquatica		tree	
Nyssa sylvatica		tree	
**COSTACEAE**			
Costus pulverulentus	shrub		
**CUCURBITACEAE**			
Albizia schimperiana		tree	
**CUCURBITACEAE**			
Cayaponia granatensis			liana
Dieterlea fusiformis			liana
Doyerea emetocathartica			liana
Trichosanthes miyagii			liana

**CUNONIACEAE**			
Pancheria elegans	shrub		
Caldcluvia brassii		tree	
Callicoma serratifolia		tree	
Ceratopetalum gummiferum		tree	
Codia discolor		tree	
Cunonia capensis		tree	
Eucryphia cordifolia		tree	
Weinmannia pinnata		tree	
Weinmannia racemosa		tree	
Weinmannia sp.		tree	
Weinmannia trichosperma		tree	
**CURTISIACEAE**			
Curtisia dentata		tree	
**CYRILLACEAE**			
Cyrilla racemiflora	shrub	tree	
Itea virginica	shrub		
**DAPHNIPHYLLACEAE**			
Daphniphyllum calycinum	shrub		
Daphniphyllum humile	shrub		
Daphniphyllum macropodum	shrub		
Daphniphyllum glaucescens		tree	
Daphniphyllum oldhamii		tree	
**DIAPENSIA**			
Diapensia lapponica	shrub		
**DICHAPETALACEAE**			
Tapura amazonica		tree	
**DILLENIACEAE**			
Hibbertia bracteata	shrub		
Hibbertia huegelii	shrub		
Hibbertia riparia	shrub		
Hibbertia sericea	shrub		
Hibbertia subvaginata	shrub		
Curatella americana		tree	
Davilla rugosa		tree	
Dillenia ferruginea		tree	
Dillenia indica		tree	
Dillenia suffruticosa		tree	
Hibbertia diffusa		tree	
Hibbertia hypericoides		tree	
Hibbertia sistiflora		tree	
Doliocarpus dentatus			liana
**DIPTEROCARPACEAE**			
Cotylelobium burckii		tree	

Dipterocarpus borneensis	tree
Dipterocarpus sublamellatus	tree
Dryobalanops aromatica	tree
Dryobalanops lanceolata	tree
Hopea hainanensis	tree
Hopea dryobalanoides	tree
Hopea pentanervia	tree
Monotes glaber	tree
Parashorea malaanonan	tree
Shorea argentifolia	tree
Shorea balanocarpoides	tree
Shorea faguetiana	tree
Shorea gibbosa	tree
Shorea javanica	tree
Shorea johorensis	tree
Shorea laxa	tree
Shorea leprosula	tree
Shorea macrophylla	tree
Shorea multiflora	tree
Shorea pachyphylla	tree
Shorea pauciflora	tree
Shorea pinanga	tree
Shorea quadrinervis	tree
Shorea robusta	tree
Vatica mangachapoi	tree
**EBENACEAE**	
Diospyros adenophora	tree
Diospyros apiculata	tree
Diospyros australis	tree
Diospyros cauliflora	tree
Diospyros dendo	tree
Diospyros embryopteris	tree
Diospyros hainanensis	tree
Diospyros howii	tree
Diospyros hoyleana	tree
Diospyros inflata	tree
Diospyros inhacaensis	tree
Diospyros kaki	tree
Diospyros latisepala	tree
Diospyros maingayi	tree
Diospyros melanoxylon	tree
Diospyros mollifolia	tree
Diospyros natalensis	tree
Diospyros nicaraguensis	tree

Diospyros nutans		tree	
Diospyros scortechinii		tree	
Diospyros subrotata		tree	
Diospyros texana		tree	
Diospyros thomasii		tree	
Diospyros whyteana		tree	
Euclea divinorum		tree	
**ELAEAGNACEAE**			
Hippophae rhamnoides	shrub	tree	
Elaeagnus glabra	shrub		liana
Elaeagnus pungens	shrub		
Hippophae tibetana	shrub		
Elaeagnus angustifolia		tree	
Elaeagnus oxycarpa		tree	
**ELAEOCARPACEAE**			
Aristotelia chilensis	shrub		
Aristotelia macqui	shrub		
Aristotelia serrata		tree	
Elaeocarpus apiculatus		tree	
Elaeocarpus decipiens		tree	
Elaeocarpus japonicus		tree	
Elaeocarpus ptilanthus		tree	
Elaeocarpus sayeri		tree	
Sloanea berteriana		tree	
Sloanea cf. *rufa*		tree	
Sloanea garckeana		tree	
Sloanea sp.		tree	
Sloanea terniflora		tree	
Crinodendron patagua	shrub		
**EMMOTACEAE**			
Apodytes dimidiata		tree	
**ERICACEAE**			
Arbutus menziesii	shrub	tree	
Kalmia latifolia	shrub	tree	
Rhododendron maximum	shrub	tree	
Rhododendron thymifolium	shrub	tree	
Andersonia heterophylla	shrub		
Andromeda glaucophylla	shrub		
Andromeda polifolia	shrub		
Arbutus andrachne	shrub		
Arctostaphylos alpina	shrub		
Arctostaphylos crustacea	shrub		
Arctostaphylos glandulosa	shrub		
Arctostaphylos glauca	shrub		

Arctostaphylos hookeri	shrub
Arctostaphylos montereyensis	shrub
Arctostaphylos nevadensis	shrub
Arctostaphylos pumila	shrub
Arctostaphylos pungens	shrub
Arctostaphylos tomentosa	shrub
Arctostaphylos uva-ursi	shrub
Arctostaphylos viscida	shrub
Astroloma xerophyllum	shrub
Brachyloma ericoides	shrub
Calluna sp.	shrub
Calluna vulgaris	shrub
Cassiope hypnoides	shrub
Cassiope tetragona	shrub
Chamaedaphne calyculata	shrub
Chimaphila umbellata	shrub
Conostephium pendulum	shrub
Dracophyllum prostratum	shrub
Empetrum hermaphroditum	shrub
Empetrum nigrum	shrub
Epigaea repens	shrub
Erica arborea	shrub
Erica ciliaris	shrub
Erica cinerea	shrub
Erica corifolia	shrub
Erica erigena	shrub
Erica herbacea	shrub
Erica mackaiana	shrub
Erica multiflora	shrub
Erica pulchella	shrub
Erica scoparia	shrub
Erica tetralix	shrub
Erica vagans	shrub
Gaultheria antipoda	shrub
Gaultheria crassa	shrub
Gaultheria hispidula	shrub
Gaultheria procumbens	shrub
Gaultheria shallon	shrub
Gaultheria yunnanensis	shrub
Gaylussacia baccata	shrub
Kalmia angustifolia	shrub
Kalmia polifolia	shrub
Ledum decumbens	shrub
Ledum groenlandicum	shrub

Ledum palustre	shrub
Leucopogon conostephioides	shrub
Leucopogon parviflorus	shrub
Leucothoe racemosa	shrub
Loiseleuria procumbens	shrub
Lyonia lucida	shrub
Lyonia octandra	shrub
Lyonia ovalifolia	shrub
Lyonia ovalifolia var. *elliptica*	shrub
Moneses uniflora	shrub
Phyllodoce caerulea	shrub
Pieris phillyreifolia	shrub
Rhododendron agglutinatum	shrub
Rhododendron anthopogon	shrub
Rhododendron arboreum	shrub
Rhododendron aureum	shrub
Rhododendron bachii	shrub
Rhododendron dauricum	shrub
Rhododendron decorum	shrub
Rhododendron fastigiatum	shrub
Rhododendron fortunei	shrub
Rhododendron hainanense	shrub
Rhododendron kwangtungense	shrub
Rhododendron lapponicum	shrub
Rhododendron mariae	shrub
Rhododendron micranthum	shrub
Rhododendron nigropunctatum	shrub
Rhododendron ponticum	shrub
Rhododendron przewalskii	shrub
Rhododendron rivulare	shrub
Rhododendron simsii	shrub
Rhododendron sp.	shrub
Rhododendron spiciferum	shrub
Rhododendron spinuliferum	shrub
Rhododendron superbum	shrub
Rhododendron vialii	shrub
Styphelia malayana	shrub
Styphelia tameiameiae	shrub
Vaccinium angustifolium	shrub
Vaccinium arboreum	shrub
Vaccinium bracteatum	shrub
Vaccinium calycinum	shrub
Vaccinium calycinum	shrub
Vaccinium consanguineum	shrub

Vaccinium corymbosum	shrub		
Vaccinium dunalianum	shrub		
Vaccinium floribundum	shrub		
Vaccinium laetum	shrub		
Vaccinium myrtilloides	shrub		
Vaccinium myrtillus	shrub		
Vaccinium ovatum	shrub		
Vaccinium oxycoccos	shrub		
Vaccinium reticulatum	shrub		
Vaccinium uliginosum	shrub		
Vaccinium vitis-idaea	shrub		
Zenobia pulverulenta	shrub		
Arbutus canariensis		tree	
Arbutus unedo		tree	
Befaria aestuans		tree	
Comarostaphylis arbutoides		tree	
Ericaceae sp.		tree	
Gaultheria foliolosa		tree	
Oxydendrum arboreum		tree	
Rhododendron maximum		tree	
Rhododendron ovatum		tree	
Vaccinium mandarinorum		tree	
Vaccinium meridionale		tree	
Vaccinium sp.		tree	
Agapetes meiniana			liana
Orthaea sp.			liana
**ERYTHROXYLACEAE**			
Erythroxylum areolatum		tree	
Erythroxylum cumanense		tree	
Erythroxylum densum		tree	
Erythroxylum orinocense		tree	
Erythroxylum rotundifolium		tree	
**ESCALLONIACEAE**			
Anopterus glandulosus	shrub		
Escallonia myrtilloides		tree	
**EUPHORBIACEAE**			
Acalypha skutchii	shrub		
Adelia triloba	shrub		
Alchornea hirtella	shrub		
Alchornea rugosa	shrub		
Alchornea trewioides	shrub		
Bertya cunninghamii	shrub		
Beyeria opaca	shrub		
Coelodepas hainanensis	shrub		

Colliguaja integerrima	shrub	
Colliguaja odorifera	shrub	
Colliguaja salicifolia	shrub	
Croton kwangsiensis	shrub	
Euphorbia acanthothamnos	shrub	
Euphorbia characias	shrub	
Euphorbia margalidiana	shrub	
Euphorbia pithyusa	shrub	
Jatropha gossypiifolia	shrub	
Mallotus anomalus	shrub	
Mallotus apelta	shrub	
Phyllanthus gasstroemii	shrub	
Sebastiania pavoniana	shrub	
Acalypha caturus		tree
Acalypha cf. *caturus*		tree
Acalypha communis		tree
Adelia oaxacana		tree
Alchornea brachygyne		tree
Alchornea castaneifolia		tree
Alchornea cordifolia		tree
Alchornea costaricensis		tree
Alchornea costaricensis		tree
Alchornea floribunda		tree
Alchornea glandulosa		tree
Alchornea grandis		tree
Alchornea iricurana		tree
Alchornea latifolia		tree
Alchornea triplinervia		tree
Alchorneopsis portoricensis		tree
Alchorneopsis trimera		tree
Aleurites moluccana		tree
Chaetocarpus globosus		tree
Chaetocarpus schomburgkia		tree
Claoxylon indicum		tree
Croton billbergianus		tree
Croton draco		tree
Croton matourensis		tree
Croton poecilanthus		tree
Croton pseudoniveus		tree
Croton sarcopetalus		tree
Croton schiedeanus		tree
Croton steenkampianus		tree
Dichostemma caloneura		tree
Dichostemma glaucens		tree

Ditta myricoides	tree	
Dodecastigma integrifolium	tree	
Elateriospermum tapos	tree	
Euphorbia pyrifolia	tree	
Excoecaria agallocha	tree	
Glycydendron amazonicum	tree	
Hevea cf. *brasiliensis*	tree	
Hevea brasiliensis	tree	
Hevea guianensis	tree	
Homalanthus populneus	tree	
Hura crepitans	tree	
Jatropha standleyi	tree	
Mabea speciosa	tree	
Macaranga beccariana	tree	
Macaranga gigantea	tree	
Macaranga heynei	tree	
Macaranga hispida	tree	
Macaranga hosei	tree	
Macaranga hullettii	tree	
Macaranga hypoleuca	tree	
Macaranga lamellata	tree	
Macaranga lowii	tree	
Macaranga sp	tree	
Macaranga tanarius	tree	
Macaranga trachyphylla	tree	
Macaranga triloba	tree	
Macaranga winkleri	tree	
Mallotus barbatus	tree	
Mallotus nesophilus	tree	
Mallotus paniculatus	tree	
Mallotus philippensis	tree	
Manihot esculenta	tree	
Micrandra sprucei	tree	
Omphalea oleifera	tree	
Ostodes paniculata	tree	
Pera sp.	tree	
Ptychopyxis caput-medusae	tree	
Sapium aucuparium	tree	
Sapium discolor	tree	
Sapium glandulosum	tree	
Sapium rotundifolium	tree	
Sapium sebiferum	tree	
Suregada zanzibariensis	tree	
Dalechampia scandens		liana

Euphorbia colletioides			liana
Manihot chlorosticta			liana
**FABACEAE**			
Acacia colletioides	shrub	tree	
Acacia doratoxylon	shrub	tree	
Acacia havilandiorum	shrub	tree	
Acacia oswaldii	shrub	tree	
Anthonotha macrophylla	shrub	tree	
Inga punctata	shrub	tree	
Inga thibaudiana	shrub	tree	
Pultenaea daphnoides	shrub	tree	
Pultenaea flexilis	shrub	tree	
Acacia angustissima	shrub		
Acacia bonariensis	shrub		
Acacia dimidiata	shrub		
Acacia farnesiana	shrub		
Acacia furcatispina	shrub		
Acacia greggii	shrub		
Acacia kempeana	shrub		
Acacia pruinocarpa	shrub		
Acacia rigidula	shrub		
Acacia tetragonophylla	shrub		
Acacia truncata	shrub		
Acacia wilhelmiana	shrub		
Adenocarpus foliolosus	shrub		
Adesmia spinosissima	shrub		
Aganope impressa	shrub		
Alhagi maurorum	shrub		
Ammopiptanthus mongolicus	shrub		
Amorpha canescens	shrub		
Amorpha fruticosa	shrub		
Amorpha sp.	shrub		
Anagyris foetida	shrub		
Anthyllis hermanniae	shrub		
Astragalus adsurgens	shrub		
Astragalus canadensis	shrub		
Astragalus spinosus	shrub		
Astragalus tibetanus	shrub		
Astrotricha floccosa	shrub		
Bossiaea eriocarpa	shrub		
Bossiaea walkeri	shrub		
Calicotome spinosa	shrub		
Calicotome villosa	shrub		
Caragana davazamcii	shrub		

Caragana intermedia	shrub
Caragana jubata	shrub
Caragana korshinskii	shrub
Caragana microphylla	shrub
Caragana pygmaea	shrub
Caragana roborovskyi	shrub
Caragana sp.	shrub
Caragana stenophylla	shrub
Caragana tibetica	shrub
Carmichaelia odorata	shrub
Cassia sturtii	shrub
Cercidium floridum	shrub
Cercis canadensis	shrub
Cercis siliquastrum	shrub
Chamaecrista bahiae	shrub
Chamaecytisus proliferus	shrub
Coursetia arborea	shrub
Crotalaria cunninghamii	shrub
Cytisus scoparius	shrub
Dalea fremontii	shrub
Daviesia incrassata	shrub
Daviesia preissii	shrub
Daviesia quadrilatera	shrub
Dorycnium hirsutum	shrub
Dorycnium spectabile	shrub
Eutaxia microphylla	shrub
Genista acanthoclada	shrub
Genista anglica	shrub
Genista pilosa	shrub
Genista pulchella	shrub
Genista tinctoria	shrub
Gleditsia heterophylla	shrub
Gompholobium glabratum	shrub
Gompholobium grandiflorum	shrub
Gompholobium latifolium	shrub
Hedysarum fruticosum	shrub
Hedysarum scoparium	shrub
Hedysarum tanguticum	shrub
Indigofera brevidens	shrub
Inga dumosa	shrub
Inga edulis	shrub
Inga lomatophylla	shrub
Inga macrophylla	shrub
Inga pilosula	shrub

Jacksonia floribunda	shrub	
Jacksonia furcellata	shrub	
Lespedeza bicolor	shrub	
Lespedeza buergeri	shrub	
Lespedeza davurica	shrub	
Lespedeza juncea	shrub	
Lotus scoparius	shrub	
Lupinus oreophilus	shrub	
Medicago arborea	shrub	
Oxytropis aciphylla	shrub	
Phyllota phylicoides	shrub	
Phyllota remota	shrub	
Pickeringia montana	shrub	
Prosopis glandulosa	shrub	
Senna alexandrina	shrub	
Senna aphylla	shrub	
Senna artemisioides	shrub	
Senna cumingii var. *eremobia*	shrub	
Senna quinquangulata	shrub	
Sophora alopecuroides	shrub	
Sophora flavescens	shrub	
Sophora moorcroftiana	shrub	
Sophora viciifolia	shrub	
Swainsona salsula	shrub	
Ulex europaeus	shrub	
Ulex gallii	shrub	
Ulex parviflorus	shrub	
Abarema curvicarpa		tree
Abarema jupunba		tree
Abarema jupunba var. *jupunba*		tree
Abarema macradenia		tree
Abarema mataybifolia		tree
Acacia aroma		tree
Acacia auriculiformis		tree
Acacia binervata		tree
Acacia brachybotrya		tree
Acacia caven		tree
Acacia cochliacantha		tree
Acacia collinsii		tree
Acacia crassicarpa		tree
Acacia floribunda		tree
Acacia gourmaensis		tree
Acacia hindsii		tree
Acacia holosericea		tree

Acacia karroo	tree
Acacia koa	tree
Acacia luederitzii	tree
Acacia macracantha	tree
Acacia mangium	tree
Acacia mayana	tree
Acacia mearnsii	tree
Acacia melanoxylon	tree
Acacia mimula	tree
Acacia nigrescens	tree
Acacia rigens	tree
Acacia robusta	tree
Acacia senegal	tree
Acacia stricta	tree
Acacia suaveolens	tree
Acacia tortilis	tree
Acacia visco	tree
Acosmium nitens	tree
Acosmium panamense	tree
Acosmium panamense	tree
Acosmium praeclarum	tree
Acrocarpus fraxinifolius	tree
Adenanthera pavonina	tree
Afzelia africana	tree
Afzelia bella	tree
Afzelia pachyloba	tree
Albizia adianthifolia	tree
Albizia antunesiana	tree
Albizia chinensis	tree
Albizia falcataria	tree
Albizia ferruginea	tree
Albizia guachapele	tree
Albizia gummifera	tree
Albizia inundata	tree
Albizia julibrissin	tree
Albizia laurentii	tree
Albizia lebbeck	tree
Albizia niopoides	tree
Albizia odoratissima	tree
Albizia pistaciifolia	tree
Albizia procera	tree
Albizia purpusii	tree
Alexa wachenheimii	tree
Amphimas pterocarpoides	tree

Andira coriacea	tree
Andira fraxinifolia	tree
Andira inermis	tree
Andira parviflora	tree
Anthonotha graciliflora	tree
Apuleia molaris	tree
Balizia pedicellaris	tree
Bauhinia forficata	tree
Bauhinia forficata	tree
Bauhinia holophylla	tree
Bauhinia megalandra	tree
Bauhinia racemosa	tree
Bauhinia sp.	tree
Bauhinia ungulata	tree
Bauhinia variegata	tree
Berlinia auriculata	tree
Bocoa prouacensis	tree
Bolusanthus speciosus	tree
Bowdichia virgilioides	tree
Brachystegia spiciformis	tree
Burkea africana	tree
Butea monosperma	tree
Caesalpinia eriostachys	tree
Caesalpinia granadillo	tree
Calliandra calothyrsus	tree
Cassia calothyrsus	tree
Cassia fistula	tree
Cassia grandis	tree
Cassia siamea	tree
Cassia spectabilis	tree
Cassia spruceana	tree
Cassia tora	tree
Centrolobium microchaete	tree
Chamaecytisus palmensis	tree
Cladrastis wilsonii	tree
Colophospermum mopane	tree
Copaifera multijuga	tree
Copaifera palustris	tree
Copaifera sp.	tree
Copaifera venezuelana	tree
Crudia aromatica	tree
Crudia bracteata	tree
Cynometra leonensis	tree
Cynometra retusa	tree

Dalbergia hainanensis	tree
Dalbergia melanoxylon	tree
Dalbergia odorifera	tree
Dalbergia retusa	tree
Dalbergia sissoo	tree
Daviesia nudiflora	tree
Dialium guianense	tree
Dialium guineense	tree
Dialium schlechteri	tree
Dicorynia guianensis	tree
Dinizia excelsa	tree
Diplotropis purpurea	tree
Dipteryx odorata	tree
Dussia mexicana	tree
Dussia munda	tree
Enterolobium cyclocarpum	tree
Eperua falcata	tree
Eperua grandiflora	tree
Eperua leucantha	tree
Eperua purpurea	tree
Erythrina berteroana	tree
Erythrina caffra	tree
Erythrina excelsa	tree
Erythrina folkersii	tree
Erythrina fusca	tree
Erythrina poeppigiana	tree
Erythrina variegata	tree
Erythrophleum chlorostachys	tree
Erythrophleum lasianthum	tree
Eysenhardtia texana	tree
Fissicalyx fendleri	tree
Flemingia congesta	tree
Fordia splendidissima	tree
Geoffroea decorticans	tree
Gleditsia triacanthos	tree
Gliricidia sepium	tree
Hardwickia binata	tree
Humboldtiella arborea	tree
Hymenaea courbaril	tree
Hymenaea parvifolia	tree
Hymenaea sp.	tree
Hymenolobium cf. *flavum*	tree
Inga acrocephala	tree
Inga alba	tree

Inga auristellae	tree
Inga cayennensis	tree
Inga cf. *alba*	tree
Inga cf. *capitata*	tree
Inga cf. *semialata*	tree
Inga cinnamomea	tree
Inga densiflora	tree
Inga fanchoniana	tree
Inga flagelliformis	tree
Inga gracilifolia	tree
Inga huberi	tree
Inga laurina	tree
Inga leiocalycina	tree
Inga paraensis	tree
Inga pezizifera	tree
Inga quaternata	tree
Inga rubiginosa	tree
Inga sarmentosa	tree
Inga splendens	tree
Inga spp.	tree
Inga stipularis	tree
Inga tubiformis	tree
Inga vera	tree
Julbernardia globiflora	tree
Laburnum anagyroides	tree
Leucaena leucocephala	tree
Librevillea klainei	tree
Lonchocarpus dipteroneurus	tree
Lonchocarpus eriocarinalis	tree
Lonchocarpus felipei	tree
Lonchocarpus guatemalensis	tree
Lonchocarpus latifolius	tree
Lonchocarpus minimiflorus	tree
Lonchocarpus phlebophyllus	tree
Lonchocarpus rugosus	tree
Lysiloma divaricatum	tree
Lysiphyllum cunninghamii	tree
Maackia amurensis	tree
Machaerium biovulatum	tree
Machaerium sp.	tree
Martiodendron sp.	tree
Millettia dura	tree
Millettia ferruginea	tree
Millettia leptobotrya	tree

Mimozyganthus carinatus	tree
Newtonia hildebrandtii	tree
Ormosia balansae	tree
Ormosia krugii	tree
Ormosia melanocarpa	tree
Ormosia pinnata	tree
Ormosia sp.	tree
Paramachaerium ormosioides	tree
Parkia cf. *multijuga*	tree
Parkia cf. *nitida*	tree
Parkia multijuga	tree
Parkia nitida	tree
Parkia spp	tree
Peltogyne cf. *excelsa*	tree
Peltogyne cf. *heterophylla*	tree
Peltogyne cf. *venosa* ssp. *densiflora*	tree
Peltogyne prancei	tree
Peltogyne sp.	tree
Peltogyne venosa	tree
Pentaclethra macroloba	tree
Pentaclethra macrophylla	tree
Pictetia aculeata	tree
Piptadeniastrum africanum	tree
Piscidia carthagenensis	tree
Pithecellobium caraboboense	tree
Pithecellobium cauliflorum	tree
Pithecellobium dulce	tree
Pithecellobium jupunba	tree
Pithecellobium ligustrinum	tree
Pithecellobium racemosum	tree
Pithecellobium saman	tree
Plagiosiphon emarginatus	tree
Platymiscium pinnatum	tree
Poecilanthe hostmannii	tree
Poeppigia procera	tree
Pongamia pinnata	tree
Prioria copaifera	tree
Prosopis chilensis	tree
Prosopis flexuosa	tree
Prosopis juliflora	tree
Pseudopiptadenia suaveolens	tree
Pterocarpus marsupium	tree
Pterocarpus officinalis	tree
Pterocarpus rohrii	tree

Pultenaea elliptica	tree	
Pultenaea myrtoides	tree	
Pultenaea tenuifolia	tree	
Recordoxylon venosa	tree	
Robinia pseudoacacia	tree	
Robinia pseudoacacia var. *intermis*	tree	
Samanea saman	tree	
Saraca indica	tree	
Schizolobium excelsum	tree	
Sclerolobium cf. *setiferum*	tree	
Senna atomaria	tree	
Sesbania sesban	tree	
Sophora chrysophylla	tree	
Sophora japonica	tree	
Sophora sp.	tree	
Stryphnodendron polystachyum	tree	
Stryphnodendron pulcherrimum	tree	
Swartzia arborescens	tree	
Swartzia cf. *ingifolia*	tree	
Swartzia guianensis	tree	
Swartzia panacoco	tree	
Swartzia polyphylla	tree	
Swartzia simplex	tree	
Sweetia fruticosa	tree	
Tachigali albiflora	tree	
Tachigali cavipes	tree	
Tachigali cf. *chrysophylla*	tree	
Tachigali cf. *rugosa*	tree	
Tachigali guianensis	tree	
Tachigali melinonii	tree	
Tachigali paniculata	tree	
Tachigali paraensis	tree	
Tachigali sp.	tree	
Tachigali versicolor	tree	
Umtiza listeriana	tree	
Vatairea lundellii	tree	
Vouacapoua americana	tree	
Zygia racemosa	tree	
Fabaceae sp.	tree	
Abrus canescens		liana
Acacia tucumanensis		liana
Bauhinia championii		liana
Bauhinia tenuiflora		liana
Dalbergia armata		liana

Entadopsis polystachya			liana
Galactia acapulcensis			liana
Millettia pachycarpa			liana
Mimosa albida			liana
Mimosa micheliana			liana
Mucuna macrocarpa			liana
Nissolia leiogyne			liana
Pueraria lobata			liana
Wisteria floribunda			liana
**FAGACEAE**			
Castanea seguinii	shrub	tree	
Quercus agrifolia	shrub	tree	
Quercus ilex	shrub	tree	
Quercus turbinella	shrub	tree	
Castanopsis chrysophylla	shrub		
Quercus coccifera	shrub		
Quercus dumosa	shrub		
Quercus durata	shrub		
Quercus lusitanica	shrub		
Castanea dentata		tree	
Castanea henryi		tree	
Castanea mollissima		tree	
Castanea sativa		tree	
Castanopsis acuminatissima		tree	
Castanopsis carlesii		tree	
Castanopsis carlesii var. *hainanca*		tree	
Castanopsis ceratacantha		tree	
Castanopsis delavayi		tree	
Castanopsis echinocarpa		tree	
Castanopsis eyrei		tree	
Castanopsis eyrei var. *caudata*		tree	
Castanopsis faberi		tree	
Castanopsis fargesii		tree	
Castanopsis fissa		tree	
Castanopsis formosana		tree	
Castanopsis hystrix		tree	
Castanopsis orthacantha		tree	
Castanopsis sclerophylla		tree	
Castanopsis sieboldii		tree	
Castanopsis sp.		tree	
Castanopsis stellatospina		tree	
Castanopsis tibetana		tree	
Castanopsis wattii		tree	
Cyclobalanopsis championii		tree	

Cyclobalanopsis gilva	tree
Cyclobalanopsis glauca	tree
Cyclobalanopsis glaucoides	tree
Cyclobalanopsis gracilis	tree
Cyclobalanopsis longinux	tree
Cyclobalanopsis myrsinifolia	tree
Cyclobalanopsis nubium	tree
Cyclobalanopsis oxyodon	tree
Cyclobalanopsis tiaoloshanica	tree
Fagus grandifolia	tree
Fagus japonica	tree
Fagus longipetiolata	tree
Fagus sylvatica	tree
Lithocarpus amygdalifolius	tree
Lithocarpus calophyllus	tree
Lithocarpus chintungensis	tree
Lithocarpus corneus	tree
Lithocarpus cyrtocarpus	tree
Lithocarpus dealbatus	tree
Lithocarpus fenzelianus	tree
Lithocarpus glaber	tree
Lithocarpus hancei	tree
Lithocarpus handelianus	tree
Lithocarpus harlandii	tree
Lithocarpus havilandii	tree
Lithocarpus xylocarpus	tree
Quercus acuta	tree
Quercus acutissima	tree
Quercus alba	tree
Quercus aliena var. *acutiserrata*	tree
Quercus austrina	tree
Quercus bicolor	tree
Quercus borealis	tree
Quercus castaneifolia	tree
Quercus cerris	tree
Quercus chapmanii	tree
Quercus chrysolepis	tree
Quercus coccinea	tree
Quercus copeyensis	tree
Quercus costaricensis	tree
Quercus crispula	tree
Quercus dentata	tree
Quercus douglasii	tree
Quercus ellipsoidalis	tree

Quercus fabrei	tree
Quercus faginea	tree
Quercus falcata	tree
Quercus floribunda	tree
Quercus geminata	tree
Quercus hemisphaerica	tree
Quercus humilis	tree
Quercus incana	tree
Quercus kelloggii	tree
Quercus laevis	tree
Quercus laurifolia	tree
Quercus leucotrichophora	tree
Quercus liaotungensis	tree
Quercus lobata	tree
Quercus macrocarpa	tree
Quercus margarettiae	tree
Quercus michauxii	tree
Quercus minima	tree
Quercus mongolica	tree
Quercus mongolica var. *grosseserrata*	tree
Quercus myrsinifolia	tree
Quercus myrtifolia	tree
Quercus nigra	tree
Quercus nuttallii	tree
Quercus oleoides	tree
Quercus patelliformis	tree
Quercus petraea	tree
Quercus prinus	tree
Quercus pubescens	tree
Quercus pumila	tree
Quercus pyrenaica	tree
Quercus robur	tree
Quercus rotundifolia	tree
Quercus rubra	tree
Quercus salicina	tree
Quercus shumardii	tree
Quercus sp.	tree
Quercus stellata	tree
Quercus suber	tree
Quercus tarokoensis	tree
Quercus vestita	tree
Quercus virginiana	tree
Quercus virginiana var. *geminata*	tree
Quercus xalapensis	tree

Trigonobalanus verticillata		tree
**FOUQUIERIACEAE**		
Fouquieria splendens	shrub	
**GARRYACEAE**		
Garrya laurifolia	shrub	
Aucuba chinensis		tree
**GENTIANACEAE**		
Macrocarpaea sodiroana	shrub	
**GOODENIACEAE**		
Goodenia ovata	shrub	
Scaevola taccada	shrub	
Verreauxia reinwardtii	shrub	
**GOUPIACEAE**		
Goupia glabra		tree
**GRISELINIACEAE**		
Griselinia littoralis		tree
**GROSSULARIACEAE**		
Grossularia reclinata	shrub	
Ribes alpinum	shrub	
Ribes californicum	shrub	
Ribes cynosbati	shrub	
Ribes diacanthum	shrub	
Ribes himalense	shrub	
Ribes hirtellum	shrub	
Ribes malvaceum	shrub	
Ribes nigrum	shrub	
Ribes rubrum	shrub	
Ribes spicatum	shrub	
Ribes uva-crispa	shrub	
**GYROSTEMONACEAE**		
Gyrostemon ramulosus	shrub	
Tersonia brevipes	shrub	
**HAMAMELIDACEAE**		
Loropetalum chinense	shrub	
Mytilaria laosensis		tree
Trichocladus crinitus		tree
Trichocladus ellipticus		tree
**HELICONIACEAE**		
Heliconia pogonantha		tree
**HERNANDIACEAE**		
Hernandia bivalvis		tree
Hernandia sonora		tree
**HUACEAE**		
Afrostyrax lepidophyllus		tree

**HUMIRIACEAE**			
Endopleura sp.		tree	
Endopleura uchi		tree	
Humiria balsamifera		tree	
Humiriastrum diguense		tree	
Humiriastrum subcrenatum		tree	
Vantanea parviflora		tree	
**HYDRANGEACEAE**			
Broussaisia arguta	shrub		
Deutzia scabra	shrub		
Hydrangea paniculata	shrub		
Philadelphus incanus	shrub		
Hydrangea petiolaris			liana
Pileostegia viburnoides			liana
**HYPERICACEAE**			
Cratoxylon ligustrinum	shrub	tree	
Hypericum balearicum	shrub		
Hypericum hircinum var. *cambessedesei*	shrub		
Hypericum reflexum	shrub		
Vismia guianensis		tree	
Vismia japurensis		tree	
Vismia lauriformis		tree	
**ICACINACEAE**			
Poraqueiba guianensis		tree	
**ITEACEAE**			
Itea chinensis	shrub		
**IXONANTHACEAE**			
Ixonanthes reticulata		tree	
**JUGLANDACEAE**			
Carya cordiformis		tree	
Carya glabra		tree	
Carya ovata		tree	
Carya sp.		tree	
Carya tomentosa		tree	
Engelhardtia chrysolepis		tree	
Juglans ailanthifolia		tree	
Juglans cinerea		tree	
Juglans mandshurica		tree	
Juglans nigra		tree	
Juglans regia		tree	
Juglans sp.		tree	
Platycarya strobilacea		tree	
**KRAMERIACEAE**			

Krameria grayi	shrub	
Krameria parvifolia	shrub	
**LACISTEMATACEAE**		
Lacistema pubescens		tree
Lozania pittieri		tree
**LAMIACEAE**		
Acinos alpinus	shrub	
Acinos arvensis	shrub	
Aegiphila integrifolia	shrub	
Aegiphila intermedia	shrub	
Aegiphila lhotzkiana	shrub	
Ballota acetabulosa	shrub	
Calamintha nepeta	shrub	
Calamintha nepeta	shrub	
Clerodendron infortunatum	shrub	
Clerodendrum cyrtophyllum	shrub	
Lavandula dentata	shrub	
Lavandula latifolia	shrub	
Lavandula stoechas	shrub	
Lepechinia calycina	shrub	
Micromeria nervosa	shrub	
Phlomis fruticosa	shrub	
Phlomis italica	shrub	
Phlomis purpurea	shrub	
Pityrodia bartlingii	shrub	
Prasium majus	shrub	
Premna serratifolia	shrub	
Rosmarinus officinalis	shrub	
Salvia deserta	shrub	
Salvia dorrii var. *dorrii*	shrub	
Salvia leucophylla	shrub	
Salvia mellifera	shrub	
Salvia mohavensis	shrub	
Satureja gilliesii	shrub	
Spartothamnella puberula	shrub	
Teucrium chamaedrys	shrub	
Teucrium polium	shrub	
Thymus capitatus	shrub	
Thymus mongolicus	shrub	
Thymus nitens	shrub	
Thymus polytrichus	shrub	
Thymus pulegioides	shrub	
Thymus serpyllum	shrub	
Thymus vulgaris	shrub	

Vitex agnus-castus	shrub		
Vitex negundo	shrub		
Vitex negundo var. *cannabifolia*	shrub		
Vitex negundo var. *heterophylla*	shrub		
Vitex trifolia var. *simplicifolia*	shrub		
Aegiphila cf. *lhotzkiana*		tree	
Aegiphila costaricensis		tree	
Aegiphila sp.		tree	
Premna microphylla		tree	
Tectona grandis		tree	
Vitex micrantha		tree	
Vitex pinnata		tree	
**LARDIZABALACEAE**			
Akebia quinata			liana
Stauntonia hexaphylla			liana
**LAURACEAE**			
Lindera glauca	shrub	tree	
Cryptocarya alba	shrub		
Laurus nobilis	shrub		
Persea borbonia	shrub		
Persea vesticula	shrub		
Actinodaphne ambigua		tree	
Aiouea impressa		tree	
Alseodaphne hainanensis		tree	
Apollonias barbujana		tree	
Beilschmiedia erythrophloia		tree	
Beilschmiedia intermedia		tree	
Beilschmiedia tungfangensis		tree	
Cinnamomum barbeyanum		tree	
Cinnamomum burmannii		tree	
Cinnamomum calcareum		tree	
Cinnamomum camphora		tree	
Cinnamomum cf. *culilaban*		tree	
Cinnamomum japonicum		tree	
Cinnamomum porrectum		tree	
Cinnamomum verum		tree	
Cryptocarya chinensis		tree	
Cryptocarya chingii		tree	
Cryptocarya concinna		tree	
Cryptocarya densiflora		tree	
Cryptocarya mackinnoniana		tree	
Cryptocarya murrayi		tree	
Cryptocarya sp.		tree	
Cryptocarya triplinervis		tree	

Lauraceae sp.	tree
Laurus azorica	tree
Licaria sp.	tree
Licaria triandra	tree
Lindera reflexa	tree
Lindera umbellata	tree
Litsea coreana var. *sinensis*	tree
Litsea cubeba	tree
Litsea elongata	tree
Litsea exsudens	tree
Litsea glutinosa	tree
Machilus breviflora	tree
Machilus chinensis	tree
Machilus leptophylla	tree
Machilus monticola	tree
Machilus thunbergii	tree
Machilus viridis	tree
Nectandra ambigens	tree
Nectandra cufodontisii	tree
Nectandra purpurascens	tree
Nectandra purpurea	tree
Neolitsea aurata var. *chekiangensis*	tree
Neolitsea chui	tree
Neolitsea sericea	tree
Ocotea bullata	tree
Ocotea costulata	tree
Ocotea esmeraldana	tree
Ocotea fasciculata	tree
Ocotea foetens	tree
Ocotea guianensis	tree
Ocotea ira	tree
Ocotea leucoxylon	tree
Ocotea pittieri	tree
Ocotea rubra	tree
Ocotea sandwithii	tree
Ocotea spathulata	tree
Ocotea spp.	tree
Ocotea veraguensis	tree
Persea indica	tree
Persea lingue	tree
Persea nectandra	tree
Persea sp.	tree
Phoebe sheareri	tree
Sassafras tzumu	tree

Umbellularia californica		tree
**LECYTHIDACEAE**		
Barringtonia fusicarpa		tree
Cariniana decandra		tree
Cariniana estrellensis		tree
Cariniana ianeirensis		tree
Cariniana integrifolia		tree
Combretodendron macrocarpum		tree
Couratari guianensis		tree
Couratari sp.		tree
Eschweilera chartacea		tree
Eschweilera coriacea		tree
Eschweilera decolorans		tree
Eschweilera micrantha		tree
Eschweilera parviflora		tree
Eschweilera sagotiana		tree
Eschweilera sp.		tree
Gustavia hexapetala		tree
Lecythidaceae sp.		tree
Lecythis chartacea		tree
Lecythis corrugata		tree
Lecythis idatimon		tree
Lecythis lurida		tree
Lecythis persistens		tree
Lecythis zabucajo		tree
Planchonia careya		tree
Leguminosae sp		tree
**LINACEAE**		
Linum suffruticosum	shrub	
Hebepetalum humiriifolium		tree
Roucheria punctata		tree
**LOGANIACEAE**		
Fagraea fragrans		tree
Strychnos decussata		tree
Strychnos gerrardii		tree
Strychnos henningsii ssp. *gerrardii*		tree
Strychnos nux-vomica		tree
Nuytsia floribunda		tree
**LYTHRACEAE**		
Punica granatum	shrub	
Woodfordia fruticosa	shrub	
Lagerstroemia indica		tree
Lagerstroemia parviflora		tree
Lagerstroemia speciosa		tree

Lawsonia inermis		tree	
Sonneratia alba		tree	
**MAGNOLIACEAE**			
Elmerrillia tsiampacca		tree	
Liriodendron tulipifera		tree	
Magnolia fraseri		tree	
Magnolia grandiflora		tree	
Magnolia obovata		tree	
Magnolia salicifolia		tree	
Magnolia schiedeana		tree	
Magnolia splendens		tree	
Manglietia chingii		tree	
Manglietia glauca		tree	
Manglietia insignis		tree	
Michelia balansae		tree	
Michelia chapensis		tree	
Michelia macclurei		tree	
Michelia macclurei		tree	
Michelia nilagirica		tree	
Tsoongiodendron odorum		tree	
**MALPIGHIACEAE**			
Acridocarpus longifolius	shrub		
Acridocarpus smeathmannii	shrub		
Byrsonima crassifolia		tree	
Byrsonima lucida		tree	
Byrsonima wadsworthii		tree	
Heteropterys sp.		tree	
Banisteriopsis sp.			liana
Gaudichaudia mcvaughii			liana
Heteropterys palmeri			liana
Malpighiaceae sp.			liana
**MALVACEAE**			
Grewia biloba var. *parviflora*	shrub		
Helicteres angustifolia	shrub		
Hibiscus rosa-sinensis	shrub		
Lasiopetalum ferrugineum	shrub		
Lasiopetalum behrii	shrub		
Microcos paniculata	shrub		
Radyera farragei	shrub		
Sphaeralcea ambigua	shrub		
Adansonia digitata		tree	
Apeiba aspera		tree	
Apeiba burchellii		tree	
Apeiba echinata		tree	

Apeiba membranacea	tree
Apeiba schomburgkii	tree
Apeiba tibourbou	tree
Argyrodendron trifoliolatum	tree
Bombacopsis cf. *nervosa*	tree
Bombacopsis macrocalyx	tree
Bombacopsis nervosa	tree
Bombax ceiba	tree
Brachychiton megaphyllus	tree
Brachychiton populneus	tree
Burretiodendron hsienmu	tree
Catostemma fragrans	tree
Catostemma sp.	tree
Cavanillesia hylogeiton	tree
Ceiba pentandra	tree
Chorisia speciosa	tree
Cola greenwayi	tree
Commersonia bartramia	tree
Dombeya mukole	tree
Gossampinus malabarica	tree
Grewia asiatica	tree
Grewia cf. *glabra*	tree
Grewia flava	tree
Guazuma ulmifolia	tree
Hampea nutricia	tree
Heliocarpus americanus	tree
Heliocarpus appendiculatus	tree
Heliocarpus pallidus	tree
Heritiera trifoliolata	tree
Hibiscus tiliaceus	tree
Luehea candida	tree
Luehea seemannii	tree
Luehea speciosa	tree
Lueheopsis rugosa	tree
Mortoniodendron guatemalense	tree
Ochroma pyramidale	tree
Pachira quinata	tree
Pentapetes acerifolia	tree
Pseudobombax septenatum	tree
Pterospermum acerifolium	tree
Pterospermum heterophyllum	tree
Quararibea asterolepis	tree
Quararibea guatemalteca	tree
Quararibea muricata	tree

Quararibea yunckeri		tree
Rhodognaphalopsis discolor		tree
Rhodognaphalopsis humilis		tree
Robinsonella mirandae		tree
Sterculia alata		tree
Sterculia colorata		tree
Sterculia excelsa		tree
Sterculia lanceolata		tree
Sterculia sp.		tree
Tarrietia parvifolia		tree
Theobroma cacao		tree
Theobroma subincanum		tree
Thespesia populnea		tree
Tilia americana		tree
Tilia amurensis		tree
Tilia cordata		tree
Tilia japonica		tree
Tilia mandshurica		tree
Tilia maximowicziana		tree
Tilia mongolica		tree
Tilia platyphyllos		tree
Tilia sp.		tree
Trichospermum galeottii		tree
Trichospermum mexicanum		tree
**MARANTACEAE**		
Stromanthe jacquinii	shrub	
**MELASTOMATACEAE**		
Clidemia hirta	shrub	
Clidemia spp.	shrub	
Melastoma candidum	shrub	
Melastoma dodecandrum	shrub	
Melastoma malabathricum	shrub	
Memecylon eleagni	shrub	
Miconia fallax	shrub	
Bellucia grossularioides		tree
Blakea trinervia		tree
Calycogonium squamulosum		tree
Clidemia sericea		tree
Graffenrieda emarginata		tree
Loreya mespiloides		tree
Macairea rufescens		tree
Mecranium purpurascens		tree
Melastomataceae sp.		tree
Meriania purpurea		tree

Miconia albicans	tree	
Miconia argentea	tree	
Miconia borealis	tree	
Miconia calvescens	tree	
Miconia crassinervia	tree	
Miconia dispar	tree	
Miconia fragilis	tree	
Miconia ligustroides	tree	
Miconia media	tree	
Miconia prasina	tree	
Miconia rivetii	tree	
Miconia schnellii	tree	
Miconia tetrandra	tree	
Miconia tonduzii	tree	
Miconia tschudyoides	tree	
Mouriri crassifolia	tree	
Mouriri sp.	tree	
Mouriri uncitheca	tree	
Tibouchina stenocarpa	tree	
Topobea sp.		liana
**MELIACEAE**		
Aglaia elliptifolia	tree	
Aglaia sapindina	tree	
Amoora rohituka	tree	
Azadirachta indica	tree	
Carapa guianensis	tree	
Carapa procera	tree	
Cedrela fissilis	tree	
Cedrela odorata	tree	
Dysoxylum kusukusense	tree	
Dysoxylum sp.	tree	
Ekebergia capensis	tree	
Guarea glabra	tree	
Guarea grandifolia	tree	
Guarea guidonia	tree	
Guarea multiflora	tree	
Guarea pubescens	tree	
Guarea swartzii	tree	
Khaya grandiflora	tree	
Khaya ivorensis	tree	
Khaya nyasica	tree	
Khaya senegalensis	tree	
Lansium domesticum	tree	
Ptaeroxylon obliquum	tree	

Soymida febrifuga		tree	
Swietenia humilis		tree	
Swietenia macrophylla		tree	
Swietenia mahagoni		tree	
Swietenia sp.		tree	
Synoum glandulosum		tree	
Toona ciliata		tree	
Trichilia americana		tree	
Trichilia cipo		tree	
Trichilia glabra		tree	
Trichilia martiana		tree	
Trichilia sp.		tree	
Trichilia trifolia		tree	
**MELIANTHACEAE**			
Melianthus major	shrub		
**MENISPERMACEAE**			
Abuta cf. *grandifolia*			liana
Abuta panamensis			liana
Abuta rufescens			liana
Anomospermum reticulatum			liana
Cissampelos pareira			liana
**MONIMIACEAE**			
Peumus boldus	shrub		
Laurelia philippiana		tree	
Mollinedia darienensis		tree	
Xymalos monospora		tree	
**MORACEAE**			
Streblus ilicifolius	shrub		
Streblus taxoides	shrub		
Antiaris africana		tree	
Artocarpus heterophyllus		tree	
Artocarpus hypargyraea		tree	
Artocarpus lakoocha		tree	
Artocarpus tonkinensis		tree	
Batocarpus amazonicus		tree	
Bosqueia phoberos		tree	
Brosimum acutifolium		tree	
Brosimum alicastrum		tree	
Brosimum guianense		tree	
Brosimum lactescens		tree	
Brosimum mollis		tree	
Brosimum rubescens		tree	
Brosimum utile		tree	
Castilla elastica		tree	

Castilla ulei	tree
Chlorophora regia	tree
Clarisia biflora	tree
Clarisia racemosa	tree
Clarisia sp.	tree
Ficus benghalensis	tree
Ficus benjamina	tree
Ficus boliviana	tree
Ficus carica	tree
Ficus colubrinae	tree
Ficus erecta	tree
Ficus erecta var. *beecheyana*	tree
Ficus fistulosa	tree
Ficus gibbosa	tree
Ficus grossularioides	tree
Ficus insipida	tree
Ficus mucuso	tree
Ficus nodosa	tree
Ficus obtusifolia	tree
Ficus ovalis	tree
Ficus racemosa	tree
Ficus religiosa	tree
Ficus rumphii	tree
Ficus scobina	tree
Ficus tecolutensis	tree
Ficus yoponensis	tree
Helicostylis pedunculata	tree
Helicostylis tomentosa	tree
Maclura tinctoria	tree
Maquira calophylla	tree
Maquira guianensis	tree
Maquira sclerophylla	tree
Morus alba	tree
Naucleopsis glabra	tree
Naucleopsis imitans	tree
Naucleopsis sp.	tree
Naucleopsis ternstroemiiflora	tree
Poulsenia armata	tree
Pseudolmedia laevis	tree
Pseudolmedia oxyphyllaria	tree
Pseudolmedia sp.	tree
Pseudostreblus indicus	tree
Sorocea guilleminiana	tree
Trophis mexicana	tree

Trophis sp.		tree	
Trymatococcus amazonicus		tree	
Ficus deltoidea			liana
Ficus insculpta			liana
Ficus nipponica			liana
Ficus thunbergii			liana
**MYOPORACEAE**			
Myoporum bontioides	shrub		
**MYRICACEAE**			
Myrica aspleniifolia	shrub		
Myrica gale	shrub		
Comptonia peregrina		tree	
Myrica esculenta		tree	
Myrica faya		tree	
Myrica rubra		tree	
**MYRISTICACEAE**			
Compsoneura ulei		tree	
Iryanthera hostmannii		tree	
Iryanthera juruensis		tree	
Iryanthera sagotiana		tree	
Iryanthera sp.		tree	
Iryanthera ulei		tree	
Pycnanthus angolensis		tree	
Staudtia stipitata		tree	
Virola michelii		tree	
Virola nervosa		tree	
Virola pavonis		tree	
Virola sebifera		tree	
Virola surinamensis		tree	
**MYRTACEAE**			
Leptospermum myrsinoides	shrub	tree	
Baeckea crassifolia	shrub		
Baeckea frutescens	shrub		
Baeckea preissiana	shrub		
Calothamnus quadrifidus	shrub		
Calothamnus sanguineus	shrub		
Calytrix alpestris	shrub		
Calytrix flavescens	shrub		
Calytrix flavescens	shrub		
Calytrix tetragona	shrub		
Decaspermum gracilentum	shrub		
Eremaea pauciflora	shrub		
Eucalyptus drummondii	shrub		
Leptospermum erubescens	shrub		

Leptospermum laevigatum	shrub	
Leptospermum polygalifolium	shrub	
Leptospermum trinervium	shrub	
Melaleuca acerosa	shrub	
Melaleuca sp.	shrub	
Melaleuca trichophylla	shrub	
Melaleuca uncinata	shrub	
Micromyrtus sessilis	shrub	
Myrtus communis	shrub	
Regelia ciliata	shrub	
Rhodomyrtus tomentosa	shrub	
Scholtzia involucrata	shrub	
Verticordia nitens	shrub	
Acca sellowiana		tree
Acmena smithii		tree
Amomyrtus luma		tree
Angophora hispida		tree
Campomanesia aromatica		tree
Corymbia foelscheana		tree
Corymbia gummifera		tree
Eucalyptus ABL 12		tree
Eucalyptus acmenoides		tree
Eucalyptus amplifolia		tree
Eucalyptus baxteri		tree
Eucalyptus behriana		tree
Eucalyptus blakelyi		tree
Eucalyptus bleeseri		tree
Eucalyptus brevifolia		tree
Eucalyptus calophylla		tree
Eucalyptus camaldulensis		tree
Eucalyptus camaldulensis		tree
Eucalyptus clavigera		tree
Eucalyptus confertiflora		tree
Eucalyptus delegatensis		tree
Eucalyptus diversicolor		tree
Eucalyptus diversifolia		tree
Eucalyptus dives		tree
Eucalyptus dumosa		tree
Eucalyptus eugenioides		tree
Eucalyptus fastigata		tree
Eucalyptus foecunda		tree
Eucalyptus foliosa		tree
Eucalyptus fraxinoides		tree
Eucalyptus globulus		tree

Eucalyptus goniocalyx	tree
Eucalyptus grandis	tree
Eucalyptus haemastoma	tree
Eucalyptus hybrid	tree
Eucalyptus incrassata	tree
Eucalyptus intertexta	tree
Eucalyptus laevopinea	tree
Eucalyptus largiflorens	tree
Eucalyptus leucoxylon	tree
Eucalyptus macrorhyncha	tree
Eucalyptus maculata	tree
Eucalyptus mannifera	tree
Eucalyptus microcarpa	tree
Eucalyptus miniata	tree
Eucalyptus muelleriana	tree
Eucalyptus nesophila	tree
Eucalyptus nitens	tree
Eucalyptus obliqua	tree
Eucalyptus oblonga	tree
Eucalyptus occidentalis	tree
Eucalyptus paniculata	tree
Eucalyptus pauciflora	tree
Eucalyptus pilularis	tree
Eucalyptus porrecta	tree
Eucalyptus propinqua	tree
Eucalyptus pruinosa	tree
Eucalyptus regnans	tree
Eucalyptus rossii	tree
Eucalyptus saligna	tree
Eucalyptus sideroxylon	tree
Eucalyptus sideroxylon ssp. *tricarpa*	tree
Eucalyptus sieberi	tree
Eucalyptus socialis	tree
Eucalyptus sp.	tree
Eucalyptus tectifica	tree
Eucalyptus tereticornis	tree
Eucalyptus terminalis	tree
Eucalyptus tetrodonta	tree
Eucalyptus todtiana	tree
Eucalyptus umbra	tree
Eucalyptus urophylla	tree
Eucalyptus viminalis	tree
Eugenia borinquensis	tree
Eugenia casearioides	tree

Eugenia caudata	tree
Eugenia eggersii	tree
Eugenia florida	tree
Eugenia mabaeoides	tree
Eugenia maleolens	tree
Eugenia rhombea	tree
Eugenia rugosa	tree
Eugenia salamensis	tree
Eugenia sp.	tree
Eugenia spicata	tree
Eugenia stahlii	tree
Eugenia stapfiana	tree
Eugenia virgultosa	tree
Eugenia woodii	tree
Lophostemon confertus	tree
Lophostemon lactifluus	tree
Luma apiculata	tree
Melaleuca huegelii	tree
Melaleuca lanceolata	tree
Melaleuca leucadendra	tree
Melaleuca linariifolia	tree
Melaleuca nodosa	tree
Melaleuca sieberi	tree
Melaleuca viridiflora	tree
Metrosideros polymorpha	tree
Metrosideros umbellata	tree
Myrceugenia planipes	tree
Myrcia cuprea	tree
Myrcia deflexa	tree
Myrcia fallax	tree
Myrcia splendens	tree
Myrcianthes fragrans	tree
Myrcianthes sp.	tree
Pimenta racemosa	tree
Psidium cattleianum	tree
Psidium guajava	tree
Psidium sartorianum	tree
Rhodamnia cinerea	tree
Syncarpia glomulifera	tree
Syzygium araiocladum	tree
Syzygium confertum	tree
Syzygium cumini	tree
Syzygium eucalyptoides ssp. *bleeseri*	tree
Syzygium euphlebium	tree

Syzygium hainanense		tree	
Syzygium houttuynii		tree	
Syzygium jambos		tree	
Syzygium spp.		tree	
Syzygium suborbiculare		tree	
Tristaniopsis clementis		tree	
Tristaniopsis obovata		tree	
Tristaniopsis whiteana		tree	
Xanthostemon paradoxus		tree	
**NITRARIACEAE**			
Nitraria schoberi	shrub		
Nitraria sibirica	shrub		
Nitraria sphaerocarpa	shrub		
Nitraria tangutorum	shrub		
**NOTHOFAGACEAE**			
Fagus crenata		tree	
Nothofagus antarctica		tree	
Nothofagus betuloides		tree	
Nothofagus dombeyi		tree	
Nothofagus menziesii		tree	
Nothofagus moorei		tree	
Nothofagus nitida		tree	
Nothofagus obliqua		tree	
Nothofagus pumilio		tree	
Nothofagus truncata		tree	
**NYCTAGINACEAE**			
Bougainvillea spectabilis	shrub		
Guapira obtusata		tree	
Guapira opposita		tree	
Guapira sp.		tree	
Neea cf. *altissima*		tree	
Neea hermaphrodita		tree	
Neea obovata		tree	
Neea sp.		tree	
Pisonia albida		tree	
Pisonia subcordata		tree	
Pisonia aculeata			liana
**OCHNACEAE**			
Ouratea hexasperma	shrub		
Lophira alata		tree	
Ochna ciliata		tree	
Ochna natalitia		tree	
Ouratea littoralis		tree	
Touroulia guianensis		tree	

**OLACACEAE**		
Chaunochiton cf. *kappleri*		tree
Chaunochiton kappleri		tree
Coula edulis		tree
Heisteria spruceana		tree
Minquartia guianensis		tree
Strombosia scheffleri		tree
**OLEACEAE**		
Forsythia suspensa	shrub	
Jasminum odoratissimum	shrub	
Ligustrum japonicum	shrub	
Ligustrum retusum	shrub	
Ligustrum sinense	shrub	
Ligustrum sp.	shrub	
Ligustrum vulgare	shrub	
Menodora spinescens	shrub	
Phillyrea angustifolia	shrub	
Phillyrea latifolia	shrub	
Phillyrea media	shrub	
Syringa oblata	shrub	
Syringa reticulata	shrub	
Syringa vulgaris	shrub	
Chionanthus peglerae		tree
Chionanthus sp.		tree
Chionanthus virginicus		tree
Fraxinus americana		tree
Fraxinus angustifolia		tree
Fraxinus bungeana		tree
Fraxinus chinensis		tree
Fraxinus excelsior		tree
Fraxinus mandshurica		tree
Fraxinus nigra		tree
Fraxinus ornus		tree
Fraxinus oxycarpa		tree
Fraxinus pennsylvanica		tree
Fraxinus pennsylvanica var. *lanceolata*		tree
Fraxinus sp.		tree
Fraxinus sp.		tree
Fraxinus velutina cv. 'Modesto'		tree
Haenianthus incrassatus		tree
Ligustrum lucidum		tree
Nyctanthes arbor-tristis		tree
Olea capensis		tree
Olea capensis ssp. *macrocarpa*		tree

Olea europaea		tree	
Olea europaea ssp. *africana*		tree	
Olea woodiana		tree	
Osmanthus cooperi		tree	
Osmanthus marginatus		tree	
Picconia excelsa		tree	
**ONAGRACEAE**			
Fuchsia arborescens	shrub		
**VOCHYSIACEAE**			
Erisma densiflora		tree	
**PAEONIACEAE**			
Paeonia delavayi	shrub		
**PANDANACEAE**			
Freycinetia angustissima			liana
Freycinetia arborea			liana
Freycinetia sp.			liana
**PAPAVERACEAE**			
Bocconia frutescens	shrub		
**PARACRYPHIACEAE**			
Quintinia acutifolia		tree	
Sphenostemon papuantum		tree	
**PASSIFLORACEAE**			
Barteria fistulosa		tree	
Deidamia clematoides		tree	
Passiflora filipes			liana
Passiflora foetida			liana
Passiflora juliana			liana
**PAULOWNIACEAE**			
Paulownia tomentosa		tree	
**PENAEACEAE**			
Olinia ventosa		tree	
**PENTAPHYLLACEAE**			
Adinandra millettii	shrub		
Eurya brevistyla	shrub		
Eurya chinensis	shrub		
Adinandra dumosa		tree	
Adinandra hainanensis		tree	
Cleyera japonica		tree	
Cleyera theoides		tree	
Eurya japonica		tree	
Eurya rubiginosa		tree	
Eurya rubiginosa var. *attenuata*		tree	
Eurya sp.		tree	
Pentaphylax euryoides		tree	

Ternstroemia apleura		tree	
Ternstroemia gymnanthera		tree	
Ternstroemia stahlii		tree	
Visnea mocanera		tree	
**PHRYMACEAE**			
Diplacus aurantiacus	shrub		
Mimulus aurantiacus	shrub		
Phryma leptostachya			liana
**PHYLLANTHACEAE**			
Glochidion puberum	shrub		
Phyllanthus flexuosus	shrub		
Phyllanthus glaucus	shrub		
Phyllanthus hirtellus	shrub		
Phyllanthus reticulatus	shrub		
Amanoa congesta		tree	
Antidesma sp.		tree	
Aporosa aurea		tree	
Aporosa bracteosa		tree	
Aporosa chinensis		tree	
Aporosa falcifera		tree	
Aporosa globifera		tree	
Aporosa lunata		tree	
Aporosa microstachya		tree	
Aporosa nigricans		tree	
Aporosa prainiana		tree	
Aporosa spp.		tree	
Aporosa symplocoides		tree	
Aporosa yunnanensis		tree	
Baccaurea parviflora		tree	
Baccaurea racemosa		tree	
Baccaurea ramiflora		tree	
Baccaurea reticulata		tree	
Baccaurea sumatrana		tree	
Bischofia javanica		tree	
Bridelia insulana		tree	
Bridelia monoica		tree	
Bridelia retusa		tree	
Cleistanthus baramicus		tree	
Cleistanthus schlechteri		tree	
Hyeronima alchorneoides		tree	
Hymenocardia ulmoides		tree	
Margaritaria nobilis		tree	
Phyllanthus emblica		tree	
Protomegabaria stapfiana		tree	

Uapaca guineensis		tree
PHYLLANTHACEAE		
Uapaca staudtii		tree
PHYTOLACCACEAE		
Gallesia integrifolia		tree
PICRAMNIACEAE		
Picramnia sellowii		tree
PICRODENDRACEAE		
Petalostigma quadriloculare	shrub	
Celaenodendron mexicanum		tree
**PIPERACEAE**		
Piper amalago	shrub	tree
Piper auritum	shrub	tree
Piper aequale	shrub	
Piper cernuum	shrub	
Piper hispidum	shrub	
Piper lapathifolium	shrub	
Piper peltatum	shrub	
Piper umbellatum	shrub	
Piper plagiophyllum		tree
Piper sp.		tree
**PITTOSPORACEAE**		
Pittosporum glabratum	shrub	
**PLANTAGINACEAE**		
Globularia alypum	shrub	
Globularia cambessedesii	shrub	
Hebe argentea	shrub	
Hebe cockayneana	shrub	
Hebe franciscana	shrub	
Hebe franciscana	shrub	
Hebe hectorii	shrub	
Hebe odora	shrub	
Hebe parviflora	shrub	
Hebe pauciramosa	shrub	
Hebe subalpina	shrub	
**PLATANACEAE**		
Platanus acerifolia		tree
Platanus occidentalis		tree
Platanus orientalis		tree
**PLUMBAGINACEAE**		
Acantholimon diapensioides	shrub	
Limonium migjornense	shrub	
**POLYGALACEAE**		
Comesperma calymega	shrub	

Monnina crepinii	shrub		
Xanthophyllum hainanense		tree	
Xanthophyllum stipitatum		tree	
**POLYGONACEAE**			
Atraphaxis frutescens	shrub		
Atraphaxis spinosa	shrub		
Calligonum comosum	shrub		
Calligonum leucocladum	shrub		
Calligonum mongolicum	shrub		
Calligonum rigidum	shrub		
Calligonum roborowskii	shrub		
Eriogonum fasciculatum	shrub		
Eriogonum latifolium	shrub		
Polygonum cuspidatum	shrub		
Rumex lunaria	shrub		
Coccoloba barbadensis		tree	
Coccoloba diversifolia		tree	
Coccoloba hondurensis		tree	
Coccoloba microstachya		tree	
Triplaris americana		tree	
Afrobrunnichia erecta			liana
Antigonon flavescens			liana
**PRIMULACEAE**			
Aegiceras corniculatum	shrub	tree	
Ardisia crenata	shrub		
Ardisia punctata	shrub		
Ardisia quinquegona	shrub		
Ardisia revoluta	shrub		
Maesa japonica	shrub		
Myrsine africana	shrub		
Myrsine nummularia	shrub		
Myrsine sandwicensis	shrub		
Rapanea neriifolia	shrub		
Rapanea umbellata	shrub		
Ardisia costaricensis		tree	
Ardisia glauciflora		tree	
Ardisia sp.		tree	
Cybianthus marginatus		tree	
Heberdenia bahamensis		tree	
Jacquinia berteroi		tree	
Jacquinia pungens		tree	
Myrsine coriacea		tree	
Myrsine lessertiana		tree	
Myrsine seguinii		tree	

Myrsine sp.		tree
Parathesis amplifolia		tree
Rapanea melanophloeos		tree
Stylogyne ambigua		tree
**PROTEACEAE**		
Banksia marginata	shrub	tree
Banksia ornata	shrub	tree
Adenanthos cygnorum	shrub	
Aulax cneorifolia	shrub	
Aulax umbellata	shrub	
Banksia sphaerocarpa	shrub	
Conospermum stoechadis	shrub	
Conospermum triplinervium	shrub	
Diastella divaricata	shrub	
Dryandra cynaroides	shrub	
Dryandra nivea	shrub	
Dryandra sessilis	shrub	
Grevillea aneura	shrub	
Grevillea buxifolia	shrub	
Grevillea dimidiata	shrub	
Grevillea pyramidalis	shrub	
Grevillea speciosa	shrub	
Hakea acuminata	shrub	
Hakea adnata	shrub	
Hakea ambigua	shrub	
Hakea amplexicaulis	shrub	
Hakea anadenia	shrub	
Hakea auriculata	shrub	
Hakea baxteri	shrub	
Hakea bicornata	shrub	
Hakea brownii	shrub	
Hakea bucculenta	shrub	
Hakea candolleana	shrub	
Hakea ceratophylla	shrub	
Hakea cinerea	shrub	
Hakea commutata	shrub	
Hakea conchifolia	shrub	
Hakea corymbosa	shrub	
Hakea costata	shrub	
Hakea cristata	shrub	
Hakea cucullata	shrub	
Hakea cyclocarpa	shrub	
Hakea cygna ssp. *cygna*	shrub	
Hakea cygna ssp. *needlei*	shrub	

Hakea dactyloides	shrub
Hakea denticulata	shrub
Hakea drupacea	shrub
Hakea elliptica	shrub
Hakea eneabba	shrub
Hakea erecta	shrub
Hakea erinacea	shrub
Hakea falcata	shrub
Hakea ferruginea	shrub
Hakea flabellifolia	shrub
Hakea florida	shrub
Hakea francisiana	shrub
Hakea gilbertii	shrub
Hakea hookeriana	shrub
Hakea horrida	shrub
Hakea ilicifolia	shrub
Hakea incrassata	shrub
Hakea invaginata	shrub
Hakea kippistiana	shrub
Hakea lasiantha	shrub
Hakea lasiocarpha	shrub
Hakea laurina	shrub
Hakea lehmanniana	shrub
Hakea linearis	shrub
Hakea lissocarpha	shrub
Hakea longiflora	shrub
Hakea marginata	shrub
Hakea megalosperma	shrub
Hakea minyma	shrub
Hakea muellerana	shrub
Hakea multilineata	shrub
Hakea myrtoides	shrub
Hakea neurophylla	shrub
Hakea newbeyana	shrub
Hakea nitida	shrub
Hakea obliqua ssp. *parviflora*	shrub
Hakea obtusa	shrub
Hakea oleifolia	shrub
Hakea orthorryncha var. *filiformis*	shrub
Hakea orthorryncha var. *orthorryncha*	shrub
Hakea pandicarpa crassifolia	shrub
Hakea pandicarpa pandicarpa	shrub
Hakea petiolaris	shrub

Hakea platysperma D2983	shrub
Hakea polyanthema	shrub
Hakea preissii	shrub
Hakea pritzelii	shrub
Hakea psilorryncha	shrub
Hakea pycnoneura	shrub
Hakea recurva ssp. *arida*	shrub
Hakea recurva ssp. *recurva*	shrub
Hakea rostrata	shrub
Hakea ruscifolia	shrub
Hakea ryscifolia	shrub
Hakea scoparia ssp. *scoparia*	shrub
Hakea scoparia ssp. *trycherica*	shrub
Hakea spathulata	shrub
Hakea stenocarpa	shrub
Hakea stenophylla	shrub
Hakea strumosa	shrub
Hakea subsulcata	shrub
Hakea sulcata	shrub
Hakea tephrosperma	shrub
Hakea teretifolia	shrub
Hakea trifurcata	shrub
Hakea tuberculata	shrub
Hakea undulata	shrub
Hakea varia	shrub
Hakea verrucosa	shrub
Hakea victoria	shrub
Lambertia formosa	shrub
Leucadendron arcuatum	shrub
Leucadendron brunioides	shrub
Leucadendron conifesula	shrub
Leucadendron foedum	shrub
Leucadendron lanigerum	shrub
Leucadendron laureolum	shrub
Leucadendron pubescens	shrub
Leucadendron salignum	shrub
Leucadendron xanthoconus	shrub
Leucospermum conocarpodendron	shrub
Leucospermum cordifolium	shrub
Leucospermum hypophyllocarpodendron	shrub
Leucospermum truncatulum	shrub
Lomatia silaifolia	shrub
Mimetes cucullatus	shrub

Mimetes fibrifolium	shrub	
Paranomus bracteolaris	shrub	
Persoonia falcata	shrub	
Persoonia laevis	shrub	
Persoonia saccata	shrub	
Petrophile linearis	shrub	
Petrophile medina	shrub	
Protea acaulos	shrub	
Protea bracteolaris	shrub	
Protea burchellii	shrub	
Protea compacta	shrub	
Protea cordata	shrub	
Protea glabra	shrub	
Protea laurifolia	shrub	
Protea neriifolia	shrub	
Protea nitida	shrub	
Protea repens	shrub	
Protea speciosa	shrub	
Protea susannae	shrub	
Roupala montana	shrub	
Serruria fasciflora	shrub	
Serruria fucifolia	shrub	
Serruria hirsuta	shrub	
Serruria nervosa	shrub	
Serruria sp.	shrub	
Stirlingia latifolia	shrub	
Athertonia diversifolia		tree
Banksia aemula		tree
Banksia attenuata		tree
Banksia coccinea		tree
Banksia grandis		tree
Banksia hookeriana		tree
Banksia marginata		tree
Banksia menziesii		tree
Banksia oblongifolia		tree
Banksia robur		tree
Banksia serrata		tree
Brabejum stellatifolium		tree
Cardwellia sublimis		tree
Darlingia darlingiana		tree
Embothrium coccineum		tree
Faurea macnaughtonii		tree
Gevuina avellana		tree
Grevillea anethifolia		tree

Grevillea annulifera		tree	
Grevillea hugelii		tree	
Grevillea juniperina		tree	
Grevillea leucopteris		tree	
Grevillea robusta		tree	
Grevillea sericea		tree	
Grevillea thelemanniana		tree	
Hakea gibbosa		tree	
Hakea arborescens		tree	
Hakea circumalata		tree	
Hakea leucoptera		tree	
Hakea obliqua		tree	
Helicia cochinchinensis		tree	
Helicia formosana		tree	
Helicia rengetiensis		tree	
Lomatia hirsuta		tree	
Persoonia lanceolata		tree	
Xylomelum angustifolium		tree	
Xylomelum pyriforme		tree	
**PUTRANJIVACEAE**			
Drypetes arguta		tree	
Drypetes hieranensis		tree	
Drypetes natalensis		tree	
Drypetes variabilis		tree	
**QUILLAJACEAE**			
Quillaja saponaria	shrub		
**RANUNCULACEAE**			
Clematis obscura	shrub		
Helleborus foetidus	shrub		
Helleborus lividus	shrub		
Ranunculus auricomus	shrub		
Clematis flammula			liana
Clematis florida			liana
Clematis hexapetala			liana
Clematis lasiantha			liana
Clematis orientalis			liana
Clematis recta			liana
Clematis tangutica			liana
Clematis terniflora			liana
Clematis vitalba			liana
**RESTIONACEAE**			
Empodisma minus	shrub		
Hypolaena fastigiata		tree	
**RHAMNACEAE**			

Ceanothus americanus	shrub	
Ceanothus crassifolius	shrub	
Ceanothus cuneatus	shrub	
Ceanothus dentatus	shrub	
Ceanothus greggii	shrub	
Ceanothus herbaceus	shrub	
Ceanothus megacarpus	shrub	
Ceanothus oliganthus	shrub	
Ceanothus oliganthus ssp. *sorediatus*	shrub	
Ceanothus spinosus	shrub	
Ceanothus velutinus	shrub	
Condalia hookeri	shrub	
Frangula alnus	shrub	
Pomaderris ferruginea	shrub	
Rhamnus alaternus	shrub	
Rhamnus arguta	shrub	
Rhamnus californica	shrub	
Rhamnus cathartica	shrub	
Rhamnus davurica	shrub	
Rhamnus frangula	shrub	
Rhamnus ludovici-salvatoris	shrub	
Rhamnus lycioides	shrub	
Rhamnus oreodendron	shrub	
Rhamnus parvifolia	shrub	
Rhamnus sp.	shrub	
Rhamnus ussuriensis	shrub	
Spyridium oligocephalum	shrub	
Spyridium subochreatum	shrub	
Trevoa trinervis	shrub	
Trymalium ledifolium	shrub	
Ziziphus jujuba var. *spinosa*	shrub	
Ziziphus obtusifolia	shrub	
Colubrina arborescens		tree
Colubrina texensis		tree
Karwinskia calderonii		tree
Krugiodendron ferreum		tree
Paliurus hemsleyanus		tree
Rhamnus crocea		tree
Rhamnus glandulosa		tree
Ziziphus glaberrima		tree
Ziziphus jujuba		tree
Ziziphus mistol		tree
Ziziphus mucronata		tree

Zizyphus glaberrima		tree	
Berchemia racemosa			liana
Berchemia sp.			liana
Gouania rosei			liana
Ventilago viminalis			liana
**RHIZOPHORACEAE**			
Bruguiera gymnorrhiza	shrub	tree	
Kandelia candel	shrub	tree	
Rhizophora mucronata	shrub	tree	
Bruguiera conjugata	shrub		
Bruguiera sexangula	shrub		
Ceriops tagal	shrub		
Rhizophora apiculata	shrub		
Bruguiera cylindrica		tree	
Cassipourea ruwensorensis		tree	
Ceriops australis		tree	
Rhizophora mangle		tree	
Rhizophora stylosa		tree	
**ROSACEAE**			
Prunus lusitanica	shrub	tree	
Prunus mahaleb	shrub	tree	
Prunus serotina	shrub	tree	
Prunus virginiana	shrub	tree	
Adenostoma fasciculatum	shrub		
Amelanchier humilis	shrub		
Amelanchier lamarckii	shrub		
Amelanchier ovalis	shrub		
Amelanchier sp.	shrub		
Amelanchier spicata	shrub		
Amygdalus arabica	shrub		
Aronia melanocarpa	shrub		
Cercocarpus betuloides	shrub		
Coleogyne ramosissima	shrub		
Comarum salesovianum	shrub		
Cotoneaster acutifolius	shrub		
Cotoneaster acutifolius	shrub		
Cotoneaster adpressus	shrub		
Cotoneaster integerrimus	shrub		
Crataegus cuneata	shrub		
Crataegus laevigata	shrub		
Crataegus macrocarpa	shrub		
Crataegus monogyna	shrub		
Crataegus pinnatifida	shrub		
Crataegus sp.	shrub		

Dasiphora floribunda	shrub
Dryas integrifolia	shrub
Dryas octopetala	shrub
Fallugia paradoxa	shrub
Heteromeles arbutifolia	shrub
Holodiscus discolor	shrub
Kageneckia angustifolia	shrub
Kageneckia oblonga	shrub
Pentaphylloides dryadanthoides	shrub
Pentaphylloides fruticosa	shrub
Photinia parvifolia	shrub
Physocarpus opulifolius	shrub
Potentilla davurica	shrub
Potentilla fruticosa	shrub
Potentilla glabra	shrub
Potentilla parvifolia	shrub
Prinsepia uniflora	shrub
Prunus davidiana	shrub
Prunus ilicifolia	shrub
Prunus laurocerasus	shrub
Prunus pilosiuscula	shrub
Prunus pumila	shrub
Prunus setulosa	shrub
Prunus spinosa	shrub
Purshia tridentata	shrub
Pyracantha fortuneana	shrub
Pyrus betulifolia	shrub
Pyrus pyrifolia	shrub
Rhaphiolepis indica	shrub
Rosa agrestis	shrub
Rosa arkansana	shrub
Rosa blanda	shrub
Rosa caesia	shrub
Rosa canina	shrub
Rosa ciesielskii	shrub
Rosa coriifolia	shrub
Rosa davurica	shrub
Rosa dumalis	shrub
Rosa glauca	shrub
Rosa hugonis	shrub
Rosa majalis	shrub
Rosa micrantha	shrub
Rosa mollis	shrub
Rosa rugosa	shrub

Rosa spinosissima	shrub	
Rosa subcanina	shrub	
Rosa xanthina	shrub	
Rubus allegheniensis	shrub	
Rubus argutus	shrub	
Rubus caesius	shrub	
Rubus canescens	shrub	
Rubus chamaemorus	shrub	
Rubus crataegifolius	shrub	
Rubus discolor	shrub	
Rubus hawaiiensis	shrub	
Rubus hispidus	shrub	
Rubus idaeus	shrub	
Rubus innominatus	shrub	
Rubus occidentalis	shrub	
Rubus parvifolius	shrub	
Rubus pubescens	shrub	
Rubus saxatilis	shrub	
Rubus sp.	shrub	
Rubus strigosus	shrub	
Rubus ursinus	shrub	
Rubus vestitus	shrub	
Sarcopoterium spinosum	shrub	
Sieversia pentapetala	shrub	
Sorbaria sorbifolia	shrub	
Sorbus hupehensis var. *aperta*	shrub	
Sorbus sp.	shrub	
Spiraea alba	shrub	
Spiraea alpina	shrub	
Spiraea aquilegiifolia	shrub	
Spiraea chamaedryfolia	shrub	
Spiraea dasyantha	shrub	
Spiraea kwangsiensis	shrub	
Spiraea mongolica	shrub	
Spiraea pubescens	shrub	
Spiraea salicifolia	shrub	
Spiraea sp.	shrub	
Spiraea tomentosa	shrub	
Amelanchier alnifolia		tree
Crataegus curvisepala		tree
Eriobotrya japonica		tree
Hagenia abyssinica		tree
Malus baccata		tree
Malus domestica		tree

Malus pumila		tree	
Malus sp.		tree	
Malus sylvestris		tree	
Padus avium		tree	
Padus sp.		tree	
Photinia davidiana		tree	
Photinia glabra		tree	
Photinia prunifolia var. *consimilis*		tree	
Polylepis australis		tree	
Polylepis rugulosa		tree	
Prunus annularis		tree	
Prunus armeniaca		tree	
Prunus avium		tree	
Prunus cerasus		tree	
Prunus humilis		tree	
Prunus padus		tree	
Prunus pensylvanica		tree	
Prunus sargentii		tree	
Prunus serrulata		tree	
Prunus sibirica		tree	
Prunus ssiori		tree	
Prunus turneriana		tree	
Prunus vulgaris		tree	
Pyrus amygdaliformis		tree	
Pyrus bourgaeana		tree	
Pyrus calleryana 'Bradford'		tree	
Pyrus communis		tree	
Pyrus pyraster		tree	
Pyrus sp.		tree	
Sorbus alnifolia		tree	
Sorbus aria		tree	
Sorbus aucuparia		tree	
Sorbus commixta		tree	
Sorbus folgneri		tree	
Sorbus intermedia		tree	
Rosa arvensis			liana
Rubus corylifolius			liana
Rubus fruticosus			liana
Rubus ulmifolius			liana
Rubus utchinensis			liana
**RUBIACEAE**			
Rubiaceae spp.	shrub	tree	
Amaioua corymbosa	shrub		
Bouvardia ternifolia	shrub		

Canthium bibracteatum	shrub	
Coprosma cheesemanii	shrub	
Coprosma ciliata	shrub	
Coprosma ernodeoides	shrub	
Coprosma parviflora	shrub	
Coprosma rugosa	shrub	
Coprosma serrulata	shrub	
Coprosma spp.	shrub	
Gardenia jasminoides	shrub	
Hamelia patens	shrub	
Hedyotis centranthoides	shrub	
Ixora alba	shrub	
Ixora coccinea	shrub	
Ixora congesta	shrub	
Leptodermis microphylla	shrub	
Luculia gratissima	shrub	
Palicourea sp.	shrub	
Psychotria furcata	shrub	
Psychotria hainanensis	shrub	
Psychotria limonensis	shrub	
Psychotria marginata	shrub	
Psychotria rubra	shrub	
Psychotria sp	shrub	
Rubia manjith	shrub	
Rubia peregrina	shrub	
Serissa foetida	shrub	
Tarenna depauperata	shrub	
Tarenna fragrans	shrub	
Adina cordifolia		tree
Aidia micrantha		tree
Aidia ochroleuca		tree
Alibertia verrucosa		tree
Alseis blackiana		tree
Anthocephalus cadamba		tree
Antirrhoea trichantha		tree
Calycophyllum candidissimum		tree
Canthium dicoccum		tree
Chomelia spinosa		tree
Coffea arabica		tree
Coffea canephora		tree
Coprosma ochracea		tree
Diplospora malaccensis		tree
Duroia eriopila		tree
Duroia macrophylla		tree

Erithalis fruticosa		tree	
Exostema caribaeum		tree	
Exostema mexicanum		tree	
Faramea occidentalis		tree	
Gardenia latifolia		tree	
Genipa americana		tree	
Genipa caruto		tree	
Guettarda macrosperma		tree	
Guettarda scabra		tree	
Guettarda spruceana		tree	
Hyperacanthus amoenus		tree	
Macrocnemum glabrescens		tree	
Mitragyna parvifolia		tree	
Neolaugeria resinosa		tree	
Paederia scandens		tree	
Palicourea crocea		tree	
Palicourea riparia		tree	
Paragenipa wrightii		tree	
Posoqueria latifolia		tree	
Psychotria berteroana		tree	
Psychotria capensis		tree	
Psychotria chiapensis		tree	
Psychotria faxlucens		tree	
Psychotria flava		tree	
Psychotria simiarum		tree	
Psychotria sp.		tree	
Psychotria suerrensis		tree	
Randia cochinchinensis		tree	
Remijia morilloi		tree	
Retiniphyllum concolor		tree	
Retiniphyllum truncatum		tree	
Rothmannia globosa		tree	
Rudgea crassiloba		tree	
Scyphiphora hydrophyllacea		tree	
Tocoyena formosa		tree	
Tricalysia dubia		tree	
Xeromphis uliginosa		tree	
Coptosapelta diffusa			liana
Morinda umbellata			liana
Mussaenda parviflora			liana
Psychotria serpens			liana
**RUTACEAE**			
Correa reflexa	shrub	tree	
Choisya ternata	shrub		

Cneorum tricoccon	shrub		
Eriostemon australasius	shrub		
Murraya koenigii	shrub		
Philotheca difformis	shrub		
Zanthoxylum alatum	shrub		
Zanthoxylum avicennae	shrub		
Zanthoxylum fagara	shrub		
Zanthoxylum planispinum	shrub		
Zanthoxylum simulans	shrub		
Zanthoxylum sprucei	shrub		
Acronychia pedunculata		tree	
Adiscanthus fusciflorus		tree	
Aegle marmelos		tree	
Boronia ledifolia		tree	
Calodendrum capense		tree	
Citrus paradisi		tree	
Clausena anisata		tree	
Erythrochiton fallax		tree	
Flindersia maculosa		tree	
Geijera parviflora		tree	
Melicope denhamii		tree	
Metrodorea flavida		tree	
Pelea sp.		tree	
Phellodendron amurense		tree	
Teclea gerrardii		tree	
Teclea natalensis		tree	
Teclea nobilis		tree	
Tetradium glabrifolium		tree	
Vepris undulata		tree	
Zanthoxylum capense		tree	
Zanthoxylum ekmanii		tree	
Zanthoxylum kellermanii		tree	
Zanthoxylum panamense		tree	
Zanthoxylum scandens			liana
**SABIACEAE**			
Meliosma oldhamii		tree	
Meliosma rigida		tree	
**SALICACEAE**			
Salix acutifolia	shrub	tree	
Salix pentandra	shrub	tree	
Salix spp.	shrub	tree	
Azara dentata	shrub		
Azara lanceolata	shrub		
Salix alaxensis	shrub		

Salix amygdaloides	shrub	
Salix anticecrenata	shrub	
Salix arctica	shrub	
Salix atrocinerea	shrub	
Salix aurita	shrub	
Salix aurita x cinerea	shrub	
Salix bebbiana	shrub	
Salix canariensis	shrub	
Salix candida	shrub	
Salix cheilophila	shrub	
Salix cinerea	shrub	
Salix dasyclados	shrub	
Salix dasyclados x phylicifolia	shrub	
Salix flavida	shrub	
Salix fuscescens	shrub	
Salix glauca	shrub	
Salix hastata	shrub	
Salix herbacea	shrub	
Salix lapponum	shrub	
Salix microstachya	shrub	
Salix mongolica	shrub	
Salix myrsinifolia	shrub	
Salix myrsinifolia × *lapponum*	shrub	
Salix myrsinites	shrub	
Salix myrtilloides	shrub	
Salix oritrepha	shrub	
Salix phylicifolia	shrub	
Salix planifolia	shrub	
Salix polaris	shrub	
Salix psammophila	shrub	
Salix pulchra	shrub	
Salix purpurea	shrub	
Salix repens	shrub	
Salix reticulata	shrub	
Salix rosmarinifolia	shrub	
Salix souliei	shrub	
Salix starkeana	shrub	
Salix triandra	shrub	
Salix viminalis	shrub	
Abatia parviflora		tree
Casearia arborea		tree
Casearia arguta		tree
Casearia corymbosa		tree
Casearia gossypiosperma		tree

Casearia nitida	tree
Casearia silvestris	tree
Casearia sylvestris	tree
Dovyalis longispina	tree
Homalium dictyoneurum	tree
Laetia procera	tree
Lunania mexicana	tree
Pleuranthodendron lindenii	tree
Populus adenopoda	tree
Populus alba	tree
Populus alba var. *pyramidalis*	tree
Populus balsamifera	tree
Populus cathayana	tree
Populus davidiana	tree
Populus deltoides	tree
Populus deltoides × *P. trichopcarpa*	tree
Populus euphratica	tree
Populus fremontii	tree
Populus grandidentata	tree
Populus heterophylla	tree
Populus hopeiensis	tree
Populus maximowiczii	tree
Populus nigra	tree
Populus nigra var. *italica*	tree
Populus pruinosa	tree
Populus sieboldii	tree
Populus simonii	tree
Populus sp.	tree
Populus tremula	tree
Populus tremuloides	tree
Populus trichocarpa	tree
Populus trichocarpa x P. deltoides	tree
Populus trichocarpa x P. maximowiczii	tree
Ryania speciosa	tree
Salix alba	tree
Salix alba x fragilis	tree
Salix caprea	tree
Salix daphnoides	tree
Salix fragilis	tree
Salix hultenii	tree
Salix laevigata	tree
Salix matsudana	tree
Salix nigra	tree

Scolopia crassipes		tree
Scolopia mundii		tree
Scolopia zeyheri		tree
Xylosma schwaneckeana		tree
Zuelania guidonia		tree
**SANTALACEAE**		
Exocarpos aphyllus	shrub	
Santalum lanceolatum	shrub	
Acanthosyris falcata		tree
Acanthosyris glabrata		tree
Exocarpos apophyllum		tree
Santalum acuminatum		tree
**SAPINDACEAE**		
Acer ginnala	shrub	tree
Acer erianthum	shrub	
Acer spicatum	shrub	
Acer tataricum	shrub	
Dodonaea attenuata	shrub	
Dodonaea triquetra	shrub	
Dodonaea viscosa	shrub	
Dodonaea viscosa ssp. *angustissima*	shrub	
Dodonaea viscosa ssp. *cuneata*	shrub	
Dodonaea viscosa ssp. *spatulata*	shrub	
Dodonaea viscosa var. *spatulata*	shrub	
Valenzuelia trinervis	shrub	
Xanthoceras sorbifolia	shrub	
Acer campestre		tree
Acer lucidum		tree
Acer mandshuricum		tree
Acer mono		tree
Acer mono ssp. *marmoratum f. dissectum*		tree
Acer monspessulanum		tree
Acer negundo		tree
Acer opalus		tree
Acer palmatum		tree
Acer pensylvanicum		tree
Acer pensylvanicum		tree
Acer platanoides		tree
Acer pseudoplatanus		tree
Acer pseudosieboldianum		tree
Acer rubrum		tree
Acer saccharinum		tree
Acer saccharinum		tree
Acer saccharum		tree

Acer sieboldianum	tree	
Acer sinense	tree	
Acer sp.	tree	
Acer tegmentosum	tree	
Acer truncatum	tree	
Aesculus californica	tree	
Aesculus hippocastanum	tree	
Aesculus turbinata	tree	
Allophylus camptostachys	tree	
Allophylus occidentalis	tree	
Allophylus varians	tree	
Amesiodendron chinense	tree	
Castanospora alphandi	tree	
Cupania cinerea	tree	
Cupania dentata	tree	
Cupania rufescens	tree	
Cupania sylvatica	tree	
Dilodendron costaricense	tree	
Dimocarpus longan	tree	
Hippobromus pauciflorus	tree	
Koelreuteria minor	tree	
Litchi chinensis	tree	
Nephelium lappaceum var. *topengii*	tree	
Pancovia golungensis	tree	
Pancovia turbinata	tree	
Sapindus mukorossi	tree	
Sapindus saponaria	tree	
Schleichera oleosa	tree	
Thouinia paucidentata	tree	
Thouinia striata	tree	
Thouinidium decandrum	tree	
Toulicia reticulata	tree	
Cardiospermum halicacabum		liana
Paullinia fibrigera		liana
Serjania brachycarpa		liana
**SAPOTACEAE**		
Afrosersalisia afzelii	tree	
Bequaertiodendron natalense	tree	
Chrysophyllum argenteum	tree	
Chrysophyllum cainito	tree	
Chrysophyllum prieurii	tree	
Chrysophyllum sanguinolentum	tree	
Ecclinusa guianensis	tree	
Ganua sp.	tree	

Inhambanella henriquesii	tree
Jacquinia revoluta	tree
Madhuca hainanensis	tree
Madhuca indica	tree
Manilkara bidentata	tree
Manilkara chicle	tree
Manilkara longiciliata	tree
Micropholis cf. *venulosa*	tree
Micropholis chrysophylloides	tree
Micropholis guyanensis	tree
Micropholis maguirei	tree
Micropholis maguirei	tree
Micropholis obscura	tree
Micropholis sp.	tree
Micropholis venulosa	tree
Mimusops elengi	tree
Mimusops hexandra	tree
Mimusops obovata	tree
Northia hornei	tree
Planchonella firma	tree
Planchonella obovata	tree
Pouteria cf. *pachycarpa*	tree
Pouteria durlandii	tree
Pouteria eugeniifolia	tree
Pouteria grandis	tree
Pouteria guianensis	tree
Pouteria hispida	tree
Pouteria macrophylla	tree
Pouteria melanopoda	tree
Pouteria nemorosa	tree
Pouteria oblanceolata	tree
Pouteria reticulata	tree
Pouteria rhynchocarpa	tree
Pouteria sp.	tree
Pouteria surumuensis	tree
Pouteria torta	tree
Pouteria venosa	tree
Pradosia cochlearia	tree
Pradosia surinamensis	tree
Sideroxylon capiri	tree
Sideroxylon inerme	tree
**SCHIZANDRACEAE**	
Illicium anisatum	tree
Illicium lanceolatum	tree

Illicium ternstroemioides		tree	
Illicium verum		tree	
Kadsura japonica			liana
Schisandra chinensis			liana
**SCROPHULARIACEAE**			
Buddleja davidii	shrub		
Eremophila deserti	shrub		
Eremophila foliosissima	shrub		
Eremophila fraseri	shrub		
Eremophila glabra	shrub		
Eremophila latrobei	shrub		
Eremophila longifolia	shrub		
Eremophila mitchelli	shrub		
Eremophila pterocarpa	shrub		
Buddleja brasiliensis		tree	
Buddleja nitida		tree	
Freylinia lanceolata		tree	
Myoporum acuminatum		tree	
Myoporum sandwicense		tree	
Wightia speciosissima		tree	
**SIMAROUBACEAE**			
Ailanthus altissima		tree	
Ailanthus excelsa		tree	
Eurycoma longifolia		tree	
Hannoa klaineana		tree	
Simaba cedron		tree	
Simarouba amara		tree	
Simarouba glauca		tree	
Soulamea terminalioides		tree	
**SIMMONDSIACEAE**			
Simmondsia chinensis	shrub		
**SIPARUNACEAE**			
Coprosma alpina	shrub		
**SMILACACEAE**			
Smilax vaginata	shrub		
Smilax aspera			liana
Smilax mollis			liana
Smilax sp.			liana
**SOLANACEAE**			
Solanum straminifolia	shrub	tree	
Dunalia spinosa	shrub		
Fabiana densa var. *ramulosa*	shrub		
Lycium andersonii	shrub		
Lycium berlandieri	shrub		

Lycium chinense	shrub		
Lycium chinense	shrub		
Lycium elongatum	shrub		
Lycium pallidum	shrub		
Lycium ruthenicum	shrub		
Lycium shockleyi	shrub		
Solanum ferocissimum	shrub		
Solanum umbelliferum	shrub		
Nicotiana glauca		tree	
Solanum punctulatum		tree	
Solanum dulcamara			liana
Solanum refractum			liana
**STAPHYLEACEAE**			
Turpinia insignis		tree	
Turpinia occidentalis		tree	
Turpinia ternata		tree	
**STYRACACEAE**			
Alniphyllum fortunei		tree	
Pterostyrax hispidus		tree	
Styrax argenteus		tree	
Styrax camporum		tree	
Styrax suberifolius		tree	
**SURIANACEAE**			
Guilfoylia monostylis		tree	
**SYMPLOCACEAE**			
Symplocos anomala		tree	
Symplocos austin-smithii		tree	
Symplocos heishanensis		tree	
Symplocos lancifolia		tree	
Symplocos laurina		tree	
Symplocos prunifolia		tree	
Symplocos serrulata		tree	
Symplocos stellaris		tree	
Symplocos sumuntia		tree	
**TAMARICACEAE**			
Myricaria elegans	shrub		
Myricaria squamosa	shrub		
Reaumuria soongarica	shrub		
Tamarix hispida	shrub		
Tamarix juniperina	shrub		
Tamarix laxa	shrub		
Tamarix ramosissima	shrub		
Tamarix sp.	shrub		
**THEACEAE**			

Camellia cuspidata	shrub	
Camellia drupifera	shrub	
Camellia elongata	shrub	
Camellia oleifera	shrub	
Camellia fraterna		tree
Camellia gigantocarpa		tree
Camellia japonica		tree
Camellia reticulata		tree
Camellia sinensis var. *assamica*		tree
Laplacea haematoxylon		tree
Polyspora axillaris		tree
Schima argentea		tree
Schima brevifolia		tree
Schima noronhae		tree
Schima superba		tree
Schima wallichii		tree
**THYMELACEAE**		
Daphne gnidium	shrub	
Daphne mezereum	shrub	
Dirca occidentalis	shrub	
Dirca palustris	shrub	
Pimelea linifolia	shrub	
Pimelea microcephala	shrub	
Thymelaea hirsuta	shrub	
Thymelaea tartonraira	shrub	
Wikstroemia chamaedaphne	shrub	
Wikstroemia dolichantha	shrub	
Daphnopsis philippiana		tree
Ovidia pillopillo		tree
**ULMACEAE**		
Ampelocera ruizii		tree
Chaetachme aristata		tree
Holoptelea integrifolia		tree
Ulmus americana		tree
Ulmus davidiana		tree
Ulmus glabra		tree
Ulmus glaucescens		tree
Ulmus laciniata		tree
Ulmus laevis		tree
Ulmus macrocarpa		tree
Ulmus parvifolia		tree
Ulmus procera		tree
Ulmus propinqua		tree
Ulmus pumila		tree

Ulmus rubra		tree	
Ulmus sp.		tree	
Ulmus suberosa		tree	
Ulmus thomasii		tree	
Ulmus tonkinensis		tree	
Zelkova schneideriana		tree	
Zelkova serrata		tree	
**URTICACEAE**			
Boehmeria sp.	shrub		
Myriocarpa longipes	shrub		
Urera elata	shrub		
Cecropia concolor		tree	
Cecropia ficifolia		tree	
Cecropia insignis		tree	
Cecropia longipes		tree	
Cecropia obtusifolia		tree	
Cecropia peltata		tree	
Cecropia schreberiana		tree	
Cecropia sciadophylla		tree	
Cecropia sp.		tree	
Laportea pterostigma		tree	
Musanga cecropioides		tree	
Myrianthus libericus		tree	
Pipturus albidus		tree	
Pipturus argenteus		tree	
Pourouma bicolor		tree	
Pourouma velutina		tree	
Urera caracasana		tree	
**VERBENACEAE**			
Rhaphithamnus spinosus	shrub		
Citharexylum macradenium		tree	
Citharexylum macrophyllum		tree	
Rehdera trinervis		tree	
**VIOLACEAE**			
Amphirrhox cf. *latifolia*		tree	
Leonia cf. *crassa*		tree	
Melicytus ramiflorus		tree	
Orthion oblanceolatum		tree	
Paypayrola guianensis		tree	
**VITACEAE**			
Rhoicissus tomentosa		tree	liana
Cissus producta		tree	
Ampelopsis aconitifolia			liana
Ampelopsis sp.			liana

Cissus sicyoides			liana
Cissus sp.			liana
Parthenocissus quinquefolia			liana
Parthenocissus quinquefolia var. *murorum*			liana
Parthenocissus vitacea			liana
Vitis amurensis			liana
Vitis cordifolia			liana
Vitis riparia			liana
Vitis sinensis			liana
Vitis sp.			liana
Vitis vinifera			liana
**VIVIANIACEAE**			
Balbisia stitchkinii	shrub		
**VOCHYSIACEAE**			
Erisma bicolor		tree	
Qualea dichotoma		tree	
Qualea grandiflora		tree	
Qualea parviflora		tree	
Ruizterania albiflora		tree	
Vochysia ferruginea		tree	
Vochysia guatemalensis		tree	
**WINTERACEAE**			
Drimys piperita	shrub		
Tasmannia lanceolata	shrub		
Drimys granadensis		tree	
Drimys winteri		tree	
**XANTHORRHOEACEAE**			
Xanthorrhoea australis	shrub		
Xanthorrhoea preissii	shrub		
Xanthorrhoea resinosa		tree	
**ZYGOPHYLLACEAE**			
Bulnesia retama	shrub		
Larrea cuneifolia	shrub		
Larrea tridentata	shrub		
Zygophyllum coccineum	shrub		
Zygophyllum rosowii	shrub		
Balanites maughamii		tree	
Bulnesia arborea		tree	
Guaiacum angustifolium		tree	
Guaiacum officinale		tree	
Guaiacum sanctum		tree	
Larrea divaricata		tree	
Plectrocarpa tetracantha		tree	
